# Supplementary material for: Foundation model of electronic medical records for adaptive risk estimation
Source: Gigascience. 2025 Sep 30;14:giaf107. doi: 10.1093/gigascience/giaf107 (PMC12482913; doi:10.1093/gigascience/giaf107)
Supplement: giaf107_Supplemental_File [file giaf107_supplemental_file.pdf]

Supplementary Materials

Figure 1. Model Architecture and Hyperparameter Overview. (Left) The architecture of the transformer-based model, following the standard GPT design, includes multiple layers of masked multi-head attention and feed-forward modules, normalized at each step and combined with positional encodings. (Right) Summary of the hyperparameters used for model training and their explored ranges. The final model uses 6 layers, a context size of 2048, an embedding size of 768, 12 attention heads, a dropout rate of 0.3, and a batch size of 32. Additional information includes the percentage of discarded ambiguous inference repetitions (0.2–0.3%) that appear when doing zero-shot inference.

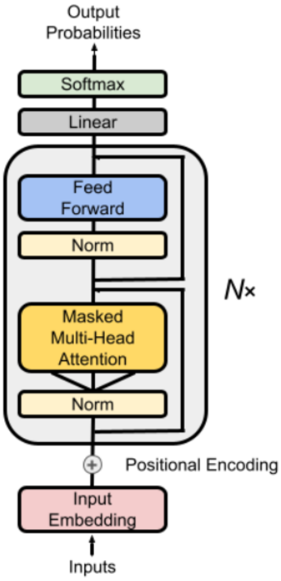

Parameter name	Parameters used	Explored
Layer number	6	4-10
Context size	2048	512-4096
Embedding size	768	512-1024
Attention head number	12	8-16
Dropout	0.3	0-0.5
Batch size	32	16-64

**Additional Information**	
Model Type	GPT without bias
Training Time	48h
Inference repetitions	>=100
Discarded ambiguous inference repetitions	0.2-0.3%

Table 1. ETHOS performance on ARES tasks with a breakdown for demographic subgroups. This table presents the predictive performance (AUROC with 95% confidence intervals) of ETHOS (top) and MEDS-Tab (bottom) for four critical clinical outcomes used in ARES: Hospital Mortality, ICU Admission, Prolonged Hospital Stay (>10 days), and a Composite Risk Score (HM+IA+PS). The prevalence rates of each outcome are provided for reference. Performance metrics are further stratified by gender and race to assess potential disparities in model performance across demographic subgroups.

	Hospital Mortality	ICU Admission	Prolonged Stay	Composite (HM+IA+PS)
Prevalence (%)	1.95	15.44	9.01	20.41
**ETHOS**				
**Overall**	0.940 [0.932, 0.947]	0.932 [0.928, 0.935]	0.853 [0.848, 0.858]	0.906 [0.902, 0.909]
**Gender**				
Female	0.944 [0.933, 0.953]	0.931 [0.927, 0.936]	0.854 [0.847, 0.862]	0.905 [0.900, 0.910]
Male	0.935 [0.924, 0.945]	0.931 [0.926, 0.936]	0.850 [0.842, 0.857]	0.906 [0.901, 0.911]
**Race**				
Asian	0.954 [0.925, 0.978]	0.944 [0.930, 0.958]	0.880 [0.858, 0.900]	0.921 [0.905, 0.936]
Black	0.955 [0.935, 0.972]	0.933 [0.924, 0.943]	0.872 [0.858, 0.885]	0.906 [0.897, 0.915]
Hispanic	0.964 [0.929, 0.987]	0.936 [0.918, 0.951]	0.881 [0.856, 0.903]	0.910 [0.893, 0.927]
Other	0.985 [0.975, 0.993]	0.956 [0.937, 0.972]	0.862 [0.831, 0.889]	0.932 [0.915, 0.949]
Unknown	0.886 [0.852, 0.911]	0.941 [0.928, 0.953]	0.790 [0.757, 0.819]	0.942 [0.928, 0.954]
White	0.928 [0.918, 0.937]	0.923 [0.919, 0.927]	0.844 [0.838, 0.851]	0.897 [0.892, 0.901]
**MEDS-Tab**				
**Overall**	0.887 [0.877, 0.897]	0.918 [0.914, 0.921]	0.815 [0.810, 0.821]	0.879 [0.875, 0.883]
**Gender**				
Female	0.898 [0.884, 0.910]	0.916 [0.910, 0.921]	0.822 [0.814, 0.830]	0.877 [0.872, 0.883]
Male	0.876 [0.861, 0.889]	0.918 [0.913, 0.922]	0.807 [0.798, 0.816]	0.878 [0.873, 0.883]
**Race**				
Asian	0.895 [0.844, 0.938]	0.916 [0.898, 0.933]	0.819 [0.786, 0.849]	0.877 [0.858, 0.897]
Black	0.918 [0.896, 0.938]	0.920 [0.909, 0.930]	0.836 [0.823, 0.851]	0.874 [0.864, 0.885]
Hispanic	0.890 [0.795, 0.954]	0.922 [0.904, 0.939]	0.851 [0.826, 0.874]	0.900 [0.882, 0.914]
Other	0.933 [0.887, 0.964]	0.943 [0.923, 0.959]	0.830 [0.800, 0.860]	0.915 [0.896, 0.933]
Unknown	0.789 [0.748, 0.829]	0.953 [0.942, 0.964]	0.750 [0.714, 0.780]	0.926 [0.910, 0.940]
White	0.871 [0.857, 0.885]	0.907 [0.903, 0.912]	0.806 [0.799, 0.814]	0.867 [0.862, 0.872]

Table 2. Demographic characteristics of the dataset analyzed in this study. The table summarizes key demographic attributes of the dataset, stratified into Train/Validation, Test, and Total splits. Patient numbers, mean age (with standard deviation), and distribution across gender, race, and marital status are shown, with percentages provided in parentheses. The data highlights the representation of each subgroup within the splits, providing context for the population characteristics in the dataset.

	Train/Validation	Test	Total
**Patient Number**	269,741	29,971	299,712
**Mean Age (Std.)**	48.5 (20.9)	48.6 (20.9)	48.5 (20.9)
**Gender (%)**			
Female	142,696 (52.9)	15,857 (52.9)	158,553 (52.9)
Male	127,045 (47.1)	14,114 (47.1)	141,159 (47.1)
**Race (%)**			
Unknown	115,437 (42.8)	12,684 (42.3)	128,121 (42.7)
White	110,408 (40.9)	12,369 (41.3)	122,777 (41.0)
Black	21,410 (7.9)	2,321 (7.7)	23,731 (7.9)
Hispanic	9,214 (3.4)	1,023 (3.4)	10,237 (3.4)
Asian	6,802 (2.5)	787 (2.6)	7,589 (2.5)
Other	6,470 (2.4)	787 (2.6)	7,257 (2.4)
**Marital Status (%)**			
Unknown	114,234 (42.3)	12,603 (42.1)	126,837 (42.3)
Married	70,269 (26.1)	7,811 (26.1)	78,080 (26.1)
Single	60,915 (22.6)	6,793 (22.7)	67,708 (22.6)
Widowed	14,243 (5.3)	1,670 (5.6)	15,913 (5.3)
Divorced	10,080 (3.7)	1,094 (3.7)	11,174 (3.7)

Table 3. Prediction of Hospitalization At Triage. Performance comparison of various models for predicting hospitalization at triage, evaluated using AUROC, AUPRC, sensitivity, and specificity (95% confidence intervals in brackets). The thresholds for sensitivity and specificity were determined by finding the operating point on the ROC curve closest to (0,1). ETHOS demonstrates superior performance across all metrics, outperforming all other methods, including traditional scoring systems and machine learning models.

	AUROC	AUPRC	Sensitivity	Specificity
LR	0.809 [0.805, 0.813]	0.775 [0.769, 0.781]	0.734 [0.721, 0.750]	0.736 [0.721, 0.749]
Med2Vec	0.816 [0.812, 0.820]	0.782 [0.775, 0.788]	0.751 [0.734, 0.770]	0.728 [0.711, 0.745]
RF	0.817 [0.814, 0.821]	0.785 [0.779, 0.791]	0.759 [0.736, 0.764]	0.726 [0.720, 0.747]
GB	0.819 [0.815, 0.823]	0.792 [0.786, 0.798]	0.753 [0.731, 0.770]	0.728 [0.715, 0.751]
MLP	0.822 [0.818, 0.826]	0.796 [0.790, 0.802]	0.754 [0.743, 0.775]	0.734 [0.716, 0.745]
esi	0.712 [0.707, 0.716]	0.632 [0.625, 0.638]	0.584 [0.577, 0.590]	0.784 [0.779, 0.789]
NEWS	0.581 [0.576, 0.586]	0.555 [0.548, 0.561]	0.563 [0.556, 0.569]	0.546 [0.540, 0.553]
NEWS2	0.565 [0.560, 0.570]	0.538 [0.532, 0.544]	0.519 [0.512, 0.526]	0.570 [0.564, 0.577]
REMS	0.666 [0.661, 0.671]	0.605 [0.598, 0.612]	0.605 [0.552, 0.722]	0.641 [0.545, 0.711]
MEWS	0.558 [0.553, 0.562]	0.521 [0.515, 0.527]	0.296 [0.289, 0.302]	0.812 [0.806, 0.817]
CART	0.673 [0.668, 0.678]	0.617 [0.610, 0.624]	0.703 [0.696, 0.709]	0.578 [0.571, 0.585]
MEDS-Tab	0.863 [0.860, 0.866]	0.879 [0.876, 0.883]	0.746 [0.735, 0.754]	0.820 [0.809, 0.835]
ETHOS (ours)	0.946 [0.944, 0.947]	0.945 [0.943, 0.947]	0.868 [0.859, 0.876]	0.864 [0.856, 0.873]

Table 4. Prediction of Critical Outcome Within 12h At Triage. Performance comparison of various models for predicting critical outcomes within 12 hours of triage, evaluated using AUROC, AUPRC, sensitivity, and specificity (95% confidence intervals in brackets). The thresholds for sensitivity and specificity were determined by finding the operating point on the ROC curve closest to (0,1). ETHOS achieves the highest performance across most of the metrics, substantially outperforming all other methods, including traditional scoring systems and machine learning models.

	AUROC	AUPRC	Sensitivity	Specificity
LR	0.875 [0.868, 0.882]	0.308 [0.288, 0.328]	0.813 [0.792, 0.836]	0.782 [0.766, 0.803]
Med2Vec	0.880 [0.872, 0.887]	0.324 [0.305, 0.346]	0.817 [0.799, 0.852]	0.787 [0.762, 0.804]
RF	0.881 [0.873, 0.888]	0.362 [0.343, 0.386]	0.812 [0.794, 0.829]	0.792 [0.788, 0.796]
GB	0.891 [0.884, 0.897]	0.389 [0.367, 0.412]	0.836 [0.804, 0.848]	0.788 [0.779, 0.812]
MLP	0.892 [0.886, 0.898]	0.372 [0.352, 0.396]	0.845 [0.806, 0.855]	0.784 [0.780, 0.823]
esi	0.821 [0.814, 0.829]	0.190 [0.178, 0.201]	0.900 [0.887, 0.913]	0.637 [0.632, 0.642]
NEWS	0.637 [0.624, 0.651]	0.139 [0.127, 0.154]	0.461 [0.440, 0.483]	0.796 [0.792, 0.801]
NEWS2	0.622 [0.610, 0.636]	0.130 [0.118, 0.144]	0.416 [0.402, 0.605]	0.822 [0.533, 0.826]
REMS	0.672 [0.661, 0.683]	0.093 [0.087, 0.102]	0.662 [0.642, 0.683]	0.602 [0.597, 0.607]
MEWS	0.623 [0.611, 0.634]	0.101 [0.093, 0.110]	0.445 [0.424, 0.466]	0.772 [0.768, 0.776]
CART	0.699 [0.687, 0.710]	0.134 [0.123, 0.147]	0.579 [0.557, 0.600]	0.720 [0.716, 0.725]
MEDS-Tab	0.853 [0.846, 0.861]	0.513 [0.493, 0.531]	0.735 [0.717, 0.752]	0.764 [0.759, 0.771]
ETHOS (ours)	0.945 [0.941, 0.950]	0.696 [0.678, 0.712]	0.876 [0.860, 0.898]	0.873 [0.852, 0.889]

Table 5. Prediction of Emergency Department Re-presentation Within 72h. Performance comparison of various models for predicting emergency department re-presentation within 72 hours, evaluated using AUROC, AUPRC, sensitivity, and specificity (95% confidence intervals in brackets). The thresholds for sensitivity and specificity were determined by finding the operating point on the ROC curve closest to (0,1). ETHOS demonstrates superior performance, outperforming all other methods and showcasing its effectiveness for this challenging task.

	AUROC	AUPRC	Sensitivity	Specificity
LR	0.679 [0.661, 0.697]	0.161 [0.141, 0.185]	0.562 [0.544, 0.645]	0.699 [0.613, 0.718]
Med2Vec	0.621 [0.601, 0.640]	0.128 [0.110, 0.148]	0.560 [0.477, 0.595]	0.615 [0.568, 0.725]
RF	0.673 [0.655, 0.691]	0.150 [0.131, 0.173]	0.642 [0.549, 0.665]	0.599 [0.594, 0.693]
GB	0.697 [0.680, 0.714]	0.165 [0.143, 0.188]	0.623 [0.592, 0.704]	0.662 [0.582, 0.690]
MLP	0.693 [0.675, 0.711]	0.168 [0.147, 0.192]	0.603 [0.579, 0.676]	0.675 [0.607, 0.701]
LSTM	0.689 [0.671, 0.708]	0.164 [0.143, 0.186]	0.595 [0.566, 0.653]	0.680 [0.633, 0.722]
MEDS-Tab	0.714 [0.696, 0.731]	0.189 [0.167, 0.214]	0.645 [0.575, 0.689]	0.657 [0.617, 0.742]
ETHOS (ours)	0.745 [0.728, 0.762]	0.214 [0.190, 0.239]	0.669 [0.611, 0.698]	0.685 [0.657, 0.757]

Table 6. Summary of Token and Timeline Statistics. This table presents a comprehensive overview of the token and timeline data in the training, test, and combined datasets. Key metrics include the total number of tokens and timelines, along with statistics on timeline lengths such as the longest timeline, median, mean, and shortest timeline. The number of unique timeline tokens is also reported. The final section breaks down the encoding of timeline tokens into categories, such as time intervals, quantiles, medications, diagnoses, procedures, laboratory results, vitals, and other clinical features. This summary highlights the diversity and complexity of the tokenized data used in the study.

	Train/Validation	Test	Total
**Tokens**	324,667,250	36,325,697	360,992,947
**Timelines**	257,081	28,539	285,620
**Timeline Lengths**			
Longest	221,346	107,147	221,346
Q3	1,053	1,063	1,055
Median	331	340	332
Mean	1,262	1,272	1,263
Q1	122	123	122
Shortest	2	2	2
Unique	13,244	4,989	13,791
**Unique Timeline Tokens**	4,495	3,947	4,495
**Timeline Tokens Encoding**			
Time Intervals	19	19	19
Quantiles	10	10	10
Medications	312	275	312
Diagnoses	2,989	2,542	2,989
Procedures	34	34	34
Labs	200	200	200
Vitals	6	6	6
HCPCS	66	37	66
Inpatient Stays	29	29	29
Emergency Department	6	6	6
DRGs	772	737	772
BMI	10	10	10

Figure 2. ROC Curves for ETHOS Across All Prediction Tasks. ROC curves and corresponding area under the curve (AUC) values with 95% confidence intervals are shown for seven prediction tasks: Hospital Mortality, ICU Admission, Prolonged Stay (>10 days), Composite Outcome (Hospital Mortality + ICU Admission + Prolonged Stay), Hospitalization at Triage, Critical Outcome Within 12h at Triage, and Emergency Department (ED) Re-presentation Within 72h. Each plot includes the fitted ROC curve (orange), unique thresholds (crosses), and the 95% confidence interval (gray shading). ETHOS demonstrates high predictive performance across all tasks, with AUC values ranging from 0.740 (ED Re-presentation) to 0.936 (Hospital Mortality).

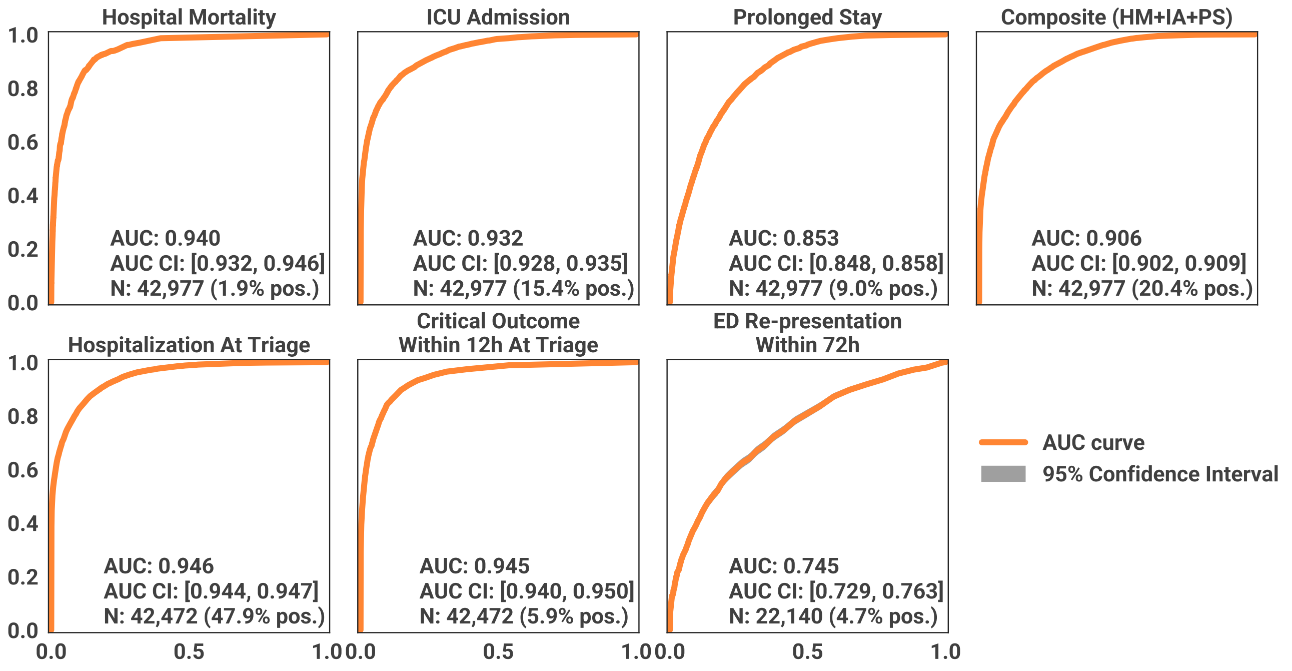

Table 7. Overview of the data sources and their corresponding columns used in this work from the MIMIC-IV database and its extension MIMIC-IV-ED. The table groups the data into three main categories: ED (Emergency Department), hosp (Hospital), and ICU (Intensive Care Unit). For each category, the associated tables and the specific columns extracted for the study are listed, highlighting key variables relevant to patient care and outcomes, such as identifiers (e.g., stay_id, hadm_id), timestamps (e.g., intime, charttime), and clinical observations (e.g., vitalsign, labresults). These selections were guided by the objectives of the study to comprehensively model patient trajectories and outcomes.

Data Source	Used Columns
**ed**	
diagnosis	icd_code, icd_version, stay_id
edstays	arrival_transport, disposition, hadm_id<br>intime, outtime, stay_id
pyxis	charttime, name, stay_id
triage	acuity, dbp, heartrate<br>o2sat, pain, resprate<br>sbp, stay_id, temperature
vitalsign	charttime, dbp, heartrate<br>o2sat, pain, resprate<br>sbp, stay_id, temperature
**hosp**	
admissions	admission_location, admission_type, admittime<br>discharge_location, dischtime, hadm_id<br>insurance, marital_status, race
diagnoses_icd	hadm_id, icd_code, icd_version
drgcodes	description, drg_code, drg_type<br>hadm_id
emar	charttime, emar_id, event_txt<br>hadm_id, medication
hcpcsevents	chartdate, hadm_id, short_description
labevents	charttime, hadm_id, itemid<br>valuenum, valueuom
omr	chartdate, result_name, result_value
patients	dod, gender
procedures_icd	chartdate, hadm_id, icd_code<br>icd_version
transfers	careunit, eventtype, hadm_id<br>intime
**icu**	
icustays	first_careunit, hadm_id, intime<br>last_careunit, outtime, stay_id

Table 8. Side-by-side view of selected columns from the original sample tables (sourced from MIMIC-IV-DEMO) compared with the format of the tokenized timelines in ETHOS.

**Original data**			**Tokenized data**		
**patients.csv**			**timeline.csv**		
subject_id	gender	anchor_age	subject_id	time	code
10038081	F	63	10000248	2192-11-29 18:44:00	ED_REGISTRATION
10019917	M	44	10000248	2192-11-29 18:44:00	ED_TRANSPORT//WALK_IN
10019568	F	59	10000248	2192-11-29 18:44:00	ED_ACUITY
10031404	F	82	10000248	2192-11-29 18:44:00	Q2
10008287	F	43	10000248	2192-11-29 19:03:00	15m-45m
**admissions.csv**			10000248	2192-11-29 19:03:00	LAB//51146//%
subject_id	hadm_id	admittime	10000248	2192-11-29 19:03:00	Q3
10035631	22732862	2112-11-10 15:55:00	10000248	2192-11-29 19:03:00	LAB//51200//%
10020786	23488445	2189-06-09 12:45:00	10000248	2192-11-29 19:03:00	Q7
10020187	26842957	2170-02-24 00:00:00	10000248	2192-11-29 19:03:00	LAB//51221//%
10005866	27167814	2148-03-10 16:16:00	10000248	2192-11-29 19:03:00	Q4
10002428	28676446	2157-07-16 04:09:00	10000248	2192-11-29 19:03:00	LAB//51222//G/DL
**diagnoses.csv**			10000248	2192-11-29 19:03:00	Q5
subject_id	hadm_id	icd_code	10000248	2192-11-29 19:03:00	LAB//51144//%
10016742	29281842	N179	10000248	2192-11-29 19:04:00	Q6
10023117	21607814	I428	10000248	2192-11-29 19:37:00	15m-45m
10040025	25933959	I130	10000248	2192-11-29 19:37:00	HOSPITAL_ADMISSION
10014354	27494880	Z955	10000248	2192-11-29 19:37:00	OBSERVATION
10014354	27487226	I5033	10000248	2192-11-29 19:37:00	INSURANCE_MEDICAID

Table 9. Estimated energy consumption of training ETHOS compared to training large language models. ETHOS is a dedicated model specifically designed for the electronic health records (EHR) domain, which allows it to be substantially smaller and more efficient to train than general-purpose large language models. With only 45 million parameters, ETHOS was trained on 8 A100 GPUs over 46 hours, consuming an estimated 220 kWh of energy. In contrast, universal LLMs such as GPT-3 (175B parameters), LLaMA 3 (8B), and Falcon (40B) require orders of magnitude more compute and energy, consuming between 307,000 and over 1.2 million kWh.

Model Name	Params (B)	GPUs Used	Duration	Est. GPU-hours	Est. Energy (kWh)
ETHOS	0.045	8×A100	46 hours	368	~220
GPT-3	175	1024×V100	~34 days	~835,000	~1,287,000
LLaMA 3 (8B)	8	~512×A100	~25 days	~307,000	~490,000
Falcon (40B)	40	384×A100	~21 days	~193,000	~307,000

Figure 3. AUPRC Curves for ETHOS Across All Prediction Tasks. Precision-recall (PR) curves and corresponding area under the precision-recall curve (AUPRC) values are shown for seven prediction tasks: Hospital Mortality, ICU Admission, Prolonged Stay (>10 days), Composite Outcome (Hospital Mortality + ICU Admission + Prolonged Stay), Hospitalization at Triage, Critical Outcome Within 12h at Triage, and Emergency Department (ED) Re-presentation Within 72h. Each plot includes the PR curve (orange) and unique thresholds (crosses). ETHOS shows good precision-recall performance across several tasks, with AUPRC values ranging from 0.199 (ED Re-presentation) to 0.887 (Hospitalization at Triage), reflecting the class imbalance present in each task.

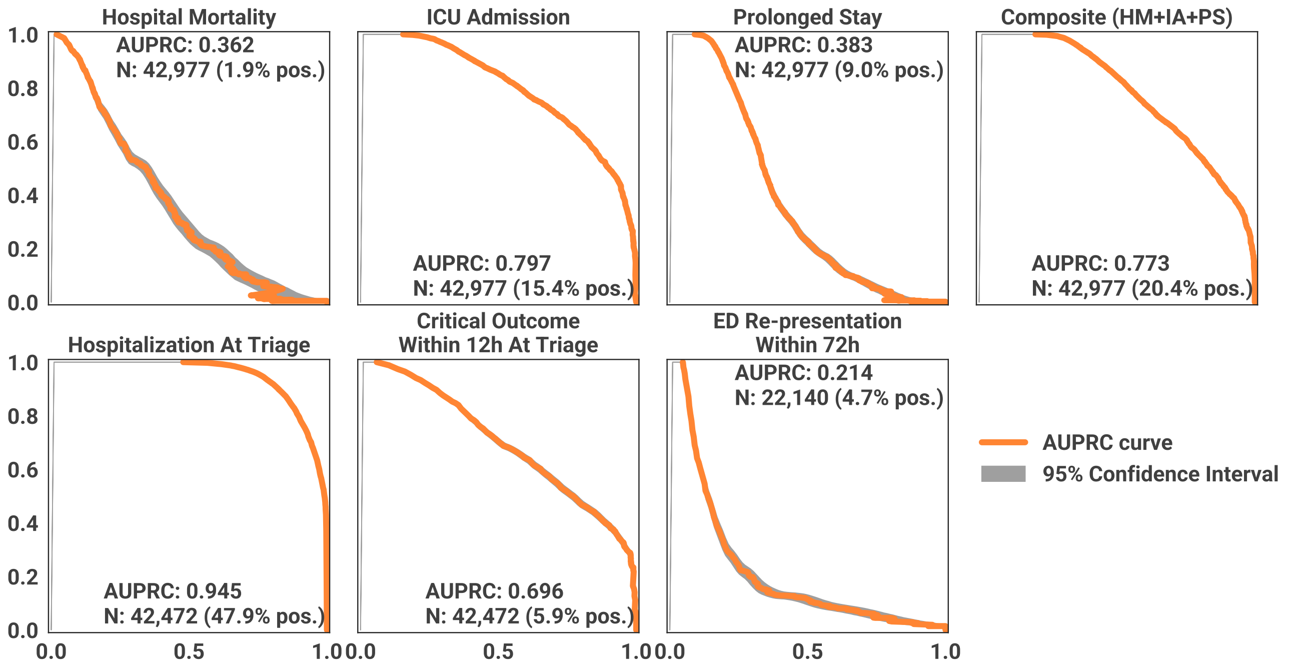

Figure 4. Calibration Curves for ETHOS Predictions Across Clinical Outcomes with 95% Confidence Intervals Determined by Bootstrapping. This figure presents calibration curves evaluating the reliability of ETHOS probability predictions across six key clinical outcomes: hospital mortality, ICU admission, prolonged hospital stay, composite risk score (HM+IA+PS), hospitalization at triage, critical outcome within 12 hours at triage, and ED re-presentation within 72 hours. The calibration curves compare predicted probabilities (x-axis) against observed event frequencies (y-axis), with perfect calibration represented by the dashed diagonal line, while the solid orange line shows ETHOS calibration performance, and the shaded gray region represents the 95% confidence interval (CI) derived from bootstrapping. Each plot includes the Brier score, a metric assessing probabilistic prediction accuracy, where lower values indicate better calibration, with 0.00–0.05 classified as excellent, 0.05–0.10 as good, 0.10–0.20 as acceptable, and values above 0.20 as poor calibration. ETHOS demonstrates excellent calibration for hospital mortality (Brier score: 0.014), critical outcome within 12 hours (0.031), and ED re-presentation (0.041), while ICU admission (0.064), prolonged stay (0.067), and the composite risk score (0.090) exhibit good calibration, closely following the ideal calibration curve. Hospitalization at triage (0.143) is categorized as acceptable calibration, with some deviations at higher predicted probabilities, suggesting areas for potential improvement. Overall, ETHOS exhibits strong calibration across most clinical tasks, particularly in predicting mortality, early critical deterioration, and ED re-presentation, with acceptable performance for hospitalization risk at triage. These findings highlight ETHOS's reliability in translating probability estimates into clinically meaningful risk stratifications, supporting its potential as a robust AI-driven decision support tool for real-time risk prediction and clinical decision-making.

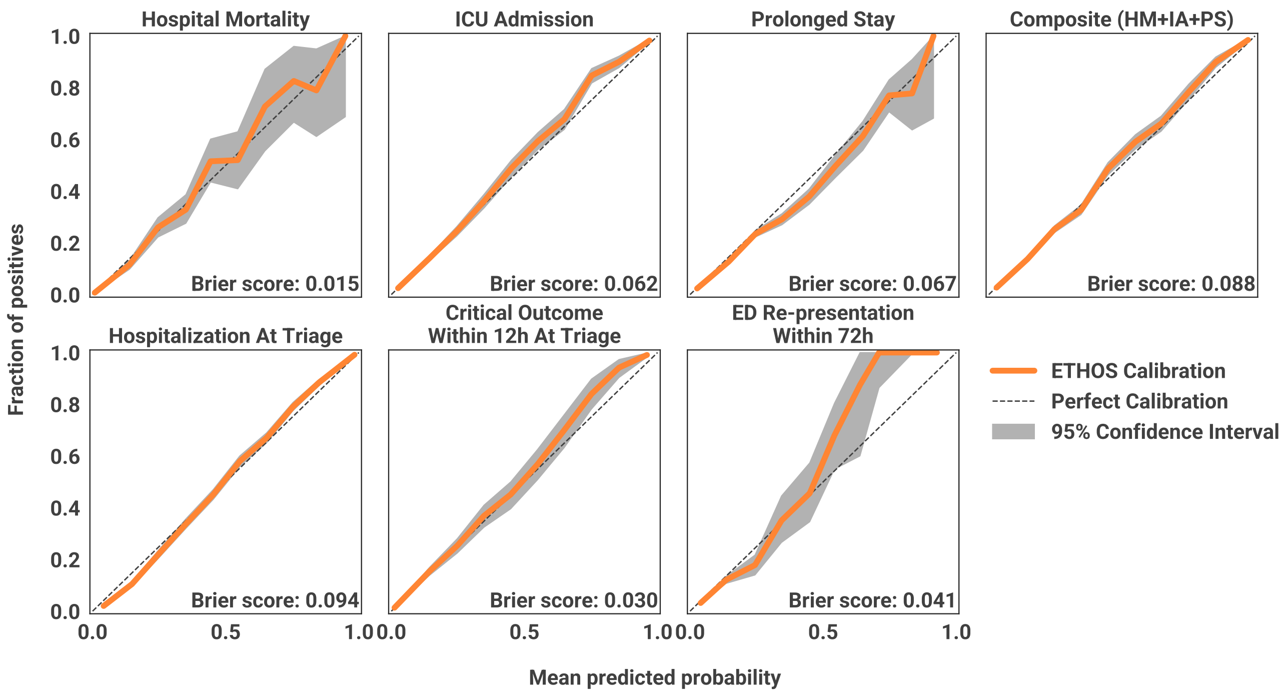

Table 10. Risk Trajectories for Eight Patients from ED Presentation to Discharge, ICU Admission, or Death.. This figure presents examples of risk trajectories for eight different patients, illustrating the dynamic evolution of risk predictions following presentation at the emergency department (ED). Each risk value is estimated from multiple (N=100) simulated fPHTs. The shaded area around each risk curve represents the 95% confidence interval (CI) for the predicted risk. The primary graphs plot risk progression as a function of the number of tokens generated since ED presentation, effectively modeling the temporal evolution of patient risk. The visualisation of ARES score is schematically represented below using 10 color-coded symbols corresponding to key risk categories (see Figures 1 and 2 in main paper). In some graphs, symbols corresponding to ICU admission risk are absent (e.g., E, F, G, and H) because these patients were already admitted to the ICU earlier, leading ARES to automatically exclude this risk component from consideration. The time axis under ARES represents actual elapsed time (in hours and days) since ED presentation. However, time progression on these axes is not linear, as the number of generated tokens does not directly correspond to real-time intervals. Instead, token generation occurs in discrete units determined by patient events. Notably, in case H, a sudden drop in prolonged stay risk occurs because ARES automatically reclassifies a risk of prolonged stay >10 days into prolonged stay >15 days, leading to an observed risk reduction. This drop is an inherent property of ARES modeling rather than a true change in patient status. All trajectories ultimately conclude when the patient either dies or is discharged.

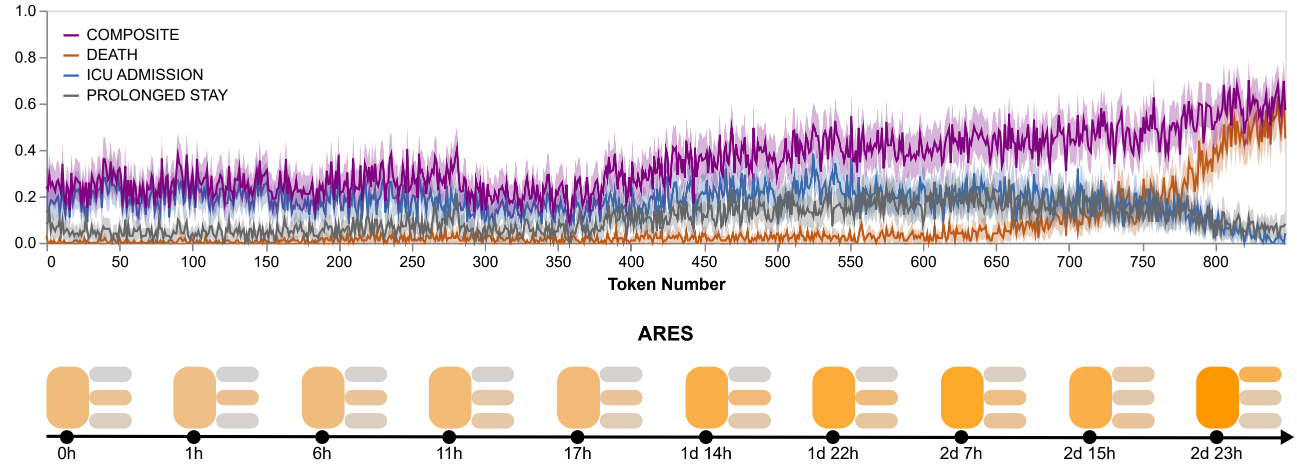

(A) Ends with death.

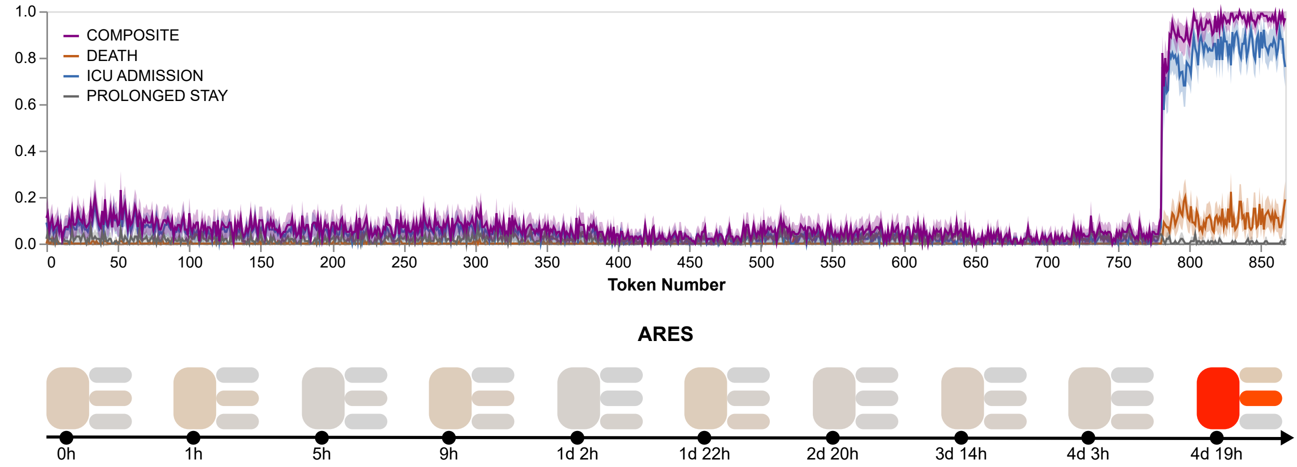

(B) Ends with ICU admission.

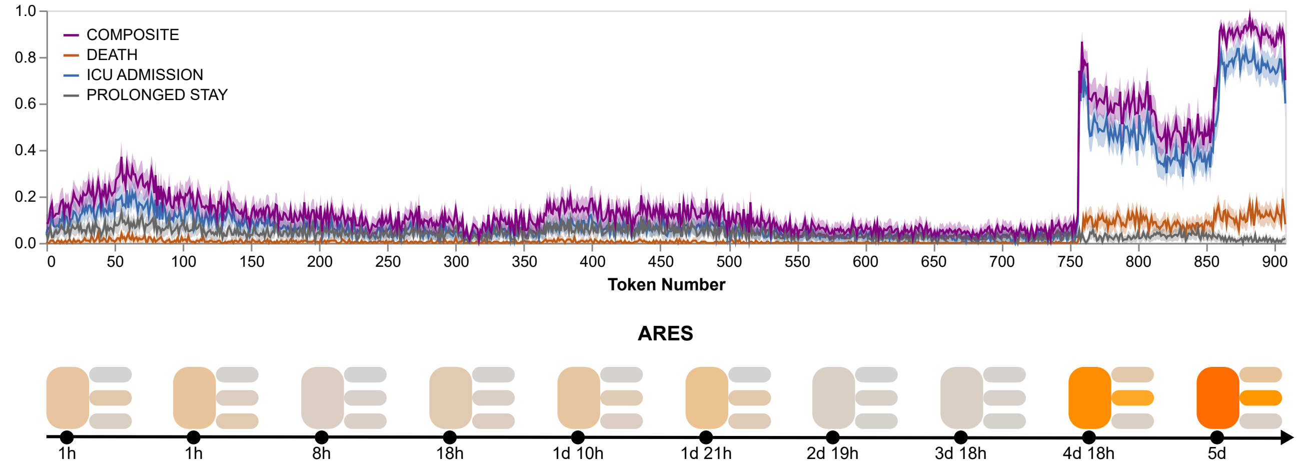

(C) Ends with ICU admission.

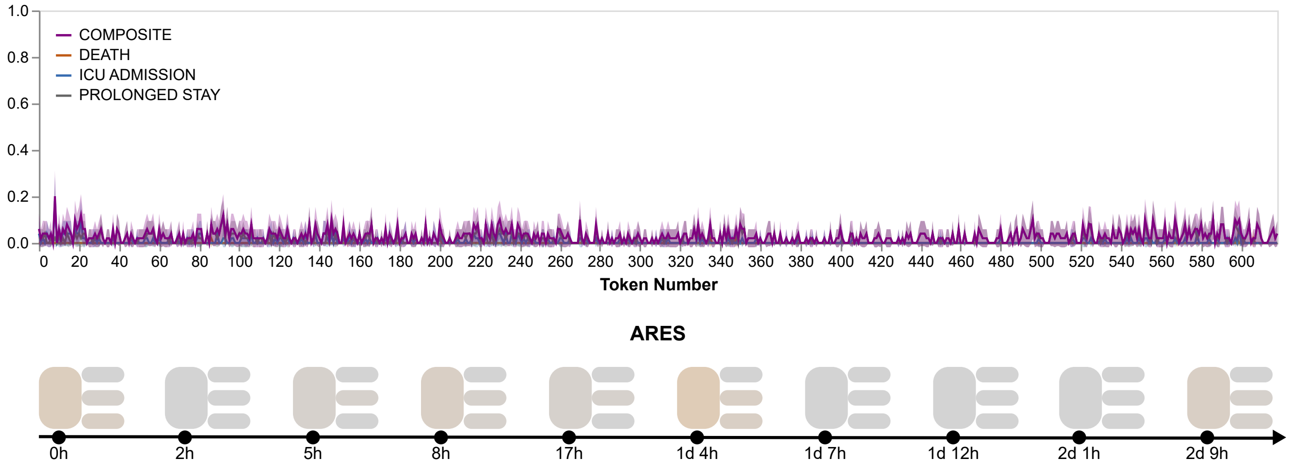

(D) Ends with hospital discharge.

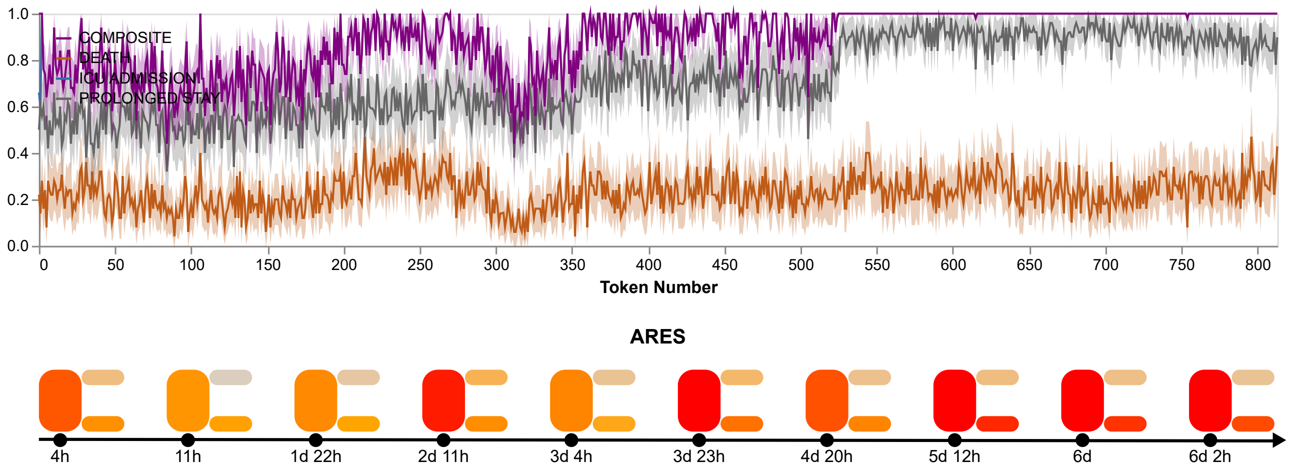

(E) Ends with death.

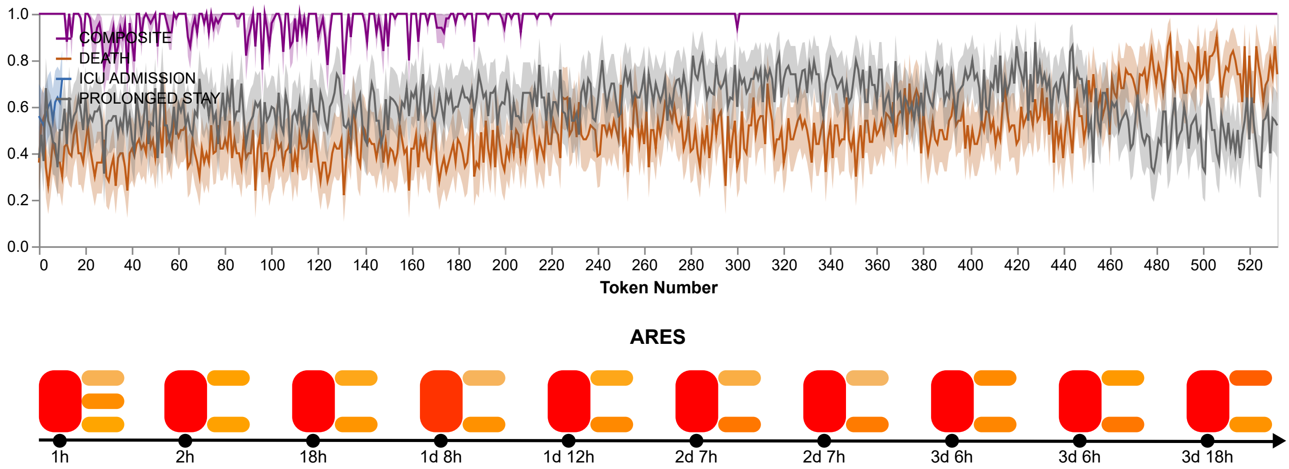

(F) Ends with death.

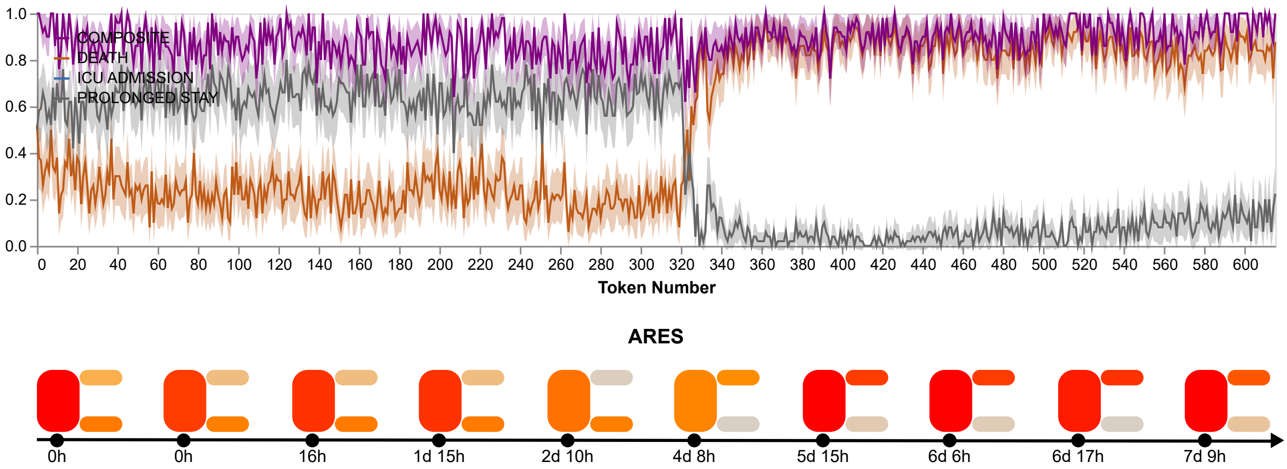

(G) Ends with death.

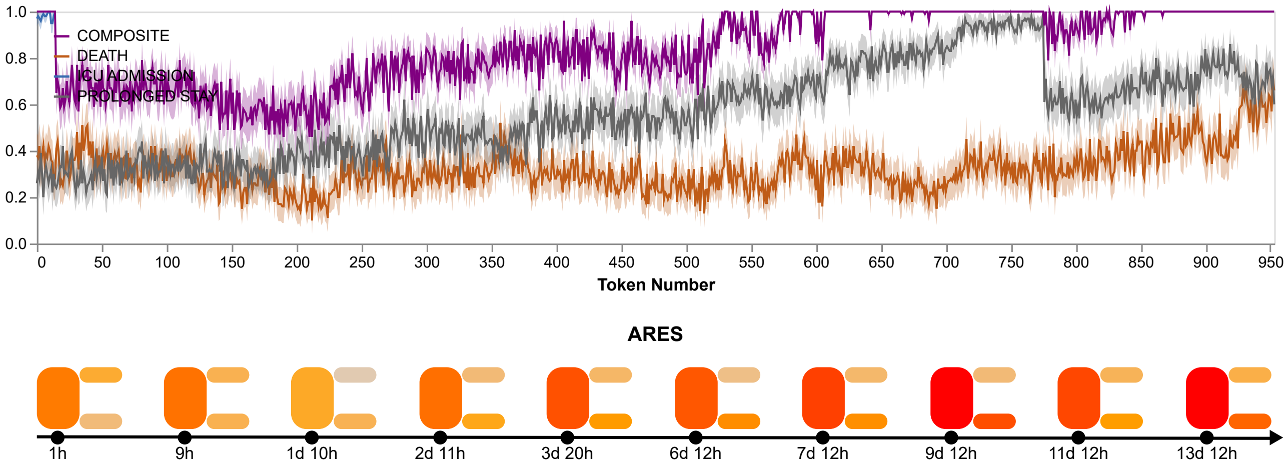

(H) Ends with death.

Table 11. Detailed Token Statistics. The table provides a detailed breakdown of the total number of tokens and unique tokens for each code group in the training, test, and combined datasets. Each code group represents a specific type of information, such as laboratory results (LAB), clinical classifications (e.g., ATC, ICD_CM), time intervals (e.g., 15m-45m, 12h-18h), and other key features like BMI, vitals, or discharge locations. The statistics summarize the diversity (#Unique) and frequency (Count) of tokens across datasets, offering insights into the distribution and variability of features used in the modeling process.

	Train		Test		Total	
	#Unique	Count	#Unique	Count	#Unique	Count
Code Group						
LAB	200	90,250,118	200	10,098,515	200	100,348,633
ATC	87	26,773,380	81	2,997,648	87	29,771,028
ATC_4	12	26,773,367	12	2,997,644	12	29,771,011
ATC_SFX	213	26,658,727	182	2,984,558	213	29,643,285
Q1	1	13,313,065	1	1,476,714	1	14,789,779
Q2	1	12,153,214	1	1,353,936	1	13,507,150
Q3	1	11,631,525	1	1,299,028	1	12,930,553
Q4	1	10,483,733	1	1,172,049	1	11,655,782
Q5	1	10,315,908	1	1,156,166	1	11,472,074
Q6	1	10,154,034	1	1,141,348	1	11,295,382
VITAL	6	9,946,752	6	1,113,072	6	11,059,824
Q7	1	9,574,210	1	1,076,334	1	10,650,544
ICD_CM	2,989	9,330,094	2,542	1,036,475	2,989	10,366,569
Q8	1	8,954,426	1	1,006,563	1	9,960,989
Q9	1	8,593,863	1	966,320	1	9,560,183
Q10	1	7,900,178	1	888,383	1	8,788,561

Continued on next page

Code Group	Train #Unique	Train Count	Test #Unique	Test Count	Total #Unique	Total Count
ICD_PCS	34	3,998,316	34	442,617	34	4,440,933
15m-45m	1	2,234,231	1	251,165	1	2,485,396
1h15m-2h	1	2,082,216	1	232,659	1	2,314,875
2h-3h	1	1,925,854	1	214,816	1	2,140,670
3h-5h	1	1,877,497	1	209,154	1	2,086,651
45m-1h15m	1	1,678,348	1	188,677	1	1,867,025
5m-15m	1	1,549,919	1	173,374	1	1,723,293
BMI	10	1,485,790	10	169,939	10	1,655,729
5h-8h	1	1,122,479	1	124,573	1	1,247,052
8h-12h	1	980,545	1	109,975	1	1,090,520
TRANSFER	38	750,441	38	83,393	38	833,834
12h-18h	1	708,241	1	79,051	1	787,292
2mt-6mt	1	465,225	1	52,313	1	517,538
=6mt	1	456,699	1	50,085	1	506,784
30d-2mt	1	430,807	1	48,696	1	479,503
12d-20d	1	388,256	1	44,259	1	432,515
DRG	772	388,255	737	42,977	772	431,232
HOSPITAL_DISCHARGE	1	388,254	1	42,977	1	431,231
DISCHARGE_LOCATION	10	388,254	10	42,977	10	431,231
INSURANCE	3	388,254	3	42,977	3	431,231
HOSPITAL_ADMISSION	1	388,254	1	42,977	1	431,231
ADMISSION_TYPE	3	388,254	3	42,977	3	431,231
ED_REGISTRATION	1	382,614	1	42,473	1	425,087
ED_OUT	1	382,614	1	42,473	1	425,087
ED_ACUITY	1	382,614	1	42,473	1	425,087
ED_TRANSPORT	4	382,614	4	42,473	4	425,087
20d-30d	1	340,809	1	38,149	1	378,958
4d-7d	1	333,877	1	38,375	1	372,252
7d-12d	1	328,988	1	37,916	1	366,904
1d-2d	1	307,351	1	34,627	1	341,978
TIMELINE_END	1	257,082	1	28,540	1	285,622
2d-4d	1	227,549	1	25,932	1	253,481
18h-1d	1	225,224	1	25,242	1	250,466
HCPCS	66	127,052	37	13,731	66	140,783
ICU_ADMISSION	1	65,816	1	7,365	1	73,181
ICU_TYPE	9	65,816	9	7,365	9	73,181
ICU_DISCHARGE	1	65,816	1	7,365	1	73,181
SOFA	1	65,816	1	7,365	1	73,181
MEDS_DEATH	1	26,200	1	2,876	1	29,076

Monte Carlo Justification for Probability Estimation

Let $p(\mathbf{x})$ denote the probability distribution over fPHTs as modeled by ETHOS where by $\mathbf{x}$ we indicate an fPHT. Suppose we want to estimate the probability of some event A regarding the future timeline. For instance, A could be the event "the patient death when admitted" or "the patient admitted to ICU." Formally,

$$\Pr(A) = \sum_{\mathbf{x} \in A} p(\mathbf{x}),$$

where the sum is over all sequences $\mathbf{x}$ for which the event A holds (i.e., $\mathbf{x} \in A$).

A. Monte Carlo Estimator

A straightforward Monte Carlo approach to approximate $\Pr(A)$ is as follows:

i. **Draw** N i.i.d. samples $\{\mathbf{x}^{(1)}, \mathbf{x}^{(2)}, \ldots, \mathbf{x}^{(N)}\}$ from the model $p(\mathbf{x})$.
ii. **Define** an indicator function $I(\mathbf{x}^{(i)} \in A)$, which is 1 if the sample $\mathbf{x}^{(i)}$ lies in A, and 0 otherwise.
iii. **Estimate** $\Pr(A)$ by the ratio

$$\hat{\Pr}(A) = \frac{1}{N} \sum_{i=1}^{N} I(\mathbf{x}^{(i)} \in A).$$

In other words, $\widehat{\Pr}(A)$ is simply the fraction of samples whose corresponding timelines satisfy event A indicated as M/N in the text.

B. Unbiasedness

If the samples $\mathbf{x}^{(i)}$ are drawn exactly from $p(\mathbf{x})$, then for each sample,

$$\mathbb{E}[I(\mathbf{x}^{(i)} \in A)] = \Pr(\mathbf{x}^{(i)} \in A) = \Pr(A).$$

Hence,

$$\mathbb{E}\left[\widehat{\Pr}(A)\right] = \mathbb{E}\left[\frac{1}{N}\sum_{i=1}^{N} I(\mathbf{x}^{(i)} \in A)\right] = \Pr(A),$$

showing that $\widehat{\Pr}(A)$ is an *unbiased* estimator of $\Pr(A)$.

C. Convergence by the Law of Large Numbers

By the Law of Large Numbers (LLN), as $N \to \infty$,

$$\widehat{\Pr}(A) \xrightarrow{a.s.} \Pr(A),$$

meaning the simple ratio of "successes" (i.e., samples satisfying A) to total draws converges almost surely to the true probability.

Intuitive Operation of ETHOS

Figure 1 shows the decoder-only transformer backbone of ETHOS, which follows the standard GPT design. Below we provide an intuitive, step-by-step explanation of how ETHOS works "zero-shot," i.e. with no additional fine-tuning for specific prediction tasks.

1. Treating Patient History as a "Story.". Each Patient Health Timeline (PHT) is a long sequence of discrete tokens, think of each token as a "word" in a clinical narrative. Static tokens (e.g., demographics) set the scene, categorical tokens (e.g., diagnoses) are like domain-specific vocabulary, and time-interval tokens keep track of elapsed time between events. Just as a language model reads a sentence and predicts the next word, ETHOS reads a patient's history and predicts the next medical event token.

2. Zero-Shot Generative Inference.. Because ETHOS has been trained to model the joint distribution of these tokens, at inference time it can *continue* any PHT without further training. We provide the model with the tokens of a patient's past, and then let it generate new tokens one at a time. No task-specific labels or fine-tuning are needed—hence "zero-shot." Each new token corresponds to a plausible future event (e.g., a lab draw, medication change, or hospital admission).

3. Exploring Possible Futures via Sampling.. To capture uncertainty, we perform many independent "completions" of the PHT. Each completion (an fPHT) is analogous to asking, "What could happen next?" and letting the model write the next chapter of the patient's story. We use nucleus sampling (top-p) to allow diversity: at each step, ETHOS randomly selects the next token from the smallest set whose cumulative probability exceeds $p = 0.9$. By generating N such fPHTs, we build a Monte Carlo ensemble of future trajectories.

4. From Generated Tokens to Risk Estimates.. Some tokens are can be treated as outcome (e.g., mortality). To compute inpatient mortality, we simply count how many of the N simulated fPHTs include that token while patient is in hospital (before discharge token). The fraction M/N then provides a direct, probabilistic estimate of risk. This simulation-based approach naturally accounts for multiple pathways and branching possibilities in a patient's course.

5. Toward Controlled Cohort Generation.. Because ETHOS is a pure generator, one can modify the sampling distribution, reweighting certain token types (e.g., age or comorbidity tokens), to synthesize patient cohorts with desired characteristics. This capability opens the door to fairness-aware risk modeling and targeted "what-if" analyses, which we plan to explore in future work.

Baseline Models

To demonstrate the applicability of ETHOS as the base for ARES we compared its performance on three benchmark tasks against other baseline models. Those tasks were: prediction of the hospital admission at triage, prediction of the critical outcome (death or transfer to ICU within 12 hours) at triage, and ED re-presentation within 72 hours after discharge from ED. We followed the baseline models utilized for the benchmark tasks as presented in [27]. Firstly, we used clinically applied scoring systems: Modified Early Warning Score (MEWS) [13], National Early Warning Score (NEWS, versions 1 and 2) [12, 32, 33], Rapid Emergency Medicine Score (REMS) [34], and Cardiac Arrest Risk Triage (CART) [35]. Those scores require the collection of specific clinical features like Heart Rate, Respiratory Rate or Oxygen Saturation. They cannot adapt to the dynamic state of the patient unless all the features used by the scoring system are remeasured. Similarly, Emergency Severity Index (ESI) [36], a five-level triage system, was assigned by a nurse. In addition, we created custom scores with the AutoScore method that generates scoring systems from clinical features automatically in six steps, including variable ranking and transformation as well as score derivation by weighting and normalization [49]. Next, we utilized classic machine learning algorithms, including Logistic Regression (LR), which is a linear classifier, Multi-layer Perceptron (MLP), a non-linear classifier and tree-based, ensemble algorithms Random Forest (RF) and Gradient Boosting (GB). Finally, we applied more advanced deep learning-based algorithms

Med2Vec [37], which applies non-linear transformations to vector embeddings of ICD codes and Long Short-Term Memory (LSTM) [38] a neural network accounting for temporal changes in data for the ED re-presentation task. We make our adapted baseline model training code publicly available for reproducibility purposes [1].

Details of model training

ETHOS was implemented as a decoder-only transformer. Training data were constructed by appending an "End of timeline" token to each patient's PHT and concatenating all timelines into one long token sequence. The model was then trained, in an unsupervised fashion, to predict the next token given its preceding context by minimizing the standard cross-entropy loss, exactly as in large-scale language-model pretraining. Given the scale of our PHT corpus (hundreds of millions of tokens) and the model's complexity, we matched the parameter count of GPT-2 as a starting point, conducted a focused hyperparameter search (see Figure 1), and made heuristic adjustments to optimize convergence. Training was performed across eight NVIDIA GPUs over approximately 36 hours, reflecting resource requirements similar to those for open-domain transformer models. AdamW optimizer (decoupled weight decay) with hyperparameters matched to those in nanoGPT/GPT-2–scale pretraining. In our experiments, we used a constant learning rate of $4e^{-4}$, weight-decay of 0.01, and default momentum parameters (β_1 = 0.9 and β_2 = 0.999 training for 100K steps per federated round with batch size 32. For more details, refer to [15]. All code including training scripts is publicly available in our GitHub repository.

1 https://github.com/ipolharvard/mimic4ed-benchmark
