# Supplementary material for: Foundation model of electronic medical records for adaptive risk estimation
Source: Gigascience. 2025 Sep 30;14:giaf107. doi: 10.1093/gigascience/giaf107 (PMC12482913; doi:10.1093/gigascience/giaf107)
Supplement: giaf107_GIGA-D-25-00088_original_submission [file giaf107_giga-d-25-00088_original_submission.pdf]

GigaScience

Foundation Model of Electronic Medical Records for Adaptive Risk Estimation

--Manuscript Draft--

**Manuscript Number:**	GIGA-D-25-00088
**Full Title:**	Foundation Model of Electronic Medical Records for Adaptive Risk Estimation
**Article Type:**	Research
**Funding Information:**	NHLBI Division of Intramural Research (R01HL159183) — Not applicable
**Abstract:**	Background, The U.S. allocates nearly 18\% of its GDP to healthcare but experiences lower life expectancy and higher preventable death rates compared to other high-income nations. Hospitals struggle to predict critical outcomes such as mortality, ICU admission, and prolonged hospital stays. Traditional early warning systems, like NEWS and MEWS, rely on static variables and fixed thresholds, limiting their adaptability, accuracy, and personalization; Methods, We developed the Enhanced Transformer for Health Outcome Simulation (ETHOS), an AI model that tokenizes patient health timelines (PHTs) from EHRs and uses transformer-based architectures to predict future PHTs. The Adaptive Risk Estimation System (ARES) leverages ETHOS to compute dynamic, personalized risk probabilities for clinician-defined critical events. ARES also features a personalized explainability module that highlights key clinical factors influencing risk estimates. We evaluated ARES on the MIMIC-IV v2.2 dataset in emergency department settings, benchmarking its performance against traditional early warning systems and machine learning models; Results, From 299,721 unique patients, 285,622 PHTs (60\% with hospital admissions) were processed, comprising over 357 million tokens. ETHOS outperformed benchmark models in predicting hospital admissions, ICU admissions, and prolonged stays, achieving superior AUC scores. Its risk estimates were robust across demographic subgroups, with calibration curves confirming model reliability. The explainability module provided valuable insights into patient-specific risk factors; Conclusions, ARES, powered by ETHOS, advances predictive healthcare AI by delivering dynamic, real-time, personalized risk estimation with patient-specific explainability. Its adaptability and accuracy offer a transformative tool for clinical decision-making, potentially improving patient outcomes and resource allocation.
**Corresponding Author:**	Arkadiusz Sitek, Ph.D.<br>Massachusetts General Hospital<br>Sommerville, Massachusetts UNITED STATES
**Corresponding Author Secondary Information:**	
**Corresponding Author's Institution:**	Massachusetts General Hospital
**Corresponding Author's Secondary Institution:**	
**First Author:**	Pawel Renc
**First Author Secondary Information:**	
**Order of Authors:**	Pawel Renc
	Michal K Grzeszczyk
	Nassim Oufattole
	Deirdre Goode
	Yugang Jia
	Szymon Bieganski
	Matthew BA McDermott
	Jaroslaw Was

Powered by Editorial Manager® and ProduXion Manager® from Aries Systems Corporation

	Anthony E Samir
	David W Bates
	Jonathan W Cunningham
	Arkadiusz Sitek, Ph.D.
**Order of Authors Secondary Information:**	
**Additional Information:**	
**Question**	**Response**
Are you submitting this manuscript to a special series or article collection?	No
**Experimental design and statistics**<br><br>Full details of the experimental design and statistical methods used should be given in the Methods section, as detailed in our Minimum Standards Reporting Checklist. Information essential to interpreting the data presented should be made available in the figure legends.<br><br>Have you included all the information requested in your manuscript?	Yes
**Resources**<br><br>A description of all resources used, including antibodies, cell lines, animals and software tools, with enough information to allow them to be uniquely identified, should be included in the Methods section. Authors are strongly encouraged to cite Research Resource Identifiers (RRIDs) for antibodies, model organisms and tools, where possible.<br><br>Have you included the information requested as detailed in our Minimum Standards Reporting Checklist?	Yes
**Availability of data and materials**<br><br>All datasets and code on which the conclusions of the paper rely must be either included in your submission or	Yes

Powered by Editorial Manager® and ProduXion Manager® from Aries Systems Corporation

deposited in publicly available repositories (where available and ethically appropriate), referencing such data using a unique identifier in the references and in the "Availability of Data and Materials" section of your manuscript.<br><br>Have you have met the above requirement as detailed in our Minimum Standards Reporting Checklist?	
GigaScience has policies and guidelines in place for the use of generative AI-writing tools such as ChatGPT. If you have used such writing tools to assist with writing the manuscript this must be declared and cited in the text. Authors should not list AI-writing tools and other AI-assisted technologies as an author or co-author and should acknowledge that they are fully responsible for text generated or refined by AI-writing tools.<p><br><br>A summary of use (particularly in the introduction or among methods) needs to be included at the end of the paper, and the outputs should also be included as a supplementary file hosted in GigaDB or other open repositories. Please read our guidelines for more information. <p><br><br>By submitting to GigaScience, you are aware of the journal's AI-writing tools policy, and if you have declared use of such tools below, you have acknowledged this where appropriate in your manuscript and have made a summary of use and outputs available. <p><br><b>AI-assisted writing tools have been used in the preparation of this manuscript?	No

Manuscript

Click here to access/download;Manuscript;ETHOS_ARES___GigaScience_

GigaScience, 2023, 1–11

doi: xx.xxxx/xxxx
Manuscript in Preparation
Paper

PAPER

Foundation Model of Electronic Medical Records for Adaptive Risk Estimation

Pawel Renc[1,2,3], Michal K. Grzeszczyk[1,2], Nassim Oufattole[4], Deirdre Goode[8,2], Yugang Jia[4], Szymon Bieganski[6], Matthew B. A. McDermott[2], Jaroslaw Was[3], Anthony E. Samir[1,2], Jonathan W. Cunningham[5,2], David W. Bates[5,7,2] and Arkadiusz Sitek[1,2,*]

[1]Massachusetts General Hospital, Boston, USA and [2]Harvard Medical School, Boston, USA and [3]AGH University of Krakow, Krakow, Poland and [4]Massachusetts Institute of Technology, Cambridge, USA and [5]Brigham and Women's Hospital, Boston, USA and [6]Medical University of Lodz, Lodz, Poland and [7]Harvard Chan School of Public Health, Boston, USA and [8]Newton Wellesley Hospital, Newton, USA

[*] Corresponding author: Arkadiusz Sitek, sarkadiu@gmail.com

Abstract

Background, The U.S. allocates nearly 18% of its GDP to healthcare but experiences lower life expectancy and higher preventable death rates compared to other high-income nations. Hospitals struggle to predict critical outcomes such as mortality, ICU admission, and prolonged hospital stays. Traditional early warning systems, like NEWS and MEWS, rely on static variables and fixed thresholds, limiting their adaptability, accuracy, and personalization; **Methods,** We developed the Enhanced Transformer for Health Outcome Simulation (ETHOS), an AI model that tokenizes patient health timelines (PHTs) from EHRs and uses transformer-based architectures to predict future PHTs. The Adaptive Risk Estimation System (ARES) leverages ETHOS to compute dynamic, personalized risk probabilities for clinician-defined critical events. ARES also features a personalized explainability module that highlights key clinical factors influencing risk estimates. We evaluated ARES on the MIMIC-IV v2.2 dataset in emergency department settings, benchmarking its performance against traditional early warning systems and machine learning models; **Results,** From 299,721 unique patients, 285,622 PHTs (60% with hospital admissions) were processed, comprising over 357 million tokens. ETHOS outperformed benchmark models in predicting hospital admissions, ICU admissions, and prolonged stays, achieving superior AUC scores. Its risk estimates were robust across demographic subgroups, with calibration curves confirming model reliability. The explainability module provided valuable insights into patient-specific risk factors; **Conclusions,** ARES, powered by ETHOS, advances predictive healthcare AI by delivering dynamic, real-time, personalized risk estimation with patient-specific explainability. Its adaptability and accuracy offer a transformative tool for clinical decision-making, potentially improving patient outcomes and resource allocation.

Key words: Early Warning scores; EHR; Foundation Model;

Background

The United States allocates nearly 18 percent of its GDP to healthcare, yet Americans have shorter lifespans and poorer health than residents of other high-income nations. Among these countries, the U.S. not only has the lowest life expectancy but also the highest rates of preventable deaths [1]. Hospitals face mounting challenges managing patient influx and identifying individuals at risk for critical outcomes, including mortality, intensive care unit (ICU) admission, or prolonged hospital stays [2]. Accurate prediction of critical clinical events is essential for enhancing patient care and optimiz-

Compiled on: March 2, 2025.
Draft manuscript prepared by the author.

Key Points

- ETHOS enables dynamic, real-time risk estimation by predicting patient health timelines (PHTs) from electronic health records using a transformer-based model.
- ARES enhances clinical decision-making by leveraging ETHOS-generated future PHTs to provide personalized risk predictions with explainability.
- ETHOS outperforms traditional models in predicting critical outcomes while demonstrating strong calibration and equitable performance across demographic subgroups.

ing the timely allocation of limited healthcare resources [3]. Early identification of at-risk patients enables clinicians to prioritize interventions, anticipate potential escalations in care, and improve outcomes while simultaneously reducing costs [4, 5]. However, current methodologies often fail to fully utilize the vast and complex data available in electronic health records (EHRs), a limitation that becomes particularly evident in emergency settings where time-sensitive decisions are critical [6, 7, 8, 9, 10]. Traditional scoring systems, such as the National Early Warning Score (NEWS) [11] and the Modified Early Warning Score (MEWS) [12], rely on static variables and predefined thresholds, constraining their ability to adapt to dynamic and multifaceted patient data. Similarly, conventional machine learning models depend on preselected predictors of patient deterioration, requiring the inclusion of only a limited number of variables. These approaches are further hindered by their reliance on specific cutoff points for data inclusion (e.g., triage, 24-hour windows), which can overlook valuable longitudinal patterns.

Recent advances in generative machine learning, particularly in transformer architectures [13, 14, 15, 16], which underpin the success of Large Language Models (LLMs) [17, 18], have unlocked unprecedented capabilities for processing high-dimensional, heterogeneous, time-stamped, and episodic health data derived from electronic health records (EHRs) [14, 19, 20, 21, 22, 23]. In this work, we build on our previous development, the Enhanced Transformer for Health Outcome Simulation (ETHOS) [14]. While our approach shares some similarities with the works of [20] or [19] it differs in how EHR data are encoded and processed by the transformer model. ETHOS is designed to provide zero-shot predictions; once the model is trained, no additional fine-tuning is required to make inferences. The model operates on Patient Health Timelines (PHTs), which are tokenized sequences of events extracted from EHRs, including demographics, medical history, medications, and more (the full list is provided in Table S7). Using known PHTs up to a given point in time, ETHOS predicts future PHTs (Figure 1). ETHOS enables dynamic, real-time risk assessment by computing a range of possible Patient Health Timelines (PHTs) for a defined outcome, such as ICU admission. If the probability of an adverse event exceeds a critical threshold, appropriate interventions can be initiated to mitigate risk. This continuous probability estimation functions as an early warning system, similar to an experienced physician's intuition in identifying high-risk patients. Unlike traditional models that require predefined inference tasks, ETHOS operates as a single, unified model, allowing for the simultaneous assessment of multiple positive and negative outcomes without retraining. Probabilities for various clinical events are dynamically updated as new patient data becomes available, ensuring adaptability throughout the care process. We refer to this flexible and scalable risk prediction framework as the Adaptive Risk Estimation System (ARES), as illustrated in Figure 2.

In this paper, we present the development of ARES and introduce a novel explainability framework that delivers fully personalized insights, allowing clinicians to understand the specific factors influencing the system's risk predictions for individual patients. We benchmark the performance of ARES against state-of-the-art methods across multiple clinically relevant tasks, demonstrating its superior predictive accuracy. Using Emergency Department (ED) datasets from MIMIC-IV-ED [24, 25, 26], we validate its effectiveness and provide the accompanying code for the full reproduction of all the experiments by other researchers.

Data Description

In this study, we used the Medical Information Mart for Intensive Care (MIMIC-IV) version 2.2 database [24, 25], including its ED extension. MIMIC-IV, developed by the Massachusetts Institute of Technology and Beth Israel Deaconess Medical Center contains de-identified health records for almost 300,000 patients either admitted to the ED and/or hospital at BIDMC from 2008 to 2019. Detailed patient demographics are presented in Table S2.

Analyses

Following the tokenization process, the data of 299,721 unique patients from the MIMIC-IV dataset was converted into 285,622 PHTs, which were subsequently used for training and testing. The discrepancy arises from some patients lacking associated data after tokenization. Of the total PHTs, approximately 60% (180,733) contained hospital admissions records. The tokenized dataset comprised over 357 million tokens in total. Detailed information regarding the MIMIC-IV data used, patient demographics, characteristics of the PHTs and tokens, and descriptive statistics are provided in supplementary data (Table S2,S7,S6,S9). The model was trained and validated on 90% of the PHTs, with the remaining 10% reserved for testing. During inference, at least 100 fPHTs were generated for each investigated task.

The predictive performance of ETHOS and MEDS-Tab was evaluated for four critical clinical outcomes: hospital mortality, ICU admission, prolonged hospital stay, and a composite risk score (HM+IA+PS). Prolonged stay was defined as a stay longer than 90th percentile of all stays. All predictions were performed at patient admission. As summarized in Figure 5, 3, and Table S1, ETHOS consistently outperformed MEDS-Tab across all outcome measures, demonstrating superior AUC values. Notably, ETHOS yielded higher AUC values across all racial groups, with the most significant improvement observed among Asian and Hispanic patients. The model's robustness across diverse populations suggests its potential for mitigating disparities in predictive accuracy.

Figure 4 illustrates the dynamic risk trajectories generated by ARES, showcasing how the system continuously updates probability estimates for key clinical outcomes, including ICU admission, prolonged hospital stay, and mortality, as new clinical events occur. The figure highlights specific medical interventions, such as laboratory tests and procedures, that drive significant changes in risk estimates, demonstrating ARES's ability to integrate evolving patient data into real-time risk assessment. The results underscore the model's capacity to capture complex temporal relationships between clinical events, dynamically recalibrating risk scores based on patient status and treatment progression.

In addition to risk which are part of ARES and to contextualize

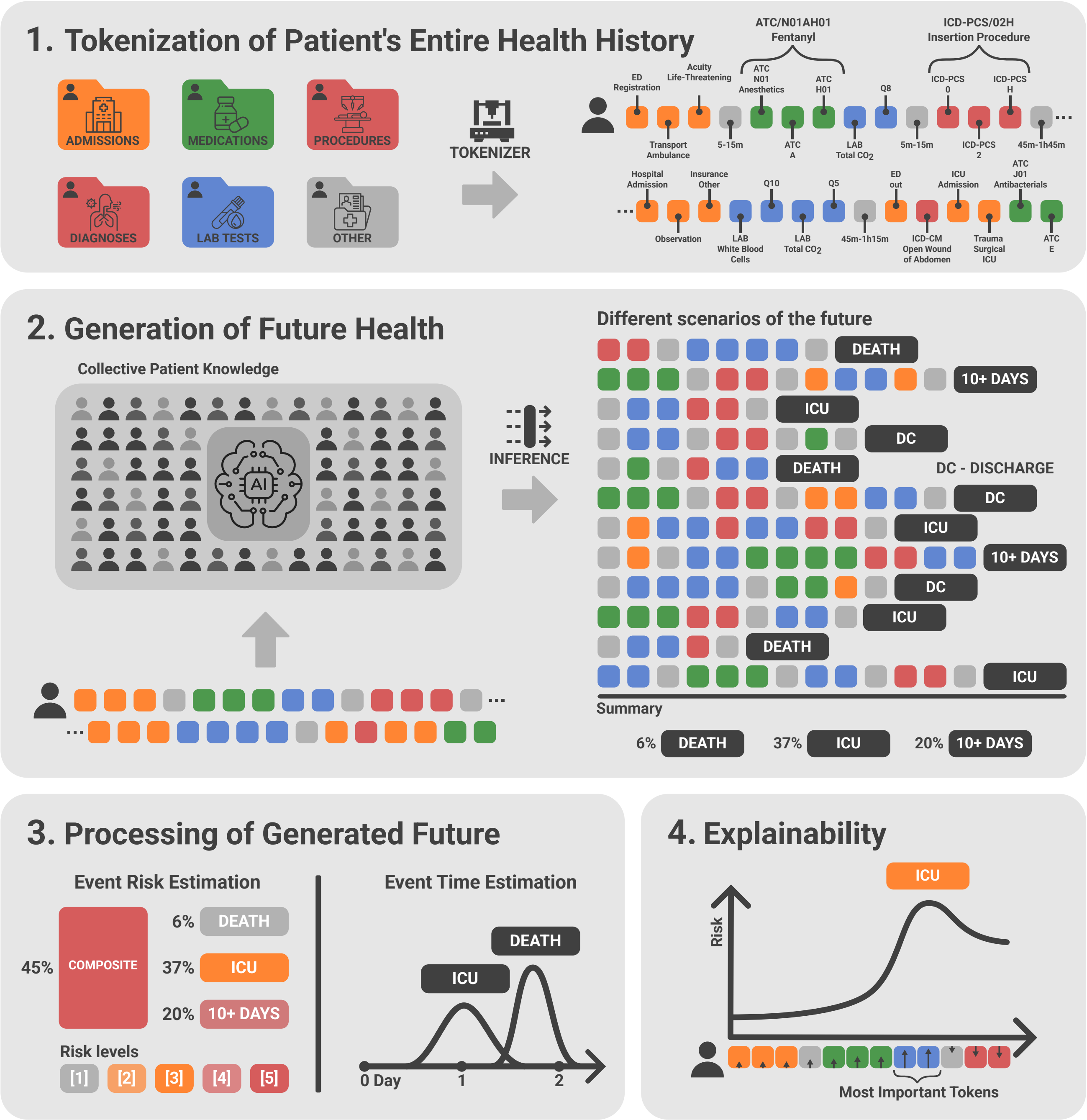

Figure 1. Workflow of the Adaptable Risk Estimation Score (ARES) Framework. This figure illustrates the ARES framework, developed on the ETHOS model, for dynamic and explainable risk evaluation. Panel 1 depicts the tokenization of a patient's entire health history into structured events represented as a sequence of tokens (PHTs), incorporating standardized coding systems such as ATC for medications, ICD-PCS for procedures, and others. Panel 2 demonstrates how the ETHOS model trained on a large dataset of PHTs to simulate potential future patient health timelines (fPHTs). By analyzing a particular patient's known PHT and generating multiple fPHTs, the model estimates the probabilities of critical outcomes, such as inpatient death, ICU admission, or a prolonged hospital stay exceeding 10 days. Panel 3 showcases the result of processing of fPHTs to calculate event-specific risks and predict the timing of these events, should they occur. Risk levels are defined across five categories, color-coded for enhanced clinical interpretability. Panel 4 showcases the explainability module, which identifies the key factors influencing specific risk estimates, offering personalized and actionable insights to support clinical decision-making. In this example, blue tokens indicate factors contributing to an increased risk of ICU admission.

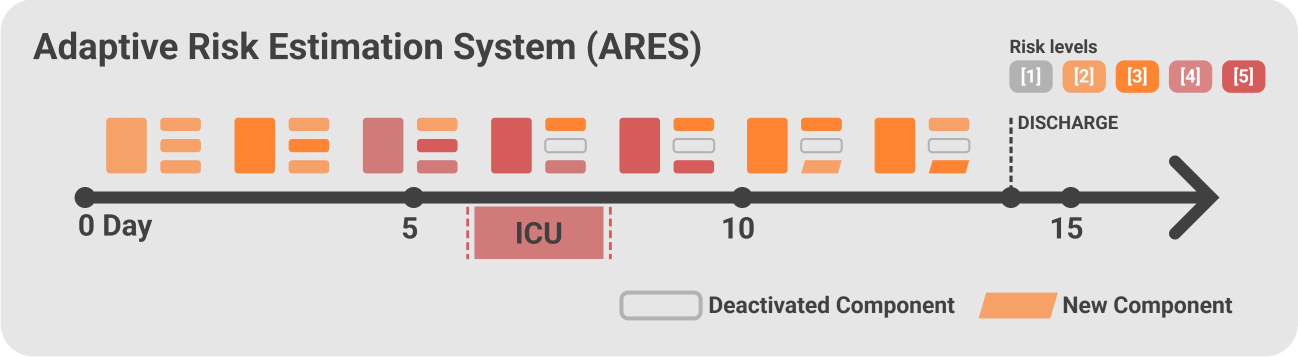

Figure 2. Timeline of a Patient's Hospital Stay and Hypothetical Risk Predictions by ARES. This figure illustrates the timeline of a patient's hospital stay, from admission to discharge around Day 14, demonstrating how ARES dynamically adjusts its predictions based on the patient's evolving clinical status and medical history. By Day 5, ARES predicts a high risk of ICU admission, which is subsequently confirmed as the patient is admitted around Day 6. Once the patient is in the ICU, ARES discontinues ICU risk evaluation, as indicated by the "Deactivated Component" label. After the ICU stay, ARES identifies an increased likelihood of a hospital stay exceeding 10 days. Upon reaching the 10-day threshold, ARES automatically recalibrates its predictions, replacing the previous risk estimation with the likelihood of a 15-day stay, now categorized as a "New Component" in the risk assessment.

the predictive capabilities of ETHOS, we compared its performance against traditional early warning scores and other ML models. Figure 3 presents the AUC values (ROC curves in Figure S2) for key ED benchmark tasks: hospitalization at triage, critical outcomes within 12 hours of triage, and ED re-presentation within 72 hours post-discharge. ETHOS demonstrated consistently superior predictive accuracy across all evaluated tasks. We provide detailed numerical values in supplementary data (Table S3,S4,S5).

The risks provided by ETHOS were also found to be well-calibrated, as tested by calibration curves. Brier scores were found in the range 0.01-0.14 depending on the task, indicating excellent to good performances, as shown in Figure S3.

Discussion

The ARES framework introduces an innovative approach to building predictive models by leveraging cutting-edge artificial intelligence technology. Several aspects of this approach distinguish it from traditional models. First, ARES enables dynamic risk estimation at any time during a patient's stay, from admission to discharge. Powered by ETHOS [14], ARES utilizes PHTs and incorporates all available clinical information at the time of risk estimation. Unlike traditional models, which rely on static data points such as information collected within 24 hours after admission or ED presentation or data up to triage [27, 28], ARES continuously adapts to the patient's evolving clinical status. This adaptability overcomes a key limitation of static models, which may not perform optimally outside the narrow time frames for which they are designed. This capability is demonstrated in the accompanying Figure 4 and Table S8, which illustrate how risk evolves over time during a patient's hospital stay. These visualizations, which depict how personalized risk evolves over time to reach the current estimates, provide insights into the specific factors driving model predictions for each patient. They highlight clinical events associated with increased or decreased risk, offering real-time explainability. By identifying the most influential features contributing to an individual's risk assessment, ARES empowers clinicians with a clearer understanding of the rationale behind each prediction.

As illustrated in Figure 1, ARES can estimate risk for various critical events, such as in-hospital mortality, ICU admission, and prolonged hospital stays. Beyond these standard metrics, additional indicators can be integrated seamlessly, including the risk of ICU admission during a specific length of stay, ICU readmission, acute kidney injury, sepsis, cardiac arrest, or 30-day readmission, and others. The ETHOS model, which underpins ARES, allows for the dynamic combination of these risks into composite measures while accounting for their interdependencies. For example, the occurrence of mortality on Day 8 would render the probability of a 10-day hospital stay zero. This ability to incorporate conditional and causal relationships between tracked events is another strength of ARES. Importantly, integrating additional metrics does not require model retraining or modifications to the ETHOS model. Once a range of possible future PHTs has been generated, any additional metrics can be calculated with minimal computational resources, making ARES scalable and adaptable to diverse healthcare settings.

In its current implementation, ETHOS distills multiple fPHTs into a single predictive decision, such as inpatient mortality. However, this approach overlooks the wealth of longitudinal information contained in these trajectories, including the sequence of clinical events that lead to a particular outcome, or the absence thereof. By merely predicting the likelihood of an adverse event, valuable insights into the pathways that contribute to deterioration or recovery remain underutilized. Expanding ETHOS to provide a more granular, trajectory-based interpretation of risk would allow clinicians not only to assess a patient's probability of experiencing a critical event but also to understand the evolving clinical course leading to that outcome including the cost. This enhanced approach would address a key limitation highlighted in the early warning paradox [29], where models trained on retrospective data may fail to capture the full complexity of clinical interventions and their effects on patient outcomes. Moving forward, we aim to refine ETHOS to incorporate and visualize these probabilistic trajectories. This will equip clinicians with deeper, more actionable insights into clinical risk dynamics and potentially provide new information about causality in patient outcomes.

This study has limitations. ETHOS was demonstrated using PHTs derived from MIMIC-IV-ED data, and its direct applicability to data from other institutions may be limited without retraining using additional data from other institutions. Electronic medical record (EMR) systems vary significantly across institutions, influenced by differences in clinical practice, care pathways, patient populations, and geographic location. These variations can impede the direct transferability of AI models trained on one dataset to another. In certain applications, such as radiology or pathology, data inputs like medical images are relatively standardized, allowing models trained in one institution to perform well in others. However, EMR data pose unique challenges due to their variability. Models trained on data from one institution may produce inaccurate risk estimates when applied to data from another, particularly if clinical practices differ [30]. To mitigate this limitation, the model code for ETHOS-ARES is compatible with the MEDS [31] health AI data standard, making it easier for other researchers to reliably train identical model architectures on their local data.

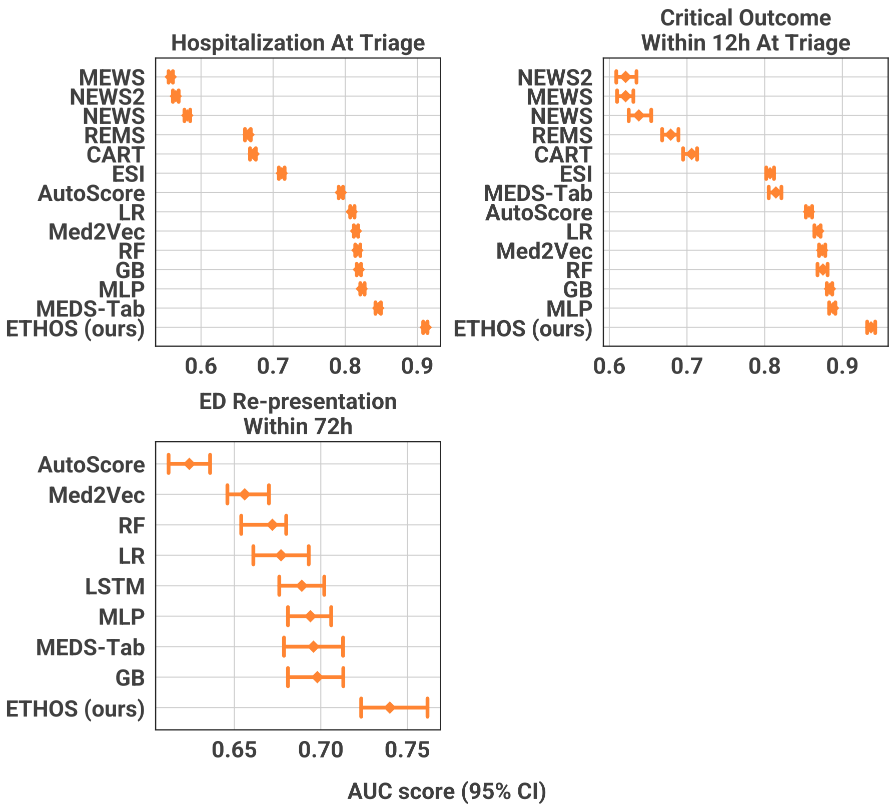

Figure 3. Predictive results for the ED benchmark tasks. Fewer methods appear in the ED re-presentation task (right) because score-based approaches, designed specifically to estimate in-hospital deterioration, are not applicable once the patient has left the ED. Ethos consistently achieves the best performance across all evaluated tasks.

Data standardization is often proposed as a solution to address the challenges of variability in healthcare data. However, achieving meaningful standardization would require identifying commonalities between healthcare systems, an endeavor that may not be feasible given the diversity of clinical practices, patient populations, and institutional workflows. In our view, a more robust approach is to train AI models, such as ETHOS, on raw data from diverse institutions, allowing the model itself to learn and interpret the underlying patterns and clinical pathways. This approach mirrors the capability of large language models (LLMs) to discern meaning from vastly different styles of text and presentations or even different languages, leveraging the same foundational transformer architecture as ETHOS.

In summary, artificial intelligence advancements have unlocked unprecedented opportunities for innovative solutions like ARES, which leverage large amounts of heterogeneous data to develop general-purpose models with superior predictive power compared to state-of-the-art methods. ARES not only enables dynamic, personalized risk estimation but also provides real-time explainability, empowering clinicians to make more informed decisions. Furthermore, its modular design and the underlying ETHOS model allow for seamless integration of additional data types, such as radiology, genetics, and other institutional datasets, paving the way for even greater predictive accuracy and applicability in diverse healthcare settings.

As healthcare costs and complexity continue to rise, PHT-based frameworks like ARES show a promising pathway towards data-driven AI-enabled individualized patient care with the potential to reduce morbidity, improve outcomes, and lower healthcare costs.

Potential implications

Authors should provide some additional comments about potential, more broad-ranging implications of their work that are not directly related to the current focus of their manuscript. This section is meant to promote discussion on possible ways the findings or data presented might be used in or have a relationship with other areas of research that may not be directly apparent in the work. It is not meant to provide 'proof of importance' of the work. Only to engender expansion of use to other areas.

Explicit personal opinions by the authors are permitted, but they should be made clear as such. References or related information to support the propositions should be included. These section should focus on work that can be done within the foreseeable future and specifically using the information within the manuscript, not provide speculation on how it will relate to far-reaching goals of the research area.

Methods

Data Preprocessing

We extracted relevant data from the MIMIC-IV tables as detailed in Table S7. Laboratory tests and medications were standardized using ATC codes, and all diagnostic and procedural codes were mapped to ICD-10 when necessary, as described in detail [14]. Additional tables requiring advanced processing, such as clinical notes, were not included in the current implementation of ETHOS.

Tokenization, PHT Construction, model training

The core of ETHOS lies in constructing PHTs from electronic medical records (EMRs) using a tokenization strategy that captures

diverse clinical events. A PHT represents a patient's medical history as a sequence of tokens, each encoding specific health-related information organized chronologically. This structured representation enables comprehensive modeling of patient journeys and more accurate clinical predictions. To build PHTs we used the MEDS-DEV [32] extraction pipeline that converts EHR data to an intermediate format called MEDS [31] to facilitate further data transformations. Advanced transformation operations were subsequently applied, breaking down each event into 1 to 7 tokens based on its complexity. Simpler events required fewer tokens, while intricate ones, such as multi-component lab results, were represented with more tokens to encapsulate their detailed information.

For example, lab test results were encoded using quantile-based tokens to represent clinical significance. Time-interval tokens were added to mark the elapsed time between successive events, with intervals shorter than 5 minutes omitted and longer gaps tokenized into 19 distinct interval tokens. Continuous numerical values, such as lab test results, were similarly quantile-encoded using ten quantiles, balancing clinical interpretability and predictive precision. Diagnostic and procedural codes, including ICD-10-CM, ICD-10-PCS, and ATC drug codes, were encoded hierarchically, which leveraged their inherent structure to enhance the transformer model's attention mechanisms. For more details, refer to [14].

Static patient attributes such as gender, marital status, race, and body mass index (BMI) were encoded using a single token depending on the value. For age, tokens of quantiles were reused, allowing age representation from 0 to 99. For instance, a 46-year-old patient would be coded as Q5 and Q7. Attributes with potential variability were represented using their most recently known value at the start of the timeline. By incorporating these elements, ETHOS ensured a richer and more adaptable representation of patient timelines.

The dataset was split into two disjoint groups: training/validation (90%) and testing (10%). During the training phase, 6 million tokens (1.8% of the entire dataset) were used for validation to balance model optimization and computational efficiency. The detailed statistics about the tokenized dataset are available in Table S6 and S9, and information about the model is in S1.

Probabilistic inference

The ETHOS model generates probabilities of future clinical events by leveraging tokenized PHTs and employing a transformer-based generative model. During inference, ETHOS autoregressively generates tokens, each representing a potential future event, until predefined stopping conditions are met, such as the appearance of a token of interest or meeting the simulation time limit. By simulating multiple future PHTs (fPHTs) for each patient, ETHOS accounts for inherent uncertainties and produces robust probabilistic predictions. For example, if N simulated fPHTs are generated and M indicates inpatient mortality, the estimated probability of mortality is calculated as M/N (Section). This stochastic, scenario-based methodology enables comprehensive modeling of patient trajectories, facilitating precise and dynamic risk assessments tailored to individual patient profiles.

Explainability

As illustrated in Figure 4, stochastic simulations can be initiated not only from the most recent token representing current information but also from any preceding token in the patient's history. This allows risk estimates to be visualized as a time series, highlighting how specific medical events affect risk over time. This approach provides intuitive visualizations, offering clinicians clear insights into the factors contributing to current risk values.

Methods used for benchmarking

We followed benchmarking tasks for emergency department models presented in the Emergency Department MIMIC-IV-ED benchmark paper [27]. Three tasks were defined: prediction of the hospital admission at triage, prediction of the critical outcome (death or transfer to ICU within 12 hours) at triage, and ED re-presentation within 72 hours after discharge from ED. We applied machine learning methods (logistic regression, random forest, gradient boosting), scoring systems MEWS [12], NEWS [11, 33, 34], Rapid Emergency Medicine Scores (REMS) [35], Cardiac Arrest Risk Triage (CART) [36], five-level triage system Emergency Severity Index (ESI) [37] and neural networks-based models including multilayer perceptron, Med2Vec [38] and Long Short-Term Memory (LSTM) [39].

To compare tasks used for early warning scores, we compared the MEDS-Tab library [40] which was used to establish a baseline. MEDS-Tab converts time-series EHR data into a tabular format by aggregating features across multiple time windows. It takes longitudinal patient data and applies various aggregation functions (like sum, count, min, max) over different historical window sizes to create fixed-size feature vectors, where each feature represents a combination of a medical code, time window, and aggregation method. XGBoost [41] models are trained on these tabular features computed from data windows prior to each prediction time point for each clinical task.

Statistical Methods

The performance of predictive models was evaluated using Receiver Operating Characteristic (ROC) curves and corresponding Area Under the Curve (AUC) values. Bootstrapping techniques were employed to estimate 95% confidence intervals (CIs) for AUCs. Model predicted probabilities were compared with observed event frequencies using calibration curves to evaluate ETHOS's reliability and alignment with real-world clinical outcomes. All statistical analyses were conducted using Python-based libraries, including scipy and scikit-learn [42, 43]. Data visualization, including ROC curves, calibration plots, and other statistical figures, was performed using matplotlib, seaborn and altair.

Availability of source code and requirements

- Project name: ETHOS-ARES
- Project home page: `github.com/ipolharvard/ethos-ares`
- Operating system(s): Platform independent
- Programming language: Python
- Other requirements: Polars etc
- License: MIT

Data availability

The MIMIC-IV dataset is publicly available at `https://physionet.org/content/mimiciv/2.2` `https://doi.org/10.13026/6mm1-ek67`. Its Emergency Department extension is available at `https://physionet.org/content/mimic-iv-ed/2.2/` `https://doi.org/10.13026/5ntk-km72`.

Declarations

List of abbreviations

- **AI** – Artificial Intelligence
- **ARES** – Adaptive Risk Estimation System
- **ATC** – Anatomical Therapeutic Chemical (codes)

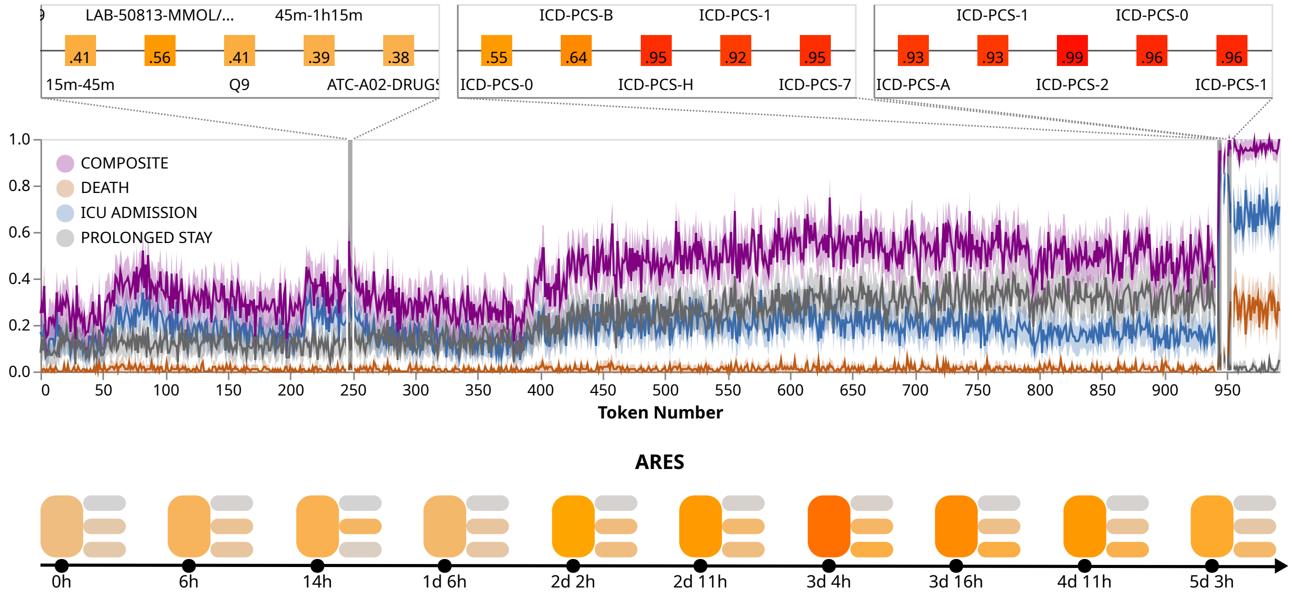

Figure 4. ARES Risk Trajectories. This figure illustrates risk trajectories for nearly 1000 tokens preceding patient death, as monitored by ARES, which evaluates the probability of death, ICU admission, prolonged hospital stay, and a composite risk score. The lower panel provides a color-coded representation of risk with the actual time since the ED presentation. In contrast, the upper panel highlights three 5-token regions influencing risk predictions at areas marked by the thin gray bar. In the first region, token LAB-50813 (Lactate Blood Test) increases the composite risk score from 0.41 to 0.56, but since the result falls in Q9 (80–90th percentile), ETHOS downgrades the risk estimate back to the previous level. In the second region (close to the end), a sharp increase in composite risk occurs due to heightened ICU admission triggered by ICD-PCS code 0BH17EZ, which is coded by 7 tokens (only 5 visible), which represents Endotracheal Airway Insertion into the Trachea via Natural or Artificial Opening. The 'H' token specifically signals ETHOS to escalate the ICU risk to nearly 1.0, indicating that the patient is being intubated de novo. The ICD-10-PCS breakdown confirms the procedure as a respiratory intervention involving tracheal insertion via a natural or artificial opening. ICD-PCS 0BH17EZ does not increase the risk of death, but the next ICD-PCS 5A12012 (5 tokens coding A1202 visible) raises the risk of death to about 0.25. We note that an increased risk of death is associated with a decreased risk of ICU admission, as these are competing risks. This visualization demonstrates how ARES dynamically adjusts risk scores based on evolving patient data, integrating clinical trajectories into real-time risk assessment.

- **AUC** – Area Under the Curve
- **BIDMC** – Beth Israel Deaconess Medical Center
- **BMI** – Body Mass Index
- **CART** – Cardiac Arrest Risk Triage
- **CI** – Confidence Interval
- **ED** – Emergency Department
- **EHR** – Electronic Health Record
- **EMR** – Electronic Medical Record
- **ESI** – Emergency Severity Index
- **ETHOS** – Enhanced Transformer for Health Outcome Simulation
- **fPHT** – Future Patient Health Timeline
- **GDP** – Gross Domestic Product
- **ICD-10** – International Classification of Diseases, 10th Revision
- **ICU** – Intensive Care Unit
- **LLM** – Large Language Model
- **LSTM** – Long Short-Term Memory
- **MEDS** – Medical Event Data Standard
- **MEWS** – Modified Early Warning Score
- **MIMIC-IV** – Medical Information Mart for Intensive Care, version IV
- **ML** – Machine Learning
- **NEWS** – National Early Warning Score
- **PHT** – Patient Health Timeline
- **REMS** – Rapid Emergency Medicine Score
- **ROC** – Receiver Operating Characteristic
- **XGBoost** – eXtreme Gradient Boosting

Consent for publication

Not applicable

Competing Interests

Dr. Jia is also affiliated with Verily Life Science. Dr. Cunningham reports consultancy for Edgewise Therapeutics, Occlutech, and us2.ai. Dr. Bates reports grants and personal fees from EarlySense, personal fees from CDI Negev, equity from ValeraHealth, equity from Clew, equity from MDClone, personal fees and equity from AESOP, personal fees and equity from FeelBetter, personal fees and equity from Guided Clinical Solutions, outside the submitted work. Other authors declare no competing interests.

Funding

Dr. Sitek reports grant support NIH R01HL159183, and Dr. Cunningham reports grant support from the American Heart Association (23CDA1052151) and the National Heart, Lung, and Blood Institute (1K23HL168163).

Author's Contributions

- **PR** – Conceptualization, Methodology, Formal Analysis(lead), Visualization, Software(lead), Writing—Review, and Editing.
- **MKG** – Conceptualization, Methodology, Formal Analysis, Writing—Review, Editing, and Visualization.
- **NO** – Formal Analysis.
- **DG** – Writing–Review, and Editing.
- **YJ** – Methodology, Writing–Review, and Editing.
- **SB** – Conceptualization.
- **MBAM** – Methodology, Writing–Review, and Editing.
- **JW** – Writing–Review, and Editing.
- **AES** – Writing–Review, and Editing.
- **JWC** – Writing–Review, and Editing.
- **DWB** – Writing–Review, and Editing.

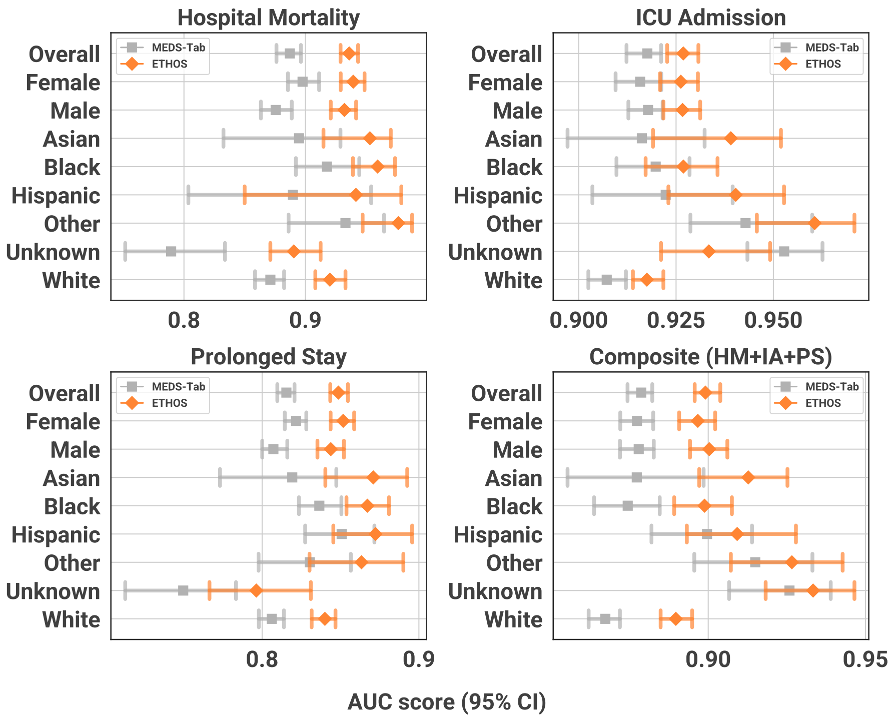

Figure 5. AUC Comparison Between ETHOS and MEDS-Tab Across Demographic Subgroups and Prediction Tasks. AUC scores with 95% confidence intervals are shown for ETHOS (orange) and MEDS-Tab (gray) across four prediction tasks: Hospital Mortality, ICU Admission, Prolonged Stay, and Composite Outcome (Hospital Mortality + ICU Admission + Prolonged Stay). Performance is reported for the overall population and stratified by gender (Female, Male) and race (Asian, Black, Hispanic, Other, Unknown, White). ETHOS consistently outperforms MEDS-Tab across all demographic subgroups and tasks.

- **AS** – Conceptualization(lead), Methodology, Writing–Original Draft, Preparation, and Supervision.

Acknowledgements

We thank Kinga Renc, M.Arch, for assisting with graphic design.

References

1. Gunja MZ, Gumas ED, Williams RD II, U.S. Health Care from a Global Perspective, 2022: Accelerating Spending, Worsening Outcomes;.
2. Committee on the Future of Emergency Care in the United States Health System . Hospital-based emergency care: at the breaking point. National Academies Press;.
3. Yang KK, Lam SSW, Low JMW, Ong MEH. Managing emergency department crowding through improved triaging and resource allocation;10:13–22.
4. Horton DJ, Graves KK, Kukhareva PV, Johnson SA, Cedillo M, Sanford M, et al. Modified early warning score-based clinical decision support: cost impact and clinical outcomes in sepsis;3:261–268.
5. Adams R, Henry KE, Sridharan A, Soleimani H, Zhan A, Rawat N, et al. Prospective, multi-site study of patient outcomes after implementation of the TREWS machine learning-based early warning system for sepsis;28:1455–1460.
6. Edelson DP, Churpek MM, Carey KA, Lin Z, Huang C, Siner JM, et al. Early warning scores with and without artificial intelligence;7:e2438986.
7. Gerry S, Bonnici T, Birks J, Kirtley S, Virdee PS, Watkinson PJ, et al. Early warning scores for detecting deterioration in adult hospital patients: systematic review and critical appraisal of methodology;369:m1501.
8. Winslow CJ, Edelson DP, Churpek MM, Taneja M, Shah NS, Datta A, et al. The impact of a machine learning early warning score on hospital mortality: A multicenter clinical intervention trial;50:1339–1347.
9. Escobar GJ, Liu VX, Schuler A, Lawson B, Greene JD, Kipnis P. Automated identification of adults at risk for in-hospital clinical deterioration;383:1951–1960.
10. Cummings BC, Blackmer JM, Motyka JR, Farzaneh N, Cao L, Bisco EL, et al. External validation and comparison of a general ward deterioration index between diversely different health systems;51:775–786.
11. Williams B. The National Early Warning Score: from concept to NHS implementation;22:499–505.
12. Subbe CP, Kruger M, Rutherford P, Gemmel L. Validation of a modified Early Warning Score in medical admissions;94:521–526.
13. Vaswani A, Shazeer N, Parmar N, Uszkoreit J, Jones L, Gomez AN, et al. Attention is all you need;30.
14. Renc P, Jia Y, Samir AE, Was J, Li Q, Bates DW, et al. Zero shot health trajectory prediction using transformer;7:256.
15. Yang Z, Mitra A, Liu W, Berlowitz D, Yu H. TransformEHR: transformer-based encoder-decoder generative model to enhance prediction of disease outcomes using electronic health records;14:7857.
16. Li Y, Mamouei M, Salimi-Khorshidi G, Rao S, Hassaine A, Canoy D, et al. Hi-BEHRT: Hierarchical Transformer-Based Model for Accurate Prediction of Clinical Events Using Multimodal Longitudinal Electronic Health Records;27:1106–1117.
17. Luo X, Rechardt A, Sun G, Nejad KK, Yáñez F, Yilmaz B, et al. Large language models surpass human experts in predicting neuroscience results;.
18. Thirunavukarasu AJ, Ting DSJ, Elangovan K, Gutierrez L, Tan TF, Ting DSW. Large language models in medicine;29:1930–1940.
19. Kraljevic Z, Bean D, Shek A, Bendayan R, Hemingway H, Yeung JA, et al. Foresight-a generative pretrained transformer for modelling of patient timelines using electronic health records:

a retrospective modelling study;6:e281–e290.

20. McDermott MBA, Nestor BA, Argaw P, Kohane I. Event Stream GPT: A data pre-processing and modeling library for generative, pre-trained transformers over continuous-time sequences of complex events;abs/2306.11547.
21. Steinberg E, Fries J, Xu Y, Shah N. MOTOR: A time-to-event foundation model for structured medical records;.
22. Li Y, Rao S, Solares JRA, Hassaine A, Ramakrishnan R, Canoy D, et al. BEHRT: Transformer for electronic health records;10:7155.
23. Jeong H, Oufattole N, Mcdermott M, Balagopalan A, Jangeesingh B, Ghassemi M, et al. Event-Based Contrastive Learning for medical time series;.
24. Johnson AEW, Bulgarelli L, Shen L, Gayles A, Shammout A, Horng S, et al. MIMIC-IV, a freely accessible electronic health record dataset;10:1.
25. Johnson A, Bulgarelli L, Pollard T, Horng S, Celi LA, Mark R. Mimic-iv;.
26. Goldberger AL, Amaral LA, Glass L, Hausdorff JM, Ivanov PC, Mark RG, et al. PhysioBank, PhysioToolkit, and PhysioNet: components of a new research resource for complex physiologic signals;101:E215–20.
27. Xie F, Zhou J, Lee JW, Tan M, Li S, Rajnthern LS, et al. Benchmarking emergency department prediction models with machine learning and public electronic health records;9:658.
28. Meng C, Trinh L, Xu N, Enouen J, Liu Y. Interpretability and fairness evaluation of deep learning models on MIMIC-IV dataset;12:7166.
29. Logan Ellis H, Palmer E, Teo JT, Whyte M, Rockwood K, Ibrahim Z. The early warning paradox;8:81.
30. Rajkomar A, Dean J, Kohane I. Machine learning in medicine;380:1347–1358.
31. Arnrich B, Choi E, Fries JA, McDermott MBA, Oh J, Pollard T, et al. Medical Event Data Standard (MEDS): Facilitating Machine Learning for Health. In: ICLR 2024 Workshop on Learning from Time Series For Health;. .
32. MEDS-DEV: Establishing Reproducibility and Comparability in Health AI;.
33. Smith GB, Prytherch DR, Meredith P, Schmidt PE, Featherstone PI. The ability of the National Early Warning Score (NEWS) to discriminate patients at risk of early cardiac arrest, unanticipated intensive care unit admission, and death;84:465–470.
34. Zhang S, Xu Y, Usuyama N, Xu H, Bagga J, Tinn R, et al. A multimodal biomedical foundation model trained from fifteen million image–text pairs;2.
35. Olsson T, Terent A, Lind L. Rapid Emergency Medicine score: a new prognostic tool for in-hospital mortality in nonsurgical emergency department patients;255:579–587.
36. Churpek MM, Yuen TC, Park SY, Meltzer DO, Hall JB, Edelson DP. Derivation of a cardiac arrest prediction model using ward vital signs;40:2102–2108.
37. Eitel DR, Travers DA, Rosenau AM, Gilboy N, Wuerz RC. The Emergency Severity Index triage algorithm version 2 is reliable and valid;10:1070–1080.
38. Choi E, Bahadori MT, Searles E, Coffey C, Thompson M, Bost J, et al. Multi-layer representation learning for medical concepts. In: Proceedings of the 22nd ACM SIGKDD International Conference on Knowledge Discovery and Data Mining ACM;. .
39. Hochreiter S, Schmidhuber J. Long short-term memory;9:1735–1780.
40. Oufattole N, Bergamaschi T, Kolo A, Jeong H, Gaggin H, Stultz CM, et al. MEDS-Tab: Automated tabularization and baseline methods for MEDS datasets;.
41. Chen T, Guestrin C. XGBoost: A Scalable Tree Boosting System. In: Proceedings of the 22nd ACM SIGKDD International Conference on Knowledge Discovery and Data Mining KDD '16, Association for Computing Machinery;. p. 785–794.
42. Virtanen P, Gommers R, Oliphant TE, Haberland M, Reddy T, Cournapeau D, et al. SciPy 1.0: fundamental algorithms for scientific computing in Python;17:261–272.
43. Pedregosa F, Varoquaux G, Gramfort A, Michel V, Thirion B, Grisel O, et al. Scikit-learn: Machine learning in python journal of machine learning research;12:2825–2830.

Click here to access/download
Supplementary Material
ETHOS_ARES___GigaScience_sup (1).pdf

Cover Letter
Click here to access/download;Personal Cover;Cover_letter_gigascience.docx.pdf

MASSACHUSETTS GENERAL HOSPITAL
HARVARD MEDICAL SCHOOL

Center for Advanced Medical Computing and Analysis
Massachusetts General Hospital, Harvard Medical School
100 Cambridge St., Boston, MA 02114 Suite 1303
asitek@@mgh.harvard.edu

Arkadiusz Sitek, PhD
Associate Investigator
Massachusetts General Hospital
Member of Faculty
Associate Professor pending
Harvard Medical School

Boston, March 2, 2025

Prof. Scott Edmunds, PhD
Editor-in-Chief
GigaScience Press,
BGI Hong Kong, Hong Kong

Dear Prof. Scott Edmunds,

I am pleased to submit our manuscript, **"Foundation Model of Electronic Medical Records for Adaptive Risk Estimation,"** for consideration in *GigaScience*. This work presents a novel transformer-based generative model for continuous, personalized, and explainable risk estimation in hospitalized patients. The model provides a unified solution for various clinical tasks without requiring task-specific retraining. The data used in this study is open-source and freely available. Additionally, we provide well-documented and professionally structured source code to facilitate straightforward reproduction of our results.

We believe this work will greatly interest your readership as it represents a major advance beyond current clinical prognostication concepts and methods. In current clinical risk assessment, research on pooled data from groups of patients defines average risk across groups of patients. Physicians identify a recognized group, apply the average clinical risk of an adverse outcome, and individualize patients as part of patient care. This is one of the reasons for the high value generally placed on physician experience. Our model is a learned representation of a vast number of patient trajectories. It can perform zero-shot (i.e., no additional training required) risk assessment for individual patients. The implications of individualized risk assessment are highly disruptive of the prevailing clinical care paradigm. Our Adaptive Risk Estimation System (ARES) shows a pathway to systems that can compute an early warning representing a specific probability of, for example, ICU admission for any patient regardless of their diagnosis or background information. ARES integrates all patient data and can continuously evaluate the likelihood of a wide range of clinically important outcomes. These outcomes can be redefined at any time without model retraining. ARES also provides patient-specific explainability, showing clinicians exactly which data elements drive each risk estimate.

Our model is trained on the publicly available MIMIC-IV database, which is a significant strength. MIMIC-IV's widespread use ensures that others can independently verify, extend, and reproduce our approach. We support this transparency by including thorough documentation and publicly accessible code.

We look forward to discussing how ARES can help shape the future of clinical risk prediction and decision support.

Thank you for your consideration.

Sincerely,

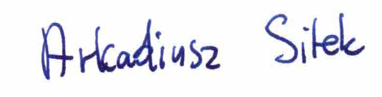

Arkadiusz Sitek, PhD
