# Supplementary material for: Foundation model of electronic medical records for adaptive risk estimation
Source: Gigascience. 2025 Sep 30;14:giaf107. doi: 10.1093/gigascience/giaf107 (PMC12482913; doi:10.1093/gigascience/giaf107)
Supplement: giaf107_GIGA-D-25-00088_Revision_1 [file giaf107_giga-d-25-00088_revision_1.pdf]

GigaScience

Foundation Model of Electronic Medical Records for Adaptive Risk Estimation

--Manuscript Draft--

**Manuscript Number:**	GIGA-D-25-00088R1
**Full Title:**	Foundation Model of Electronic Medical Records for Adaptive Risk Estimation
**Article Type:**	Research
**Funding Information:**	NHLBI Division of Intramural Research (R01HL159183) — Not applicable
**Abstract:**	Background, Hospitals struggle to predict critical outcomes. Traditional early warning systems, like NEWS and MEWS, rely on static variables and fixed thresholds, limiting their adaptability, accuracy, and personalization; Methods, We previously developed the Enhanced Transformer for Health Outcome Simulation (ETHOS), an AI model that tokenizes patient health timelines (PHTs) from EHRs and uses transformer-based architectures to predict future PHTs. ETHOS is a versatile framework for developing a wide range of applications. In this work, we develop the Adaptive Risk Estimation System (ARES) that leverages ETHOS to compute dynamic, personalized risk probabilities for clinician-defined critical events. ARES also features a personalized explainability module that highlights key clinical factors influencing risk estimates. We evaluated ARES using the MIMIC-IV v2.2 dataset together with its Emergency Department (ED) extension and benchmarked performance against both classical early warning systems and contemporary machine learning models.; Results, The entire dataset was tokenized resulting in 285,622 PHTs (63% with at least one hospital admission), comprising over 357 million tokens. ETHOS outperformed benchmark models in predicting hospital admissions, ICU admissions, and prolonged stays, achieving superior AUC scores. Its risk estimates were robust across demographic subgroups, with calibration curves confirming model reliability. The explainability module provided valuable insights into patient-specific risk factors; Conclusions, ARES, powered by ETHOS, advances predictive healthcare AI by delivering dynamic, real-time, personalized risk estimation with patient-specific explainability. Although our results are promising, the clinical impact remains uncertain. Demonstrating ARES's true utility in real-world settings will be the focus of our future work.
**Corresponding Author:**	Arkadiusz Sitek, Ph.D.<br>Massachusetts General Hospital<br>Sommerville, Massachusetts UNITED STATES
**Corresponding Author Secondary Information:**	
**Corresponding Author's Institution:**	Massachusetts General Hospital
**Corresponding Author's Secondary Institution:**	
**First Author:**	Pawel Renc
**First Author Secondary Information:**	
**Order of Authors:**	Pawel Renc
	Michal K Grzeszczyk
	Nassim Oufattole
	Deirdre Goode
	Yugang Jia
	Szymon Bieganski
	Matthew BA McDermott
	Jaroslaw Was
	Anthony E Samir

Powered by Editorial Manager® and ProduXion Manager® from Aries Systems Corporation

	David W Bates
	Jonathan W Cunningham
	Arkadiusz Sitek, Ph.D.
**Order of Authors Secondary Information:**	

Response to Reviewers:

AUTHORS: We gratefully acknowledge the reviewers for their time, thoughtful evaluations, and constructive critique of our work. We respectfully address each point raised below.

REVIEWER 1: Reviewer reports:
Reviewer #1: GIGASCIENCE REVIEW: Foundation Model of Electronic Medical Records for Adaptive Risk Estimation

This paper presents a new risk estimation system (ARES) capable of calculating real-time predictions throughout the patient journey in hospital thanks to an AI-based model using tokenized patient timelines (ETHOS). This new risk estimation system offers promising new possibilities and differs from other fixed time-points, which seem less flexible and agile.

On the general reading of the article, although the paper introducing the ETHOS framework is cited ([14]), it would be useful for the reader and the whole understanding of the article to provide more information about this framework in the introduction. As it stands, the reader is almost obliged to read the paper cited in [14] in order to understand the article.

AUTHORS: We appreciate this suggestion. We have expanded the first part of the Methods to include additional details on how ETHOS operates, and we have added a new section in the Supplementary Data that describes the model training and provides an intuitive explanation of ETHOS's workflow. We hope these revisions improve the clarity of the ETHOS framework, such that readers will only need to consult our original paper for in-depth technical questions.

REVIEWER 1: A few major questions and reviews arise from our reading of the article:

- Firstly, from the clinical point of view :
o It seems that the most useful alert is the one given at least 12 to 24 hours before the event. ARES provide warnings in real-time, how would it be possible to evaluate its ability to predict events 24 hours before they occur? If an alert is too close to the predicted event, preventive measures cannot be taken. On the other hand, a prediction too close to the event risks using information that already reflects the event itself. There is therefore a risk of over-matching or artificially improving model quality. For example, predicting ICU admission knowing that the patient has just been intubated and ventilated makes no sense. Can the authors explain this point?

AUTHORS: Thank you for this insightful comment. You are absolutely correct: an alert that occurs only moments before or after deterioration (e.g., immediately after intubation) has limited clinical utility. Our intention in Figure 4 was not to imply that ARES should alert clinicians based on post-intubation data, but rather to demonstrate that ARES faithfully tracks the patient's evolving severity, i.e. as soon as highly prognostic information (such as intubation) enters the record, ARES appropriately increases its risk estimate. Importantly, ARES' risk predictions are well calibrated, as demonstrated in our paper. We will revise the Figure 4 caption to emphasize that the increase in risks triggered immediately after invasive interventions, such as intubation, should be interpreted as severity markers rather than actionable warnings. We agree that prospective clinical validation, demonstrating that ARES alerts delivered with adequate lead time can improve decision-making and patient outcomes, is essential. This is one of the directions that we will pursue in the future. We have added a statement in the discussion and in the abstract that indicates that clinical utility will be investigated in future work.

Powered by Editorial Manager® and ProduXion Manager® from Aries Systems Corporation

REVIEWER 1: o What is the clinical justification of the composite indicator? This composite criterion combines highly heterogeneous events of varying importance (ie mortality and prolonged length of stay). We suggest deleting this criterion, which is clinically irrelevant and provides no major information on the method.

AUTHORS: Thank you for this suggestion. The composite indicator was introduced not so much as a new clinical endpoint although it was suggested by clinicians (authors on the paper), but rather as a means of illustrating ETHOS's flexibility in jointly modeling multiple, potentially correlated outcomes. We selected three distinct events, ICU admission, prolonged hospital stay, and in-hospital mortality, to span a range of severities and types of events (classification and regression types combined), but any other set of endpoints could be combined in exactly the same way. This was to show adaptability and flexibility of our approach. Importantly, ETHOS naturally captures the statistical dependencies between events that are tracked in the ARES composite score.
We revised the manuscript to make clear that the composite criterion is an illustrative example of our framework's capacity to compute joint probabilities over arbitrary clinical events. Users may define and interpret any combination of endpoints, composite or individual, according to their clinical needs. This clarification emphasizes that our goal is to demonstrate the underlying technology, rather than to propose this specific composite as a clinical decision-making tool. This is now made clear in the second paragraph of section "Evaluation".
With this explanation that is not made clear in the manuscript we hope the reviewer would reconsider.

REVIEWER 1: - With regard to methodology :
o It would be useful to detail a little more detail on the flowchart. In particular, what can explain the loss of over 10 thousands patients between the 299 721 patients of the MIMIC-IV database and the 285 622 PHTs after the tokenization process used in the study? Does it introduce a bias or risk of poorer generalisation of the method for future use?

AUTHORS: We did not apply any explicit patient filtering. The reduction in patient count stems from the tokenization process, which excludes patients with no tokenizable information. As shown in Table S6, patient timelines vary greatly in length, with some exceeding 200,000 tokens and others nearly empty. Patients whose records contain only rare lab tests, for example, may be excluded, as ETHOS tokenizer only the 200 most common lab results. Table S7 details the data types used. These exclusions primarily affect patients with sparse records and occur automatically during tokenization.

REVIEWER 1: - In terms of results,
o It seems important to clarify the new key advantages of ARES over ETHOS. For example, it is not clear whether the results on figure 5 are provided by ARES and ETHOS or only ETHOS as they are very close to those presented on the ETHOS publication cited in the references [14]

AUTHORS: ETHOS is a foundation model and ARES system to track risk build based on ETHOS. ETHOS is a framework on which many applications can be built, one example being ARES. Since the publication of [14], the framework has been enhanced in multiple respects (the updated code is provided); however, these refinements are primarily implementation-level, as the core capability of generating future PHTs remains unchanged.
In order to avoid confusion, we carefully went through the manuscript and made clear what are the relations between ETHOS and ARES. We have clearly stated this point in the revised Background.

REVIEWER 1: o On figure 4: the signal provided by ARES appears noisy due to the granularity of the tokens. What enables ARES to detect key breaks in the risk

estimation timeline? Has token granularity been discussed in the modelling process in order to provide less noisy timelines?

AUTHORS: Thank you for this excellent point. In Figure 4, the apparent noise in the ARES risk trajectories arises primarily from variability in the finite Monte Carlo simulations, not from token granularity or interval binning. Each risk estimate is computed as the fraction of N simulated future PHTs that contain the event of interest; with a finite N, this naturally introduces jitter. We explicitly depict this uncertainty with shaded 95% confidence bands around the mean trajectory in all figures. While increasing N would reduce Monte Carlo noise, it also increases computational cost proportionally. We have clarified these points in the revised figure caption and expanded the "ETHOS and Probabilistic Inference" section of the Methods to discuss the role of sampling variability in the risk trajectories. We added an explanation in the caption of Figure 4. We also added in section Methods "ETHOS and Probabilistic Inference" how those confidence intervals were computed.

REVIEWER 1: - Concerning the benchmarks provided to assess the results,
o It would be interesting to provide measures dedicated to imbalanced class classification (as it is the case) such as F1-score and balanced accuracy.

AUTHORS: This was also pointed out by other reviewers. That's why we added a new figure in the Supplement with AUPRC curves for all tasks discussed in the paper, similar to the AUROC figure. AUPRC is commonly considered more informative in the presence of class imbalance and provides additional insight into ETHOS performance.

REVIEWER 1: o Variable structure is very particular in the ETHOS framework due to tokenization. Would it be possible to provide more information about data pre-processing to run baselines like logistic regression with this particular data structure?

AUTHORS: We used separate preprocessing pipelines tailored to each baseline method. For models from the ED-Benchmark paper, we followed their original count-based preprocessing approach. For the MEDS-TAB baseline, we applied their more advanced pipeline, which is also count-based but incorporates multiple time windows to better capture temporal structure. The ETHOS tokenization pipeline is specific to the ETHOS framework and was not used for classical models. While it would be technically possible to reverse the tokenized sequences into token counts and use them with classical models such as logistic regression, this would effectively reproduce or underperform the existing count-based baselines. Therefore, we chose to use the original preprocessing strategies for each baseline to ensure a fair and representative comparison. We added a section in supplementary data with additional information about benchmark methods.

REVIEWER 1: o Concerning the benchmarks, it would be interesting to provide an indicator of the energy consumption to train each of the algorithms and add it in the balance sheet.

AUTHORS: We estimated the energy consumption of the ETHOS training based on the resource utilization. ETHOS, which consists of approximately 45 million parameters, was trained on 8 A100 GPUs over 46 hours, resulting in an estimated 368 GPU-hours and approximately 220 kWh of energy consumption. Estimating energy usage for classical methods such as linear regression or XGBoost is less straightforward, as these models train quickly, often on CPUs, and generate minimal resource usage. While their energy footprint is likely much lower, this comes at the cost of substantially reduced performance, as demonstrated in our benchmark results. To provide further context, ETHOS is highly efficient compared to large language models. For example, training GPT-3 (175B parameters) required approximately 835,000 GPU-hours and 1,287,000 kWh, while LLaMA 3 (8B) and Falcon (40B) consumed around 490,000 kWh and 307,000 kWh, respectively. This highlights that our approach strikes a favorable balance between performance and environmental impact.

Powered by Editorial Manager® and ProduXion Manager® from Aries Systems Corporation

REVIEWER 1: We would also like to point out a few other minor reviews:
- On figure 4 : the colours differ between the legend and the figure itself

AUTHORS: Fixed

REVIEWER 1: The references are incomplete: we cannot see the names of journals.

AUTHORS: We apologize for the oversight. We used the GigaScience LaTeX template and forgot to convert the format of our bibliography from BibLaTex to BibTex. We have fixed this issue in the revised version of the paper and now all references appear correctly.

REVIEWER 2: Reviewer #2: The authors present ETHOS, a novel deep learning method for dynamic, personalized, and explainable clinical outcome prediction based on EHR. The approach is relevant and, in my view, holds strong potential for actual implementation in clinical settings.

AUTHORS: We thank the reviewer for positive comments and are also excited about the potential of this approach.

REVIEWER 2:Question to authors:
- Why is ETHOS currently restricted to ED settings? Could it generalize to inpatient/outpatient contexts with broader training?

AUTHORS: Actually, we used the MIMIC-IV v2.2 dataset with the MIMIC-IV-ED extension. So the PHTs describe patient admissions to ED, followed by potential hospital admissions and ICU admissions etc. In other words, it is not only ED data. We now make this clear in the abstract and introduction.

REVIEWER 2:- In "Analyses," please add a brief explanation about what the remaining 40% of PHTs represent.

AUTHORS: The MIMIC-IV v2.2 dataset contains records for 299,721 patients, but only around 60 percent have at least one documented hospital admission. As noted in our response to Reviewer 1, after tokenizing the entire dataset, we retain 285,622 PHTs. This number is lower than the total number of patients because those with no tokenizable information (patients with very sparse records) are automatically excluded during tokenization. We have updated this number in the abstract and revised the 60 percent to 63 percent to more accurately reflect the statistic of patients with at least one hospital admission in their records.

REVIEWER 2:- It seems all tasks can be framed as binary classifications. Have you explored ETHOS for multiclass or multilabel outcomes (diagnosis/ procedure codes, multiple LOS thresholds etc.)? How would thresholding work for overlapping events? If not, you could add a brief speculation in the Supplementary.

AUTHORS: In this work, the main focus was on ARES, for which we report results of binary classification tasks, to compare with benchmark models. However, the composite score is an example of a 3-class classification with events that can happen concurrently. The advantage of ETHOS, which generates multiple future PHTs, is that we can define any task related to modeled data with no additional training or fine-tuning.
In our previous publication, where we introduced ETHOS, we demonstrated hospital length of stay and SOFA score prediction, both regression tasks, as well as DRG code classification which was about 700- class multi-class classification, with excellent performance, all done with no finetuning.
We now explicitly state this in the Background (second to last paragraph).

REVIEWER 2:- Can you explain your data filtering approach for making predictions at admission level? E.g. truncation based on admission timestamp?

AUTHORS: Our tokenized data consists of a single sequence of all PHTs concatenated and separated by the TIMELINE_END token. The starting point for inference depends on the specific downstream task. For example, in the task of predicting whether a patient will be admitted to the hospital upon arriving at the emergency department, each sample is formed by including all tokens from the patient's timeline up to and including the last token recorded during triage evaluation. We also include as many preceding tokens as possible, up to the model's context limit of 2048 tokens.

REVIEWER 2:- How are thresholds determined for highlighting risk-contributing events?

AUTHORS: We now explain this at the end of the second to last paragraph in the background. We used a simple conversion between the risk and ordinal 5 risk level for ease of interporation.

REVIEWER 2:- Was any usability testing or qualitative feedback from clinicians conducted to assess how predictions and explanations might be integrated into real workflows? It would be nice to see at least a speculation (Supplementary) of how the authors envision doctors using the system, e.g., 1 patient case.

AUTHORS: This is a crucial point: although we present powerful technology, its true value lies in real-world use and improved patient outcomes. Physicians, including ED specialists who are co-authors, contributed to discussions on feasibility and implementation. We have not yet conducted formal usability testing, but we are actively planning pilot studies to evaluate ARES in clinical settings. We believe that ARES's adaptability and ability to be customized for specific workflows will enhance its adoption. Before live testing, we will perform mock implementations that simulate clinical scenarios based on PHTs. These studies will use both the MIMIC dataset and our own data from Massachusetts General Hospital. We have added a paragraph in the Discussion to address these plans.

REVIEWER 2:- Would you consider integrating an LLM layer to perhaps summarize or explain risk-contributing tokens in natural language? For instance, using an LLM to return a phrase about a newly highlighted token, which understands the entire patient trajectory and that doctors can also query.

AUTHORS: Thank you for this excellent suggestion. We agree that an LLM layer could greatly enhance interpretability by translating risk-driving tokens into concise, natural-language explanations. We are actually working on this, developing an LLM-based module that ingests the simulated future fPHT and generates summaries of the most influential events for each patient trajectory.

REVIEWER 2:- How does explainability work if only one component from multi-component information is highlighted as important for a prediction?

AUTHORS: We actually compute risk for each component so we know which components trigger changes.
Figure 4 illustrates this using the seven-character ICD-10-PCS code 0BH17EZ, tokenized one character at a time. The initial token, "0", merely indicates a medical and surgical procedure, and the second token, "B", specifies the respiratory system, neither of which meaningfully alters ETHOS's risk estimate. It is only upon processing the third token, "H" (Insertion), corresponding to intubation, that the predicted risk jumps sharply. The remaining tokens, "1" (Trachea), "7" (Via Natural or Artificial Opening), "E" (Endotracheal Airway), and "Z" (No Qualifier), provide further procedural detail but exert little to no additional effect on the risk score beyond the insertion marker.

REVIEWER 2:- Consider adding AUPRC plots alongside AUC (Figure 5), given tasks are imbalanced.

AUTHORS: We have added a figure showing the AUPRC for all tasks in the Supplement.

REVIEWER 2:- Add a brief explanation, at least in the Supplementary, about how

baseline algorithms (e.g. Figure 3) work and differ from your method.

AUTHORS: We have added an entirely new section in the Supplement, titled Baseline Models, to provide more detail on the algorithms used for comparison with ETHOS.

REVIEWER 2:- The "Potential implications" section appears to be placeholder text, please revise.

AUTHORS: Thank you for pointing out this oversight. We have replaced the placeholder with a fully developed “Potential implications” section in the revised manuscript.

REVIEWER 2:- How do you potentially see the integration of clinical notes in the system?

AUTHORS: We’ve begun preliminary (unpublished) work to embed clinical notes directly into the patient-health timeline (PHT). In our current approach, we convert each note into one or more fixed-size vectors, using a model such as BioMed-CLIP, and insert them at the appropriate time points in the PHT, treating them as embeddings of tokens representing clinical notes. During training, these embeddings are held constant, and although we have not yet observed clear performance gains, our investigation is ongoing and we will continue refining the integration strategy. In parallel, we plan to incorporate information from radiology reports and imaging studies, both as text embeddings and, ultimately, as image-derived features, into the same framework. We added a paragraph in the discussion.

REVIEWER 2:- How does ETHOS handle variability in patient history length (e.g. patients with extensive vs. minimal prior data)? Has performance been evaluated across short and long PHTs?

AUTHORS: This is an excellent point. In our current implementation, ETHOS uses a fixed context window of 2 048 tokens, so any patient history beyond that length is truncated and not visible to the model. While this is a limitation of our chosen configuration, not of the underlying method, the window size can be increased in the same way it is for large language models, albeit at the cost of substantially larger model parameters and greater data requirements. It is our future work.
During training, we concatenate all PHTs into a single sequence (350+ million tokens). We then follow the standard transformer training procedure: in each training step, we randomly sample a contiguous 2048-token chunk and train the model to predict the next tokens. As a result, some chunks may span the boundary between two patients’ timelines, but because we insert a dedicated TIMELINE_END token at the end of each PHT that informs the model that two or more patient data is present in the context.
At inference time, we handle PHT length uniformly by left-padding shorter PHTs up to 2048 tokens, while for longer PHTs we feed in only the most recent 2 048 tokens as context. This ensures consistent behavior regardless of the original timeline length.

REVIEWER 2:- The token distribution shows extreme variation in both frequency and diversity across code groups. Could the authors elaborate on how the system handles this? Was synthetic data or oversampling considered to improve the representation of these less frequent events?

AUTHORS: We have not yet examined how prediction accuracy varies with token frequency. This is an excellent point, and we plan to investigate it. One important application of ETHOS that we plan to look into is in diagnosing rare diseases, where the corresponding tokens are inherently infrequent. In such cases, ETHOS may struggle to model probabilities of occurrence of those tokens in fPHTs accurately. Should this limitation arise, we will need to develop methods to debias rare-event estimates. However, preserving overall calibration is challenging, approaches like oversampling or synthetic data augmentation for rare tokens could distort calibration for more common events.

REVIEWER 2:Minor revisions:

	- Add a reference to support the claim in "The United States allocates nearly 18 percent of its GDP to healthcare, yet….".<br><br>AUTHORS: Done<br><br>REVIEWER 2:- Add references to support the claim in "Similarly, conventional machine learning models depend on preselected predictors…".<br><br>AUTHORS: We removed this sentence in the revised manuscript.<br><br>REVIEWER 2:- Provide the class balance for the four prediction tasks in the main paper.<br><br>AUTHORS: We have added information about it in the second paragraph of the Evaluation section. Also, it is worth to note that this information is also present in Table S1 and Figure S2.<br><br>REVIEWER 2:- Add a figure, at least in the Supplementary, to exemplify patient data before/after tokenization. Even reusing the example provided in the MEDS paper could help clarify the technique.<br><br>AUTHORS: We have added a table in the Supplement with a short sample of original data (MIMIC-IV-DEMO), and the tokenized timeline used in ETHOS.<br><br>REVIEWER 2:- Add a brief explanation of the (HM+IA+PS) composite risk score in the "Analyses" section.<br><br>AUTHORS: Done<br><br>REVIEWER 2:- In the "Probabilistic inference" section, fix (Section) in "…the estimated probability of mortality is calculated as M/N (Section)."<br><br>AUTHORS: Done<br><br><br>REVIEWER 3: Reviewer #3: This manuscript introduces the Enhanced Transformer for Health Outcome Simulation (ETHOS) and the Adaptive Risk Estimation System (ARES), representing a novel approach to predicting critical healthcare outcomes using electronic health records (EHRs). The authors present a transformer-based foundation model that tokenizes patient health timelines (PHTs) to forecast future clinical trajectories and compute personalized risk probabilities for key outcomes such as mortality, ICU admission, and prolonged hospital stays. Notably, the system features an explainability module that highlights the clinical factors most influential in patient-specific risk assessments. The proposed approach is evaluated on the MIMIC-IV dataset, where it demonstrates superior predictive performance compared to traditional early warning systems and machine learning models.<br><br>However, I have several suggestions for the authors:<br><br>AUTHORS: Thank you, we address the suggestions below.<br><br>REVIEWER 3:1. Bias and Generalizability: While the discussion of limitations is thoughtful, it would benefit from a more direct examination of potential biases present in the training data—such as demographic or institutional biases—and how these might affect the model's generalizability to diverse patient populations or external datasets.<br><br>AUTHORS: We performed subgroup analyses in Figure 5 to demonstrate that ETHOS-derived metrics consistently outperform MEDS across key demographic groups; these evaluations were intended to illustrate relative performance rather than to serve as a comprehensive bias audit. We fully agree that a more rigorous investigation of potential demographic and institutional biases is important, and we have now added a clear statement to the revised manuscript’s limitations section to reflect this.

Powered by Editorial Manager® and ProduXion Manager® from Aries Systems Corporation

REVIEWER 3:2. Additional Limitations: Although the limitations of training on a single dataset are acknowledged, other important limitations are not fully explored. In particular, issues related to data missingness, temporal biases inherent in EHR data, and the potential for model drift over time should be discussed in greater detail.

AUTHORS: We agree that these are important considerations. Although MIMIC-IV is a well-curated dataset with relatively clean data and low levels of missingness, real-world EHRs often contain far more irregularities. ETHOS is designed to accommodate incomplete timelines by generating risk estimates based on whatever data are available at each time point. However, we acknowledge that higher degrees of missingness can still degrade model performance. ETHOS also mitigates certain temporal biases by always generating predictions based on the most recent available context, helping ensure that forecasts are contemporaneous with current patient status. While we have not yet conducted a thorough investigation into these effects, we recognize their importance. In our revised manuscript, we have expanded the Limitations section to discuss how data missingness, temporal bias inherent in EHRs, and potential model drift may affect ETHOS's performance in real-world deployment. We plan to address these issues in future validation and recalibration studies.

REVIEWER 3:3. Manuscript Formatting:The overall format of the manuscript could be further improved for readability and professionalism. For example, the abstract should be justify-aligned to enhance visual presentation and consistency with journal standards.

AUTHORS: Thank you for spotting that mistake. We inspected the manuscript and corrected the bibliography format used by us so it conforms to the GigaScience template. Now, journal names and years of publications appear correctly in the references.

REVIEWER 4: Reviewer #4: This manuscript presents a transformer-based model—Enhanced Transformer for Health Outcome Simulation—and its application in adaptive risk estimation using EHR data. The model is evaluated on MIMIC-IV and benchmarked against a set of traditional and machine learning approaches. The authors position this work as a zero-shot foundation model for real-time, personalized clinical risk prediction.
While the manuscript introduces a potentially impactful approach to clinical AI, several substantive concerns must be addressed before the work can be considered for publication. These primarily relate to methodological clarity, evaluation rigor, and support for the model's positioning as a "foundation model." The current version lacks sufficient technical and empirical depth to support the central claims. Specific concerns include:

AUTHORS: We thank the reviewer for his comments and appreciate them much. We address the specific points raised by the reviewer below.

REVIEWER 4:1. Insufficient methodological detail: The manuscript omits key information about the model's training procedure and learning objective(s). It is unclear whether masked token prediction, autoregressive modeling, or another strategy was used. Details on the loss functions, training epochs, optimization strategy, and data preprocessing are essential to assess reproducibility and scalability.

AUTHORS: To develop ARES, we leveraged our previously introduced ETHOS framework (foundational model), and the training procedure closely follows that of our prior publication. The training procedure is also relatively standard for any GPT model. We agree with the reviewer that the current paper would benefit with more extensive information about the original Framework and how it was trained. To this effect, in the Appendix of revised paper, we provide a comprehensive model description, including hyperparameter settings and training protocols. ARES is implemented as an autoregressive GPT-style transformer trained with the standard multiclass cross-entropy objective. We also added in the appendix an intuitive explanation for the ETHOS work. In addition to this, we also extended Methods/ETHOS and Probabilistic Inference sections to provide basic information about ETHOS and direct readers to

appendix for more information, and our previous work for comprehensive coverage. We also provide an entire code base for training ETHOS and implementation of ARES.

REVIEWER 4:2. Model and dataset scale: The model is trained on EHRs from approximately 300K patients, which is modest relative to other models such as MedBERT (trained on over 28 million patients). Similarly, the model's parameter count is relatively small. It is not evident that either the data scale or model capacity meets the bar typically associated with foundation models.

AUTHORS: Our model is actually three times bigger than Med-BERT's in terms of number of parameters (45M vs 17M). The reason for this is that 28M patients are used but each patient contributes an average 15 codes (tokens), so their training set has about 420M tokens. By contrast, our 300K patients generate richly annotated long timelines that can be of length of 200,000 tokens and include diagnoses, labs, vitals, medications, procedures, and time intervals, that collectively amount to a similar number (357M) of tokens vs. MedBERT that handles ICD code only.
Authors of MedBERT use large vocab sizes to encode ICD codes using one to one correspondence. We also use all ICD codes, however we address the large number of possible codes by using hierarchical tokenization which dramatically reduces the number of required token types. Our justification for choosing vocab size, context size, and model size vs. the number of tokens in the training set was established ad hoc in trial and error as well as guided from text processing domain and sizes of GPT-2 model which is trained on similar size of training data. That was investigated in our previous publication about ETHOS.
Our model is scalable and learns to predict future health trajectories directly from complex timelines, without downstream fine-tuning, demonstrating true zero-shot generalization across tasks. We believe this ability to ingest diverse, high-resolution EHR streams and immediately generate actionable risk predictions is the hallmark of a foundation model as is currently understood in the field.

REVIEWER 4:3. Benchmarking omissions: The comparative evaluation lacks transformer-based baselines trained on clinical data, such as MedBERT. Including such models would provide a more meaningful benchmark than comparisons with classical ML methods alone.

AUTHORS: Thank you for the suggestion. At present, no publicly available transformer-based model has been pretrained for real-time early-warning on clinical time-series data. Existing "clinical BERT" variants such as Med-BERT and ClinicalBERT are designed for discrete, visit-level disease prediction based on ICD codes and are not readily applicable to continuous streaming risk scoring investigated here. Adapting these models would require redesigning the tokenization schema to encode fine-grained vital-sign and laboratory trajectories, developing a low-latency inference pipeline capable of scoring each new measurement as it arrives, and retraining or further pretraining with a next-alert or masking objective specifically tailored to streaming data.
Given the absence of an off-the-shelf Transformer baseline and the considerable engineering effort required, we chose to benchmark ARES against well-established classical and recurrent early-warning models whose input formats and training regimes closely mirror our use case. Using pretrained Transformer embeddings into a streaming risk-prediction framework is a possible avenue for future work, but far from obvious for us how to implement this, and deserves separate investigation.

REVIEWER 4:4. Exclusion of unstructured clinical text: The decision to exclude clinical notes—a rich source of patient-level information—is a major limitation. The authors should discuss how this exclusion impacts performance and generalizability, particularly for a model intended to operate in real-world, zero-shot settings.

AUTHORS: Thank you for highlighting this important limitation. Our current implementation of ETHOS relies exclusively on structured EHR data, encoded as tokenized events, because incorporating free-text notes into the Patient Health

	Timeline (PHT) is not straightforward. Clinical notes often contain complementary insights (e.g., nuanced physician impressions, social factors) that are not fully captured by tabular fields, and their inclusion could improve ARES's predictive performance and generalizability in real-world, zero-shot settings.<br>However, integrating unstructured text presents several challenges: clinical narratives vary greatly in length and style, and it is unclear how best to segment, tokenize, and embed these notes alongside existing event tokens without overwhelming the model's capacity or diluting structured signals. We are actively exploring approaches, such as leveraging pretrained clinical-language-model embeddings, hierarchical note-chunk tokenization, and multimodal fusion techniques, and we will report on these efforts in future work. We also explain our current efforts in response to Reviewer #2. In the revised manuscript, we have added a discussion under "Limitations" that acknowledges the exclusion of clinical notes, considers its impact on model performance, and outlines our roadmap for multimodal PHT representations.<br><br>REVIEWER 4: 5. Evaluation methodology: The manuscript does not clarify whether all models were trained and evaluated on the same data splits, nor whether hyperparameter tuning or statistical testing was applied. This lack of rigor makes it difficult to interpret the significance of performance differences.<br><br>AUTHORS: We apologize for the lack of clarity. All models, ours and the benchmarks, were trained and evaluated on a single, fixed train/test split. This is now clearly stated in the "Data Preprocessing" section. Benchmark methods used the optimized settings and code provided by Xie et al. (2022). To quantify uncertainty, we computed 95 % confidence intervals for every metric using bootstrapped resampling; the non-overlapping intervals between ETHOS and competing methods demonstrate that our performance gains are statistically robust. We have now made these details prominent in the revised Methods and Results sections.<br><br>REVIEWER 4: 6. Zero-shot and "foundation model" claims: The manuscript emphasizes zero-shot capabilities but does not clearly contrast this with standard fine-tuning requirements in comparable models. Furthermore, the term "foundation model" is used without sufficient justification. Articulating the criteria used and how this model meets them (e.g., scale, generalizability, multi-task adaptability) would strengthen the framing.<br><br>AUTHORS: In medical AI, the term "foundation model" generally refers to a deep learning system that is (1) pretrained on very large datasets, often in an unsupervised or self-supervised fashion, (2) captures broad, high-dimensional representations that transfer across tasks, and (3) supports multi-task adaptation with minimal or no fine-tuning. ETHOS fulfills these criteria in the EHR domain. We trained it autoregressively on over 320 million tokens drawn from 257 082 patient health timelines, learning a rich representation of temporal clinical data without any task-specific labels. Once pretrained, ETHOS can perform a wide range of downstream predictions, mortality, ICU admission, prolonged stay, or composite endpoints, directly in a zero-shot manner by simulating future timelines and computing event probabilities, without any fine-tuning. This capability contrasts with standard transformer models that require separate fine-tuning for each new task. By virtue of its scale, generalizability across diverse endpoints, and zero-shot multi-task adaptability, ETHOS meets the established definition of a foundation model for EHR data. We have clarified this framing in the revised Background in the second paragraph. .<br><br>REVIEWER 4: In summary, the manuscript addresses an important and timely topic, but significant revisions are needed to clarify technical details, expand the evaluation, and substantiate key claims. At this stage, I do not recommend acceptance, but I encourage the authors to address these concerns in a future submission.<br><br>AUTHORS: Thank you for your comments. We appreciate your recognition of the importance and timeliness of our work. In response to your feedback, we have substantially revised the manuscript as described above. We believe these enhancements address the concerns raised and bring the manuscript to the level of rigor expected.

Powered by Editorial Manager® and ProduXion Manager® from Aries Systems Corporation

Additional Information:	
Question	Response
Are you submitting this manuscript to a special series or article collection?	No
**Experimental design and statistics**<br><br>Full details of the experimental design and statistical methods used should be given in the Methods section, as detailed in our Minimum Standards Reporting Checklist. Information essential to interpreting the data presented should be made available in the figure legends.<br><br>Have you included all the information requested in your manuscript?	Yes
**Resources**<br><br>A description of all resources used, including antibodies, cell lines, animals and software tools, with enough information to allow them to be uniquely identified, should be included in the Methods section. Authors are strongly encouraged to cite Research Resource Identifiers (RRIDs) for antibodies, model organisms and tools, where possible.<br><br>Have you included the information requested as detailed in our Minimum Standards Reporting Checklist?	Yes
**Availability of data and materials**<br><br>All datasets and code on which the conclusions of the paper rely must be either included in your submission or deposited in publicly available repositories (where available and ethically appropriate), referencing such data using a unique identifier in the references and in the "Availability of Data and Materials" section of your manuscript.	Yes

Powered by Editorial Manager® and ProduXion Manager® from Aries Systems Corporation

Have you have met the above requirement as detailed in our Minimum Standards Reporting Checklist?	
GigaScience has policies and guidelines in place for the use of generative AI-writing tools such as ChatGPT. If you have used such writing tools to assist with writing the manuscript this must be declared and cited in the text. Authors should not list AI-writing tools and other AI-assisted technologies as an author or co-author and should acknowledge that they are fully responsible for text generated or refined by AI-writing tools.<p><br><br>A summary of use (particularly in the introduction or among methods) needs to be included at the end of the paper, and the outputs should also be included as a supplementary file hosted in GigaDB or other open repositories. Please read our guidelines for more information. <p><br><br>By submitting to GigaScience, you are aware of the journal's AI-writing tools policy, and if you have declared use of such tools below, you have acknowledged this where appropriate in your manuscript and have made a summary of use and outputs available. <p><br><b>AI-assisted writing tools have been used in the preparation of this manuscript?	No

Powered by Editorial Manager® and ProduXion Manager® from Aries Systems Corporation

Manuscript Revised
Click here to access/download;Manuscript;ETHOS_ARES___GigaScience_

Placeholder for OUP logo oup.pdf

GigaScience, 2023, 1–13

doi: xx.xxxx/xxxx
Manuscript in Preparation
Paper

PAPER

Foundation Model of Electronic Medical Records for Adaptive Risk Estimation

Pawel Renc[1,2,3], Michal K. Grzeszczyk[1,2], Nassim Oufattole[4], Deirdre Goode[8,2], Yugang Jia[4], Szymon Bieganski[6], Matthew B. A. McDermott[2], Jaroslaw Was[3], Anthony E. Samir[1,2], Jonathan W. Cunningham[5,2], David W. Bates[5,7,2] and Arkadiusz Sitek[1,2,*]

[1]Massachusetts General Hospital, Boston, USA and [2]Harvard Medical School, Boston, USA and [3]AGH University of Krakow, Krakow, Poland and [4]Massachusetts Institute of Technology, Cambridge, USA and [5]Brigham and Women's Hospital, Boston, USA and [6]Medical University of Lodz, Lodz, Poland and [7]Harvard Chan School of Public Health, Boston, USA and [8]Newton Wellesley Hospital, Newton, USA

[*] Corresponding author: Arkadiusz Sitek, sarkadiu@gmail.com

Abstract

Background, Hospitals struggle to predict critical outcomes. Traditional early warning systems, like NEWS and MEWS, rely on static variables and fixed thresholds, limiting their adaptability, accuracy, and personalization; **Methods,** We previously developed the Enhanced Transformer for Health Outcome Simulation (ETHOS), an AI model that tokenizes patient health timelines (PHTs) from EHRs and uses transformer-based architectures to predict future PHTs. ETHOS is a versatile framework for developing a wide range of applications. In this work, we develop the Adaptive Risk Estimation System (ARES) that leverages ETHOS to compute dynamic, personalized risk probabilities for clinician-defined critical events. ARES also features a personalized explainability module that highlights key clinical factors influencing risk estimates. We evaluated ARES using the MIMIC-IV v2.2 dataset together with its Emergency Department (ED) extension and benchmarked performance against both classical early warning systems and contemporary machine learning models.; **Results,** The entire dataset was tokenized resulting in 285,622 PHTs (63% with at least one hospital admission), comprising over 357 million tokens. ETHOS outperformed benchmark models in predicting hospital admissions, ICU admissions, and prolonged stays, achieving superior AUC scores. Its risk estimates were robust across demographic subgroups, with calibration curves confirming model reliability. The explainability module provided valuable insights into patient-specific risk factors; **Conclusions,** ARES, powered by ETHOS, advances predictive healthcare AI by delivering dynamic, real-time, personalized risk estimation with patient-specific explainability. Although our results are promising, the clinical impact remains uncertain. Demonstrating ARES's true utility in real-world settings will be the focus of our future work.

Key words: Early Warning Scores; EHR; Foundation Model; Transformer; Zero-Shot Inference; Patient Health Trajectories

Background

The United States allocates nearly 18 percent of its GDP to healthcare [1], yet Americans have shorter lifespans and poorer health than residents of other high-income nations. Among these countries, the U.S. not only has the lowest life expectancy but also the highest rates of preventable deaths [2]. Hospitals face mounting challenges managing patient influx and identifying individuals at risk for critical outcomes, including mortality, intensive care unit (ICU) admission, or prolonged hospital stays [3]. Accurate prediction of critical clinical events is essential for enhancing patient care and optimizing the timely allocation of limited healthcare resources [4]. Early identification of at-risk patients

Compiled on: May 8, 2025.
Draft manuscript prepared by the author.

Key Points

- ARES enables dynamic, real-time risk estimation by predicting patient health timelines (PHTs) from electronic health records using a transformer-based model.
- ARES enhances clinical decision-making by leveraging ETHOS-generated future PHTs to provide personalized risk predictions with explainability.
- Methods used outperform traditional models in predicting critical outcomes while demonstrating strong calibration and equitable performance across demographic subgroups.

enables clinicians to prioritize interventions, anticipate potential escalations in care, and improve outcomes while simultaneously reducing costs [5, 6]. However, current methodologies often fail to fully utilize the vast and complex data available in electronic health records (EHRs), a limitation that becomes particularly evident in emergency settings where time-sensitive decisions are critical [7, 8, 9, 10, 11]. Traditional scoring systems, such as the National Early Warning Score (NEWS) [12] and the Modified Early Warning Score (MEWS) [13], rely on static variables and predefined thresholds, constraining their ability to adapt to dynamic and multifaceted patient data. These approaches are further hindered by their reliance on specific cutoff points for data inclusion (e.g., triage, 24-hour windows), which can overlook valuable longitudinal patterns.

Recent advances in generative machine learning—particularly transformer architectures [14, 15, 16, 17] that underpin Large Language Models [18, 19]—have unlocked unprecedented capabilities for processing high-dimensional, heterogeneous, time-stamped health data from EHRs [20, 21, 22, 23, 24]. In this work, we build on our Enhanced Transformer for Health Outcome Simulation (ETHOS) [15], which differs from prior efforts in its tokenization and handling of EHR events. ETHOS is autoregressively pretrained—without any task-specific labels—on over 321 million tokens drawn from 269,741 Patient Health Timelines (PHTs), learning broad, high-dimensional representations that transfer across tasks. Operating on PHTs (tokenized sequences of demographics, diagnoses, medications, etc.; see Table 7), ETHOS generates plausible future timelines (Figure 1) and delivers zero-shot predictions for mortality, ICU admission, prolonged stay, and composite endpoints without any additional fine-tuning. By virtue of its scale, generalizability, and multi-task adaptability, ETHOS serves as a *foundation model* for PHT generation.

Once trained, ETHOS can generate multiple simulated future patient health timelines (PHTs) and estimate the probability of clinical events occurring within those trajectories (e.g., ICU admission). For adverse events during an inpatient stay, these probabilities serve as dynamic risk estimates, effectively functioning as an early warning system. Unlike traditional methods that require separate models or task-specific retraining, ETHOS operates as a unified model capable of concurrently assessing multiple clinical endpoints. As new patient data become available, risk estimates are automatically updated. This flexible and scalable risk prediction framework, built on ETHOS, is referred to as the Adaptive Risk Estimation System (ARES), as illustrated in Figure 2. Risk is quantified into five ordinal categories (levels 1 through 5) based on the predicted probability: 0–20% corresponds to level 1, 20–40% to level 2, and so on.

In this paper, we present ARES and introduce a novel explainability framework that delivers fully personalized insights, potentially allowing clinicians to understand the specific factors influencing the system's risk predictions for individual patients. We benchmark the performance of ARES against state-of-the-art methods across multiple clinically relevant tasks, demonstrating its superior predictive accuracy. We validate its effectiveness and provide the accompanying code for the full reproduction of all the experiments by other researchers.

Data Description

In this study, we used the Medical Information Mart for Intensive Care (MIMIC-IV) version 2.2 database [25, 26], including its ED extension. MIMIC-IV, developed by the Massachusetts Institute of Technology and Beth Israel Deaconess Medical Center contains de-identified health records for almost 300,000 patients either admitted to the ED and/or hospital at BIDMC from 2008 to 2019. Detailed patient demographics are presented in Table 2.

Evaluation

Following the tokenization process, the data of 299,721 unique patients from the MIMIC-IV dataset was converted into 285,622 PHTs, which were subsequently used for training and testing. The discrepancy arises from some patients lacking associated data after tokenization. Of the total PHTs, approximately 63% (180,733) contained hospital admissions records. The tokenized dataset comprised over 357 million tokens in total. In the Supplementary Materials, we provide detailed information regarding the MIMIC-IV data used (Table 7), patient demographics (Table 2), characteristics of the PHTs (Table 6) and tokens (Table 11). The model was trained and validated on 90% of the PHTs, with the remaining 10% reserved for testing. During inference, at least $N = 100$ fPHTs were generated for each investigated task.

The predictive performance of ARES and MEDS-Tab was evaluated on three individual clinical endpoints—hospital mortality (HM), ICU admission (IA), and prolonged hospital stay (PS; defined as length of stay >90th percentile)—and, as an illustrative demonstration of joint risk modeling, on a composite criterion combining these events (HM-IA-PS). The prevalence of these tasks is: 1.85%, 15.44%, 9.01% and 20.39%, respectively. This composite endpoint demonstrates ARES's capacity to compute joint probabilities across heterogeneous outcomes and to naturally model their statistical dependencies. The composite score represents the cumulative risk of clinician-defined critical events. All predictions were generated at the hospital admission. As summarized in Figure 3, Figure 5, and Table 1, ARES consistently outperformed MEDS-Tab across both individual and composite endpoints, achieving higher AUC values in every case. Notably, these gains were observed across all racial subgroups, with the most pronounced improvements for Asian and Hispanic patients, indicating ARES's robustness and its potential to reduce disparities in predictive accuracy.

Figure 4 illustrates the dynamic risk trajectories generated by ARES, showcasing how the system continuously updates probability estimates for key clinical outcomes, including ICU admission, prolonged hospital stay, and mortality, as new clinical events occur. The figure highlights specific medical interventions, such as laboratory tests and procedures, that drive significant changes in risk estimates, demonstrating ARES's ability to integrate evolving patient data into real-time risk assessment. The results underscore the model's capacity to capture complex temporal relationships between clinical events, dynamically recalibrating risk scores based on patient status and treatment progression.

In addition to risk which are part of ARES and to contextualize

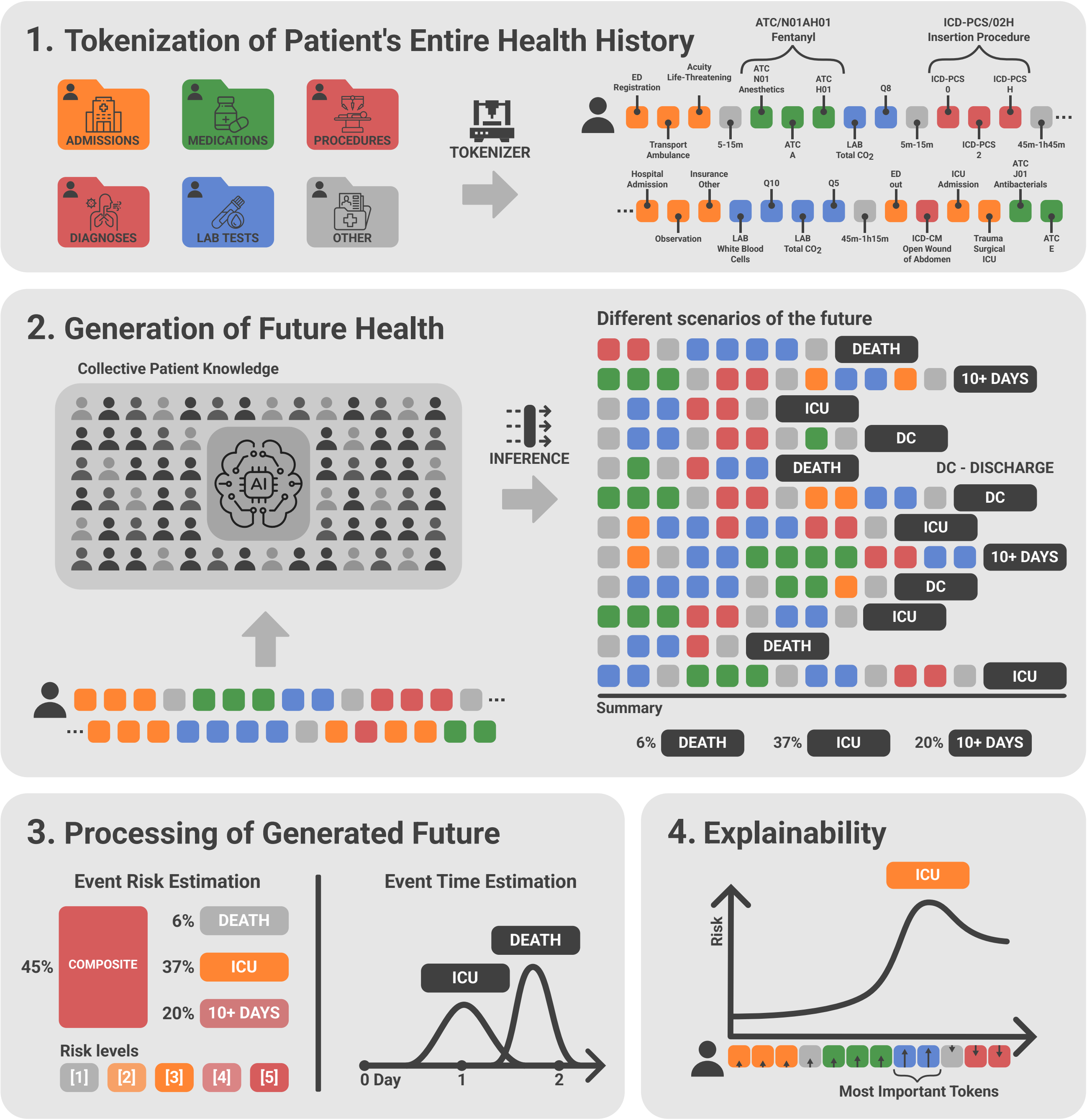

Figure 1. Workflow of the Adaptable Risk Estimation Score (ARES) Framework. This figure illustrates the ARES framework, developed on the ETHOS model, for dynamic and explainable risk evaluation. Panel 1 depicts the tokenization of a patient's entire health history into structured events represented as a sequence of tokens (PHTs), incorporating standardized coding systems such as ATC for medications, ICD-PCS for procedures, and others. Panel 2 demonstrates how the ETHOS model trained on a large dataset of PHTs to simulate potential future patient health timelines (fPHTs). By analyzing a particular patient's known PHT and generating multiple fPHTs, the model estimates the probabilities of critical outcomes, such as inpatient death, ICU admission, or a prolonged hospital stay exceeding 10 days. Panel 3 showcases the result of processing of fPHTs to calculate event-specific risks and predict the timing of these events, should they occur. Risk levels are defined across five categories, color-coded for enhanced clinical interpretability. Panel 4 showcases the explainability module, which identifies the key factors influencing specific risk estimates, offering personalized and actionable insights to support clinical decision-making. In this example, blue tokens indicate factors contributing to an increased risk of ICU admission.

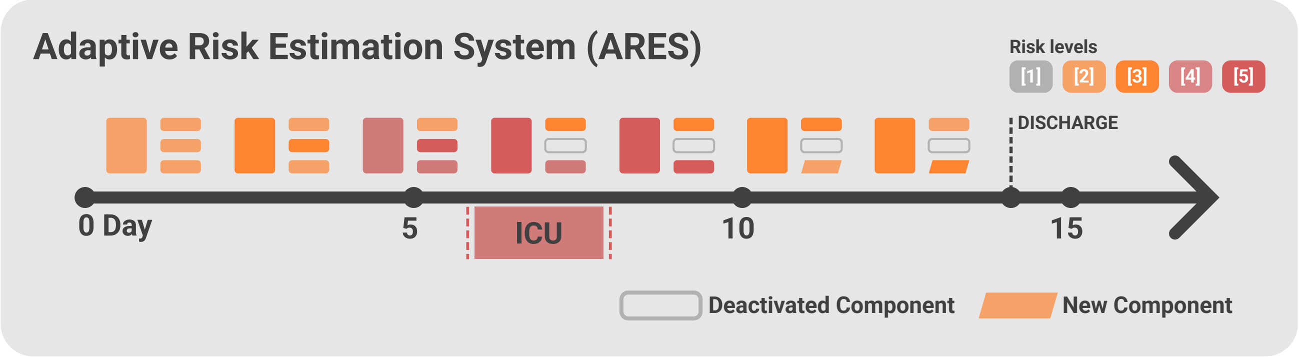

Figure 2. Timeline of a Patient's Hospital Stay and Hypothetical Risk Predictions by ARES. This figure illustrates the timeline of a patient's hospital stay, from admission to discharge around Day 14, demonstrating how ARES dynamically adjusts its predictions based on the patient's evolving clinical status and medical history. By Day 5, ARES predicts a high risk of ICU admission, which is subsequently confirmed as the patient is admitted around Day 6. Once the patient is in the ICU, ARES discontinues ICU risk evaluation, as indicated by the "Deactivated Component" label. After the ICU stay, ARES identifies an increased likelihood of a hospital stay exceeding 10 days. Upon reaching the 10-day threshold, ARES automatically recalibrates its predictions, replacing the previous risk estimation with the likelihood of a 15-day stay, now categorized as a "New Component" in the risk assessment.

the predictive capabilities of ETHOS, we compared its performance against traditional early warning scores and other ML models. Figure 3 presents the AUC values (ROC curves in Figure 2) for key ED benchmark tasks: hospitalization at triage, critical outcomes within 12 hours of triage, and ED re-presentation within 72 hours post-discharge. ETHOS demonstrated consistently superior predictive accuracy across all evaluated tasks. We provide detailed numerical values in the Supplement (Table 3,4,5).

The risks provided by ETHOS were also found to be well-calibrated, as tested by calibration curves. Brier scores were found in the range 0.01-0.14 depending on the task, indicating excellent to good performances, as shown in Figure 4.

Discussion

The ARES framework introduces an innovative approach to building predictive models by leveraging cutting-edge artificial intelligence technology. Several aspects of this approach distinguish it from traditional models. First, ARES enables dynamic risk estimation at any time during a patient's stay, from admission to discharge. Powered by ETHOS [15], ARES utilizes PHTs and incorporates all available clinical information at the time of risk estimation. Unlike traditional models, which rely on static data points such as information collected within 24 hours after admission or ED presentation or data up to triage [27, 28], ARES continuously adapts to the patient's evolving clinical status. This adaptability overcomes a key limitation of static models, which may not perform optimally outside the narrow time frames for which they are designed. This capability is demonstrated in the accompanying Figure 4 and Table 10, which illustrate how risk evolves over time during a patient's hospital stay. These visualizations, which depict how personalized risk evolves over time to reach the current estimates, provide insights into the specific factors driving model predictions for each patient. They highlight clinical events associated with increased or decreased risk, offering real-time explainability. By identifying the most influential features contributing to an individual's risk assessment, ARES have the potential to empower clinicians with a clearer understanding of the rationale behind each prediction.

As illustrated in Figure 1, ARES can estimate risk for various critical events, such as in-hospital mortality, ICU admission, and prolonged hospital stays. Beyond these standard metrics, additional indicators can be integrated seamlessly, including the risk of ICU admission during a specific length of stay, ICU readmission, acute kidney injury, sepsis, cardiac arrest, or 30-day readmission, and others. The ETHOS model, which underpins ARES, allows for the dynamic combination of these risks into composite measures while accounting for their interdependencies. For example, the occurrence of mortality on day 8 would render the probability of a 10-day hospital stay zero. This ability to incorporate conditional and causal relationships between tracked events is another strength of ARES. Importantly, integrating additional metrics does not require model retraining or modifications of ETHOS. Once a range of possible future PHTs has been generated, any additional metrics can be calculated with minimal computational resources, making ARES scalable and adaptable to diverse healthcare settings.

In its current implementation, ETHOS distills multiple fPHTs into a single predictive decision, such as inpatient mortality. However, this approach overlooks the wealth of longitudinal information contained in these trajectories, including the sequence of clinical events that lead to a particular outcome, or the absence thereof. By merely predicting the likelihood of an adverse event, valuable insights into the pathways that contribute to deterioration or recovery remain underutilized. Expanding ARES to provide a more granular, trajectory-based interpretation of risk would allow clinicians not only to assess a patient's probability of experiencing a critical event but also to understand the evolving clinical course leading to that outcome including the cost. This enhanced approach would address a key limitation highlighted in the early warning paradox [29], where models trained on retrospective data may fail to capture the full complexity of clinical interventions and their effects on patient outcomes. Moving forward, we aim to refine ARES to incorporate and visualize these probabilistic trajectories. This will equip clinicians with deeper, more actionable insights into clinical risk dynamics and potentially provide new information about causality in patient outcomes.

We recognize that, to date, ARES has not undergone formal usability testing with frontline clinicians, yet their ultimate impact depends on seamless integration into real-world workflows. Emergency medicine specialists on our team have provided informal feedback on feasibility and clarity of the risk estimates and explanatory highlights, and we are now designing pilot simulations in which physicians will "round" on de-identified patient cases presented through mock electronic charts powered by ARES. These studies, first leveraging MIMIC-derived timelines and subsequently our own Mass General Brigham data, will allow us to observe decision points, gather qualitative feedback on timing and interpretability of alerts, and refine both the user interface and explanation formats. We anticipate that iterative, case-based testing will guide the development of a clinician-centered dashboard, ensuring that ARES's predictions align with care priorities and support timely, actionable insights in the emergency setting.

This study has several important limitations. First, although we demonstrated ETHOS using PHTs derived from the MIMIC-IV-ED

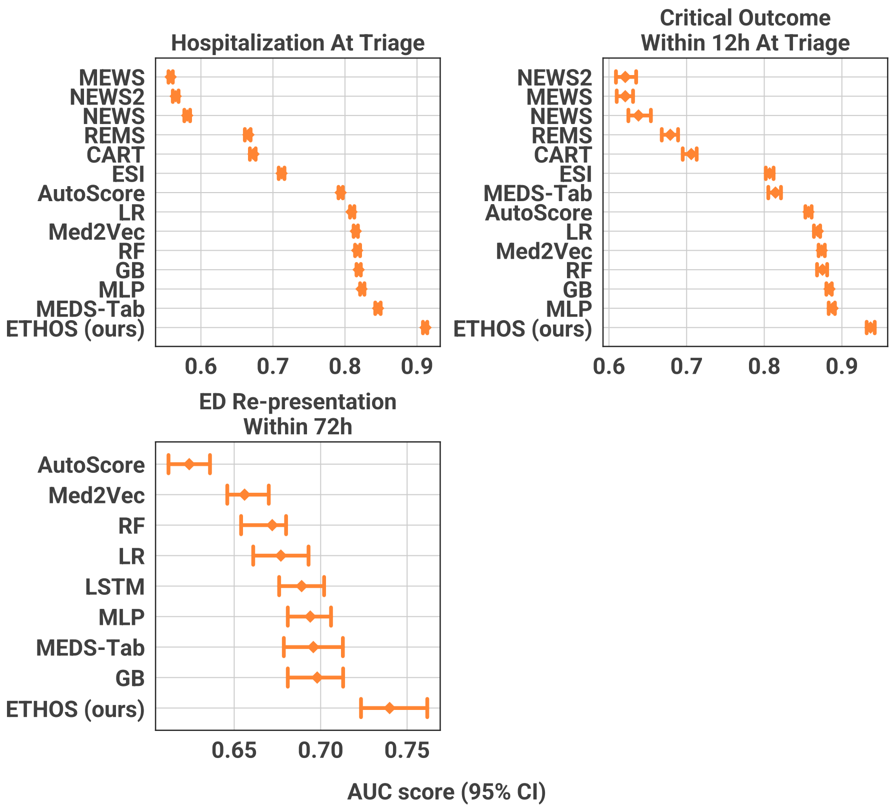

Figure 3. Predictive results for the ED benchmark tasks. Fewer methods appear in the ED re-presentation task (right) because score-based approaches, designed specifically to estimate in-hospital deterioration, are not applicable once the patient has left the ED. Ethos consistently achieves the best performance across all evaluated tasks.

dataset, its performance on data from other institutions may be compromised without retraining on external cohorts. Electronic health record systems and clinical workflows differ substantially across hospitals—driven by variations in documentation practices, patient case-mix, and care protocols—so models trained on one site can yield misleading risk estimates when deployed elsewhere. Moreover, our training data may harbor demographic and institutional biases (for example, overrepresentation of certain age, race, or socioeconomic groups), which could impair generalizability and exacerbate health inequities if unaddressed. We have not yet conducted a thorough fairness audit to quantify potential disparities in ETHOS's predictions across sex, race, or ethnicity. By contrast, in domains such as radiology or pathology, data inputs like images are relatively standardized, enabling easier cross-institution transfer. To facilitate broader validation and retraining, we have ensured that the ETHOS-ARES codebase is fully compatible with the MEDS health AI data standard [30]. This interoperability simplifies the process for other researchers to apply identical model architectures to their local data, perform bias and subgroup analyses, and iteratively refine ETHOS for diverse patient populations.

We also recognize that our evaluation on the extensively curated MIMIC-IV dataset may underestimate challenges encountered in real-world EHRs, which often exhibit higher rates of missing or irregular data, temporal shifts in documentation and care processes, and evolving patient populations. Although ETHOS is designed to operate on incomplete timelines, elevated missingness will still impair model accuracy. It is unclear if temporal biases or practice changes may introduce drift over time for ETHOS because the inferences are based on the context which contemporary to predictions, but this has not been investigated yet. Future work will systematically assess ETHOS's resilience to these factors and develop strategies for ongoing recalibration in heterogeneous clinical environments.

In current implementation, we exclude unstructured clinical text and because of that ETHOS may miss nuanced patient information, such as narrative impressions or social determinants, that could enhance risk estimation and zero-shot generalizability. Integrating free-text notes poses challenges in segmenting and embedding variable-length narratives alongside structured events without overwhelming the model's capacity. In future work, we will explore the use of pretrained clinical-language-model embeddings, hierarchical chunking of note content, and multimodal fusion techniques to incorporate these rich data into the PHTs.

Data standardization is often proposed as a solution to address the challenges of variability in healthcare data. However, achieving meaningful standardization would require identifying commonalities between healthcare systems, an endeavor that may not be feasible given the diversity of clinical practices, patient populations, and institutional workflows. An alternative is to train AI models, such as ETHOS, on raw data from diverse institutions, allowing the model itself to learn and interpret the underlying patterns and clinical pathways. This approach mirrors the capability of large language models to discern meaning from vastly different styles of text and presentations or even different languages, leveraging the same transformer architecture as ETHOS. We performed an energy consumption analysis comparing training of ETHOS to other known LLMs that can be seen in Table 9.

In summary, recent advances in AI have created unprecedented opportunities for innovative solutions like ARES, which harness large volumes of heterogeneous data to build general-purpose models whose predictive performance exceeds state-of-the-art methods. ARES delivers dynamic, personalized risk estimates and offers real-time explainability, empowering clinicians to make better-informed decisions. Moreover, its modular architecture and the underlying ETHOS framework enable seamless integration of additional data modalities, such as radiology, genomics, and other institutional datasets, further enhancing predictive accuracy and broadening applicability across diverse healthcare environments.

Although our results are promising, the clinical impact remains uncertain. Demonstrating ARES's true utility in real-world settings will be the focus of our future work.

As healthcare costs and complexity continue to rise, PHT-based frameworks like ARES show a promising pathway towards data-driven AI-enabled individualized patient care with the potential to reduce morbidity, improve outcomes, and lower healthcare costs.

Potential implications

The Adaptive Risk Estimation System (ARES) offers several near-term opportunities to enhance clinical care beyond emergency early warning. First, by continuously updating personalized risk profiles, ARES can inform dynamic triage and resource allocation decisions across hospital units. For example, bed managers could use the ARES scores to anticipate ICU demand several hours in advance, improving staff deployment and reducing admission delays.

Second, ARES's real-time explainability module—highlighting the specific tokens driving risk changes—can support shared decision-making at the bedside. Clinicians may review the key factors that elevated a patient's risk, facilitating targeted interventions such as order adjustments, specialist consults, or heightened monitoring, and enabling more transparent discussions with patients and families.

Third, ARES can serve as a decision-support tool in clinical research and quality improvement initiatives. Embedded within institutional dashboards, ARES could identify patient subgroups with unexpectedly high or low risk trajectories, prompting retrospective chart reviews or prospective studies to refine care pathways. Its use in pilot implementation studies may reveal workflow integrations that optimize alert timing and reduce alarm fatigue.

Methods

ETHOS and Probabilistic Inference

We introduced ETHOS in [15]. It operates on Patient Health Timelines (PHTs), which are tokenized chronological representations of patient medical histories (see Table 8). Here, tokenization refers to encoding clinical events, such as inpatient visits, procedures, laboratory results, medication administrations, and vital signs, as sequences of discrete tokens. The intervals between events are captured using specialized time-interval tokens. Formally, a PHT is a sequence of integer labels corresponding to these tokens, and its length can reach hundreds of thousands of tokens.

ETHOS employs a transformer-based generative model to predict future clinical events from tokenized PHTs. During inference, ETHOS generates successive tokens, each denoting a prospective future event, until a predefined stopping condition is met, such as the appearance of a target event token or the attainment of a simulation time limit. By repeatedly simulating multiple future PHTs (fPHTs) for each patient, ETHOS explores a range of possible trajectories, thereby quantifying the inherent uncertainty in its predictions. For instance, if N fPHTs are simulated and M of these trajectories include an inpatient mortality token, the estimated mortality probability is given by M/N (see section). All probabilistic inferences in this paper utilize Monte Carlo (MC) sampling with $N = 100$ simulated fPHTs per patient, which inevitably introduces variability due to finite draws. We quantify this uncertainty by modeling the number of positive outcomes as a $Binomial(N, p)$ random variable and computing 95% confidence intervals, and visualizing these as shaded bands around the mean risk trajectory (e.g., Figure 4).

For detailed information on the transformer architecture, PHT statistics, and tokenization procedures, as well as intuitive explanation of ETHOS, please refer to our first publication [15] and section .

Data Preprocessing

We extracted relevant data from the MIMIC-IV tables as detailed in Table 7. Laboratory tests and medications were standardized using ATC codes, and all diagnostic and procedural codes were mapped to ICD-10 when necessary, as described in [15]. Additional tables requiring advanced processing, such as clinical notes, were not included in the current implementation of ETHOS.

The dataset was split into two disjoint groups: training/validation (90%) and testing (10%). Exactly the same splits were used for all methods investigated.

Tokenization, PHT Construction, model training

The core of ETHOS lies in constructing PHTs from electronic medical records (EMRs) using a tokenization strategy that captures diverse clinical events. A PHT represents a patient's medical history as a sequence of tokens, each encoding specific health-related information organized chronologically. This structured representation enables comprehensive modeling of patient journeys and more accurate clinical predictions. To build PHTs, we used the MEDS-DEV [31] extraction pipeline that converts EHR data to an intermediate format called MEDS [30] to facilitate further data transformations. Advanced transformation operations were subsequently applied, breaking down each event into 1 to 7 tokens based on its complexity.

For example, lab test results were encoded using quantile-based tokens to represent clinical significance. Time-interval tokens were added to mark the elapsed time between successive events, with intervals shorter than 5 minutes omitted and longer gaps tokenized into 19 distinct interval tokens. Continuous numerical values, such as lab test results, were similarly quantile-encoded using ten quantiles, balancing clinical interpretability and predictive precision. Diagnostic and procedural codes, including ICD-10-CM, ICD-10-PCS, and ATC drug codes, were encoded hierarchically, which leveraged their inherent structure to enhance the transformer model's attention mechanisms. For more details, refer to [15].

Static patient attributes such as gender, marital status, race, and body mass index (BMI) were encoded using a single token depending on the value. For age, tokens of quantiles were reused, allowing age representation from 0 to 99. For instance, a 46-year-old patient would be coded as Q5 and Q7. Attributes with potential variability were represented using their most recently known value at the start of the timeline. By incorporating these elements, ETHOS ensured a richer and more adaptable representation of patient timelines.

During the training phase, 6 million tokens (1.8% of the train/validation dataset) were used for validation to balance model optimization and computational efficiency. The detailed statistics about the tokenized dataset are available in Table 6, 11, and information about the model is in Figure 1.

Explainability

As illustrated in Figure 4, stochastic simulations can be initiated not only from the most recent token representing current information but also from any preceding token in the patient's history. This allows risk estimates to be visualized as a time series, highlighting how specific medical events affect risk over time. This approach provides intuitive visualizations, offering clinicians clear insights into the factors contributing to current risk values.

Methods used for benchmarking

We followed benchmarking tasks for emergency department models presented in the Emergency Department MIMIC-IV-ED benchmark paper [27]. Three tasks were defined: prediction of the hospi-

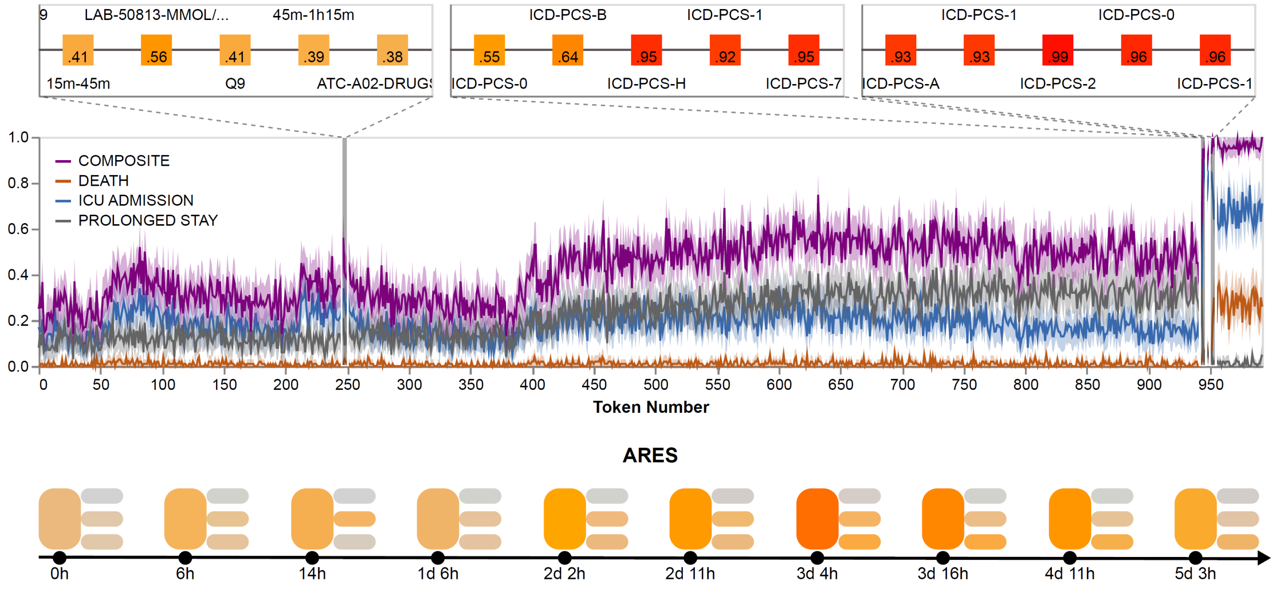

Figure 4. ARES Risk Trajectories. This figure illustrates risk trajectories for nearly 1000 tokens preceding patient death, as monitored by ARES, which evaluates the probability of death, ICU admission, prolonged hospital stay, and a composite risk score. The lower panel provides a color-coded representation of risk with the actual time since the ED presentation. In contrast, the upper panel highlights three 5-token regions influencing risk predictions at areas marked by the thin gray bar. In the first region, token LAB-50813 (Lactate Blood Test) increases the composite risk score from 0.41 to 0.56, but since the result falls in Q9 (80–90th percentile), ETHOS downgrades the risk estimate back to the previous level. In the second region (close to the end), a sharp increase in composite risk occurs due to heightened ICU admission triggered by ICD-PCS code 0BH17EZ, which is coded by 7 tokens (only 5 visible), which represents Endotracheal Airway Insertion into the Trachea via Natural or Artificial Opening. The 'H' token specifically signals ETHOS to escalate the ICU risk to nearly 1.0, indicating that the patient is being intubated de novo. The ICD-10-PCS breakdown confirms the procedure as a respiratory intervention involving tracheal insertion via a natural or artificial opening. ICD-PCS 0BH17EZ does not increase the risk of death, but the next ICD-PCS 5A12012 (5 tokens coding A1202 visible) raises the risk of death to about 0.25. We note that an increased risk of death is associated with a decreased risk of ICU admission, as these are competing risks. This visualization demonstrates how ARES dynamically adjusts risk scores based on evolving patient data, integrating clinical trajectories into real-time risk assessment. In this example, the rapid risk increases immediately following invasive procedures (e.g., intubation) should be interpreted as retrospective severity markers rather than actionable alerts, since they occur too late to guide effective intervention. Shaded bands around each trajectory denote the 95% confidence intervals arising from Monte Carlo sampling.

tal admission at triage, prediction of the critical outcome (death or transfer to ICU within 12 hours) at triage, and ED re-presentation within 72 hours after discharge from ED. We applied machine learning methods (logistic regression, random forest, gradient boosting), scoring systems MEWS [13], NEWS [12, 32, 33], Rapid Emergency Medicine Scores (REMS) [34], Cardiac Arrest Risk Triage (CART) [35], five-level triage system Emergency Severity Index (ESI) [36] and neural networks-based models including multilayer perceptron, Med2Vec [37] and Long Short-Term Memory (LSTM) [38].

To compare tasks used for early warning scores, we compared the MEDS-Tab library [39] which was used to establish a baseline. MEDS-Tab converts time-series EHR data into a tabular format by aggregating features across multiple time windows. It takes longitudinal patient data and applies various aggregation functions (like sum, count, min, max) over different historical window sizes to create fixed-size feature vectors, where each feature represents a combination of a medical code, time window, and aggregation method. XGBoost [40] models are trained on these tabular features computed from data windows prior to each prediction time point for each clinical task.

Statistical Methods

The performance of predictive models was evaluated using Receiver Operating Characteristic (ROC) curves and corresponding Area Under the Curve (AUC) values. Bootstrapping techniques were employed to estimate 95% confidence intervals (CIs) for AUCs. Model predicted probabilities were compared with observed event frequencies using calibration curves to evaluate ETHOS's reliability and alignment with real-world clinical outcomes. All statistical analyses were conducted using Python-based libraries, including scipy and scikit-learn [41, 42]. Data visualization, including ROC curves, calibration plots, and other statistical figures, was performed using matplotlib, seaborn and altair.

Availability of source code and requirements

- Project name: ETHOS-ARES
- Project home page: `https://github.com/ipolharvard/ethos-ares`
- Operating system(s): Platform independent
- Programming language: Python
- Other requirements: Polars, Pytorch etc. (see pyproject.toml)
- License: MIT

Data availability

The MIMIC-IV dataset is publicly available at `https://physionet.org/content/mimiciv/2.2`. Its Emergency Department extension is available at `https://physionet.org/content/mimic-iv-ed/2.2/`. These are controlled-access datasets; users need to sign in to PhysioNet, apply for a credentialed account, sign a Data Use Agreement, and follow training on human research data. Annotations to the code are available at ML-DOME [43].

Declarations

List of abbreviations

- **AI** – Artificial Intelligence
- **ARES** – Adaptive Risk Estimation System

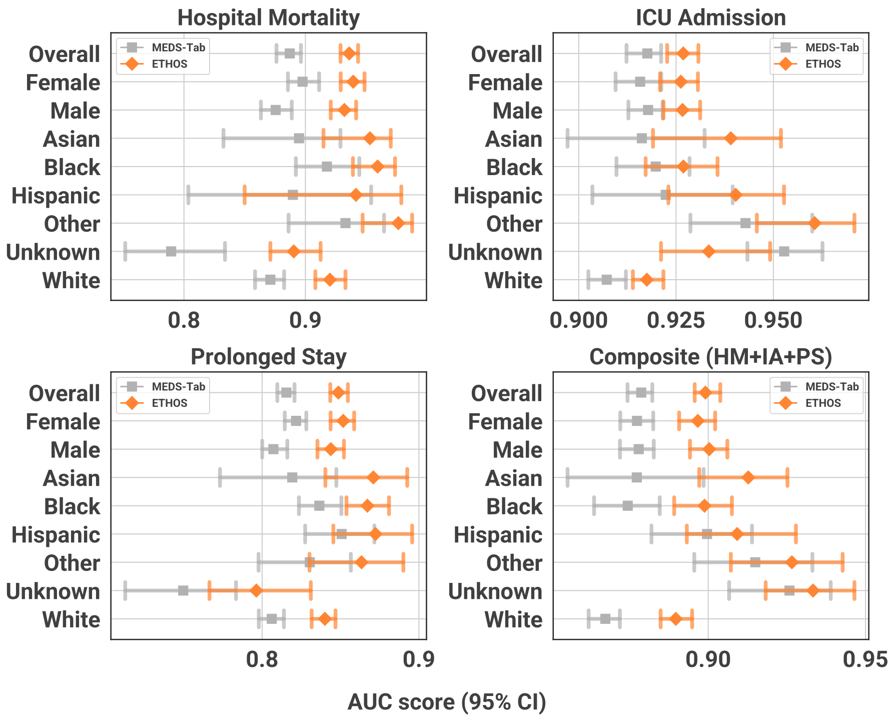

Figure 5. AUC Comparison Between ETHOS and MEDS-Tab Across Demographic Subgroups and Prediction Tasks. AUC scores with 95% confidence intervals are shown for ETHOS (orange) and MEDS-Tab (gray) across four prediction tasks: Hospital Mortality, ICU Admission, Prolonged Stay, and Composite Outcome (Hospital Mortality + ICU Admission + Prolonged Stay). Performance is reported for the overall population and stratified by gender (Female, Male) and race (Asian, Black, Hispanic, Other, Unknown, White). ETHOS consistently outperforms MEDS-Tab across all demographic subgroups and tasks.

- **ATC** – Anatomical Therapeutic Chemical (codes)
- **AUC** – Area Under the Curve
- **BIDMC** – Beth Israel Deaconess Medical Center
- **BMI** – Body Mass Index
- **CART** – Cardiac Arrest Risk Triage
- **CI** – Confidence Interval
- **ED** – Emergency Department
- **EHR** – Electronic Health Record
- **EMR** – Electronic Medical Record
- **ESI** – Emergency Severity Index
- **ETHOS** – Enhanced Transformer for Health Outcome Simulation
- **fPHT** – Future Patient Health Timeline
- **GDP** – Gross Domestic Product
- **ICD-10** – International Classification of Diseases, 10th Revision
- **ICU** – Intensive Care Unit
- **LLM** – Large Language Model
- **LSTM** – Long Short-Term Memory
- **MEDS** – Medical Event Data Standard
- **MEWS** – Modified Early Warning Score
- **MIMIC-IV** – Medical Information Mart for Intensive Care, version IV
- **ML** – Machine Learning
- **NEWS** – National Early Warning Score
- **PHT** – Patient Health Timeline
- **REMS** – Rapid Emergency Medicine Score
- **ROC** – Receiver Operating Characteristic
- **XGBoost** – eXtreme Gradient Boosting

Consent for publication

Not applicable

Competing Interests

Dr. Jia is also affiliated with Verily Life Science. Dr. Cunningham reports consultancy for Edgewise Therapeutics, Occlutech, and us2.ai. Dr. Bates reports grants and personal fees from EarlySense, personal fees from CDI Negev, equity from ValeraHealth, equity from Clew, equity from MDClone, personal fees and equity from AESOP, personal fees and equity from FeelBetter, personal fees and equity from Guided Clinical Solutions, outside the submitted work. Other authors declare no competing interests.

Funding

Dr. Sitek reports grant support NIH R01HL159183, and Dr. Cunningham reports grant support from the American Heart Association (23CDA1052151) and the National Heart, Lung, and Blood Institute (1K23HL168163).

Author's Contributions

- **PR** – Conceptualization, Methodology, Formal Analysis(lead), Visualization, Software(lead), Writing—Review, and Editing.
- **MKG** – Conceptualization, Methodology, Formal Analysis, Writing—Review, Editing, and Visualization.
- **NO** – Writing—Review, Formal Analysis.
- **DG** – Writing–Review, and Editing.
- **YJ** – Methodology, Writing–Review, and Editing.
- **SB** – Conceptualization.
- **MBAM** – Methodology, Writing–Review, and Editing.
- **JW** – Writing–Review, and Editing.
- **AES** – Writing–Review, and Editing.
- **JWC** – Writing–Review, and Editing.
- **DWB** – Writing–Review, and Editing.
- **AS** – Conceptualization(lead), Methodology, Writing–Original Draft, Preparation, and Supervision.

Acknowledgements

We thank Kinga Renc, M.Arch, for her invaluable assistance with graphic design.

References

1. Centers for Medicare & Medicaid Services, National Health Expenditure Data: Historical; 2024. Accessed: 2025-05-03. https://www.cms.gov/data-research/statistics-trends-and-reports/national-health-expenditure-data/historical.
2. Gunja MZ, Gumas ED, Williams RD II, U.S. Health Care from a Global Perspective, 2022: Accelerating Spending, Worsening Outcomes; 2023. Accessed: 2025-1-26. https://www.commonwealthfund.org/publications/issue-briefs/2023/jan/us-health-care-global-perspective-2022.
3. Committee on the Future of Emergency Care in the United States Health System . Hospital-based emergency care: at the breaking point. Washington, D.C., DC: National Academies Press; 2007.
4. Yang KK, Lam SSW, Low JMW, Ong MEH. Managing emergency department crowding through improved triaging and resource allocation. Oper Res Health Care 2016 Sep;10:13–22.
5. Horton DJ, Graves KK, Kukhareva PV, Johnson SA, Cedillo M, Sanford M, et al. Modified early warning score-based clinical decision support: cost impact and clinical outcomes in sepsis. JAMIA Open 2020 Jul;3(2):261–268.
6. Adams R, Henry KE, Sridharan A, Soleimani H, Zhan A, Rawat N, et al. Prospective, multi-site study of patient outcomes after implementation of the TREWS machine learning-based early warning system for sepsis. Nat Med 2022 Jul;28(7):1455–1460.
7. Edelson DP, Churpek MM, Carey KA, Lin Z, Huang C, Siner JM, et al. Early warning scores with and without artificial intelligence. JAMA Netw Open 2024 Oct;7(10):e2438986.
8. Gerry S, Bonnici T, Birks J, Kirtley S, Virdee PS, Watkinson PJ, et al. Early warning scores for detecting deterioration in adult hospital patients: systematic review and critical appraisal of methodology. BMJ 2020 May;369:m1501.
9. Winslow CJ, Edelson DP, Churpek MM, Taneja M, Shah NS, Datta A, et al. The impact of a machine learning early warning score on hospital mortality: A multicenter clinical intervention trial. Crit Care Med 2022 Sep;50(9):1339–1347.
10. Escobar GJ, Liu VX, Schuler A, Lawson B, Greene JD, Kipnis P. Automated identification of adults at risk for in-hospital clinical deterioration. N Engl J Med 2020 Nov;383(20):1951–1960.
11. Cummings BC, Blackmer JM, Motyka JR, Farzaneh N, Cao L, Bisco EL, et al. External validation and comparison of a general ward deterioration index between diversely different health systems. Crit Care Med 2023 Jun;51(6):775–786.
12. Williams B. The National Early Warning Score: from concept to NHS implementation. Clin Med 2022 Nov;22(6):499–505.
13. Subbe CP, Kruger M, Rutherford P, Gemmel L. Validation of a modified Early Warning Score in medical admissions. QJM 2001 Oct;94(10):521–526.
14. Vaswani A, Shazeer N, Parmar N, Uszkoreit J, Jones L, Gomez AN, et al. Attention is all you need. Adv Neural Inf Process Syst 2017;30.
15. Renc P, Jia Y, Samir AE, Was J, Li Q, Bates DW, et al. Zero shot health trajectory prediction using transformer. NPJ Digit Med 2024 Sep;7(1):256.
16. Yang Z, Mitra A, Liu W, Berlowitz D, Yu H. TransformEHR: transformer-based encoder-decoder generative model to enhance prediction of disease outcomes using electronic health records. Nat Commun 2023 Nov;14(1):7857.
17. Li Y, Mamouei M, Salimi-Khorshidi G, Rao S, Hassaine A, Canoy D, et al. Hi-BEHRT: Hierarchical Transformer-Based Model for Accurate Prediction of Clinical Events Using Multimodal Longitudinal Electronic Health Records. IEEE J Biomed Health Inform 2023 Feb;27(2):1106–1117.
18. Luo X, Rechardt A, Sun G, Nejad KK, Yáñez F, Yilmaz B, et al. Large language models surpass human experts in predicting neuroscience results. Nat Hum Behav 2024 Nov;.
19. Thirunavukarasu AJ, Ting DSJ, Elangovan K, Gutierrez L, Tan TF, Ting DSW. Large language models in medicine. Nat Med 2023 Aug;29(8):1930–1940.
20. Kraljevic Z, Bean D, Shek A, Bendayan R, Hemingway H, Yeung JA, et al. Foresight-a generative pretrained transformer for modelling of patient timelines using electronic health records: a retrospective modelling study. Lancet Digit Health 2024 Apr;6(4):e281–e290.
21. McDermott MBA, Nestor BA, Argaw P, Kohane I. Event Stream GPT: A data pre-processing and modeling library for generative, pre-trained transformers over continuous-time sequences of complex events. Adv Neural Inf Process Syst 2023 Jun;abs/2306.11547.
22. Steinberg E, Fries J, Xu Y, Shah N. MOTOR: A time-to-event foundation model for structured medical records. arXiv [csLG] 2023 Jan;.
23. Li Y, Rao S, Solares JRA, Hassaine A, Ramakrishnan R, Canoy D, et al. BEHRT: Transformer for electronic health records. Sci Rep 2020 Apr;10(1):7155.
24. Jeong H, Oufattole N, Mcdermott M, Balagopalan A, Jangeesingh B, Ghassemi M, et al. Event-Based Contrastive Learning for medical time series. arXiv [csLG] 2023 Dec;.
25. Johnson AEW, Bulgarelli L, Shen L, Gayles A, Shammout A, Horng S, et al. MIMIC-IV, a freely accessible electronic health record dataset. Sci Data 2023 Jan;10(1):1.
26. Johnson A, Bulgarelli L, Pollard T, Horng S, Celi LA, Mark R. Mimic-iv. PhysioNet Available online at: https://physionet org/content/mimiciv/22/(accessed Oct 1, 2023) 2023;.
27. Xie F, Zhou J, Lee JW, Tan M, Li S, Rajnthern LS, et al. Benchmarking emergency department prediction models with machine learning and public electronic health records. Sci Data 2022 Oct;9(1):658.
28. Meng C, Trinh L, Xu N, Enouen J, Liu Y. Interpretability and fairness evaluation of deep learning models on MIMIC-IV dataset. Sci Rep 2022 May;12(1):7166.
29. Logan Ellis H, Palmer E, Teo JT, Whyte M, Rockwood K, Ibrahim Z. The early warning paradox. NPJ Digit Med 2025 Feb;8(1):81.
30. Arnrich B, Choi E, Fries JA, McDermott MBA, Oh J, Pollard T, et al. Medical Event Data Standard (MEDS): Facilitating Machine Learning for Health. In: ICLR 2024 Workshop on Learning from Time Series For Health; 2024. .
31. MEDS-DEV: Establishing Reproducibility and Comparability in Health AI;. https://github.com/mmcdermott/MEDS-DEV.
32. Smith GB, Prytherch DR, Meredith P, Schmidt PE, Featherstone PI. The ability of the National Early Warning Score (NEWS) to discriminate patients at risk of early cardiac arrest, unanticipated intensive care unit admission, and death. Resuscitation 2013 Apr;84(4):465–470.
33. Zhang S, Xu Y, Usuyama N, Xu H, Bagga J, Tinn R, et al. A multimodal biomedical foundation model trained from fifteen million image–text pairs. NEJM AI 2025 Jan;2(1).
34. Olsson T, Terent A, Lind L. Rapid Emergency Medicine score: a new prognostic tool for in-hospital mortality in nonsurgical emergency department patients. J Intern Med 2004 May;255(5):579–587.
35. Churpek MM, Yuen TC, Park SY, Meltzer DO, Hall JB, Edelson DP. Derivation of a cardiac arrest prediction model using ward vital signs. Crit Care Med 2012 Jul;40(7):2102–2108.
36. Eitel DR, Travers DA, Rosenau AM, Gilboy N, Wuerz RC. The

Emergency Severity Index triage algorithm version 2 is reliable and valid. Acad Emerg Med 2003 Oct;10(10):1070–1080.

37. Choi E, Bahadori MT, Searles E, Coffey C, Thompson M, Bost J, et al. Multi-layer representation learning for medical concepts. In: Proceedings of the 22nd ACM SIGKDD International Conference on Knowledge Discovery and Data Mining New York, NY, USA: ACM; 2016. .
38. Hochreiter S, Schmidhuber J. Long short-term memory. Neural Comput 1997 Nov;9(8):1735–1780.
39. Oufattole N, Bergamaschi T, Kolo A, Jeong H, Gaggin H, Stultz CM, et al. MEDS-Tab: Automated tabularization and baseline methods for MEDS datasets. arXiv [csLG] 2024 Oct;.
40. Chen T, Guestrin C. XGBoost: A Scalable Tree Boosting System. In: Proceedings of the 22nd ACM SIGKDD International Conference on Knowledge Discovery and Data Mining KDD '16, New York, NY, USA: Association for Computing Machinery; 2016. p. 785–794.
41. Virtanen P, Gommers R, Oliphant TE, Haberland M, Reddy T, Cournapeau D, et al. SciPy 1.0: fundamental algorithms for scientific computing in Python. Nat Methods 2020 Mar;17(3):261–272.
42. Pedregosa F, Varoquaux G, Gramfort A, Michel V, Thirion B, Grisel O, et al. Scikit-learn: Machine learning in python journal of machine learning research. Journal of machine learning research 2011;12:2825–2830.
43. Renc P, Grzeszczyk MK, Oufattole N, Goode D, Jia Y, Biegan-ski S, et al., Foundation Model of Electronic Medical Records for Adaptive Risk Estimation. [DOME-ML Annotations]; 2025. https://registry.dome-ml.org/review/jdqxjqrnsb. DOME-ML Registry.
44. Xie F, Chakraborty B, Ong MEH, Goldstein BA, Liu N, et al. AutoScore: a machine learning–based automatic clinical score generator and its application to mortality prediction using electronic health records. JMIR medical informatics 2020;8(10):e21798.

Click here to access/download
Supplementary Material
ETHOS_ARES___GigaScience___Resubmission_supplement.pdf

Personal Cover
Click here to access/download;Personal Cover;Cover_letter_gigascience_resubmission.docx.pdf

MASSACHUSETTS GENERAL HOSPITAL

HARVARD MEDICAL SCHOOL

Radiology Department,
Massachusetts General Hospital, Harvard Medical School
100 Cambridge St., Boston, MA 02114 Suite 1303
asitek@@mgh.harvard.edu

Arkadiusz Sitek, PhD
Associate Investigator
Massachusetts General Hospital
Member of Faculty
Harvard Medical School

Boston, May 7, 2025

Prof. Scott Edmunds, PhD
Editor-in-Chief
GigaScience Press,
BGI Hong Kong, Hong Kong

Dear Prof. Scott Edmunds,

We are pleased to resubmit our revised manuscript, *"Foundation Model of Electronic Medical Records for Adaptive Risk Estimation,"* to *GigaScience*. We appreciate the thoughtful and constructive feedback from the reviewers and editorial team, and we have made extensive revisions to address each point raised.

In this work, we present ETHOS, a transformer-based foundation model trained on longitudinal electronic health records (EHRs), and ARES, a flexible and explainable early warning system that computes personalized risk estimates in real time. ETHOS supports zero-shot inference across diverse clinical endpoints without the need for retraining. Our framework is trained on the publicly available MIMIC-IV database, and we provide complete source code and documentation to promote reproducibility and transparency.

In response to reviewer feedback, we have implemented the following key changes:

- **Expanded methodological details** on ETHOS training, inference procedures, and probabilistic modeling, including sampling variability and risk quantification.
- **Clarified the distinction between ETHOS and ARES**, with improved explanations and updated figures to reduce confusion between the foundation model and its application.
- **Revised the evaluation framework**, including AUPRC plots, statistical confidence intervals, and enhanced descriptions of all benchmark methods and preprocessing pipelines.
- **Addressed clinical considerations**, such as the limitations of last-minute alerts, the role of composite outcomes, and the clinical utility of different lead times.
- **Added discussions on limitations**, including data missingness, temporal bias, model drift, and the current exclusion of clinical text. We outline ongoing efforts to integrate unstructured data and generalize the model to additional settings.
- **Substantiated the foundation model claim**, aligning with the accepted definition in the AI community and emphasizing ETHOS's scale, multi-task adaptability, and zero-shot capabilities.

We have also corrected minor formatting issues, improved reference completeness, and updated the "Potential Implications" section. A detailed point-by-point response to each reviewer is included with the resubmission.

We believe the revised manuscript is substantially improved and now meets the high standards of *GigaScience*. We hope the updated work will be of strong interest to your readers, especially those engaged in clinical AI, healthcare modeling, and real-world deployment of machine learning in medicine.

Thank you for considering our revised submission. We look forward to your response.

Sincerely,

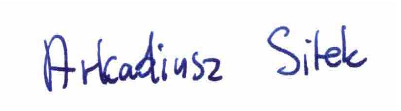

Arkadiusz Sitek, Ph.D.
Associate Investigator, Massachusetts General Hospital
Member of Faculty, Harvard Medical School
on behalf of all co-authors
