# Supplementary material for: Foundation model of electronic medical records for adaptive risk estimation
Source: Gigascience. 2025 Sep 30;14:giaf107. doi: 10.1093/gigascience/giaf107 (PMC12482913; doi:10.1093/gigascience/giaf107)
Supplement: giaf107_GIGA-D-25-00088_Revision_2 [file giaf107_giga-d-25-00088_revision_2.pdf]

GigaScience
Foundation Model of Electronic Medical Records for Adaptive Risk Estimation
--Manuscript Draft--

**Manuscript Number:**	GIGA-D-25-00088R2
**Full Title:**	Foundation Model of Electronic Medical Records for Adaptive Risk Estimation
**Article Type:**	Research
**Funding Information:**	NHLBI Division of Intramural Research (R01HL159183) — Not applicable
**Abstract:**	Background, Hospitals struggle to predict critical outcomes. Traditional early warning systems, like NEWS and MEWS, rely on static variables and fixed thresholds, limiting their adaptability, accuracy, and personalization; Methods, We previously developed the Enhanced Transformer for Health Outcome Simulation (ETHOS), an AI model that tokenizes patient health timelines (PHTs) from EHRs and uses transformer-based architectures to predict future PHTs. ETHOS is a versatile framework for developing a wide range of applications. In this work, we develop the Adaptive Risk Estimation System (ARES) that leverages ETHOS to compute dynamic, personalized risk probabilities for clinician-defined critical events. ARES also features a personalized explainability module that highlights key clinical factors influencing risk estimates. We evaluated ARES using the MIMIC-IV v2.2 dataset together with its Emergency Department (ED) extension and benchmarked performance against both classical early warning systems and contemporary machine learning models.; Results, The entire dataset was tokenized resulting in 285,622 PHTs (63% with at least one hospital admission), comprising over 357 million tokens. ETHOS outperformed benchmark models in predicting hospital admissions, ICU admissions, and prolonged stays, achieving superior AUC scores. Its risk estimates were robust across demographic subgroups, with calibration curves confirming model reliability. The explainability module provided valuable insights into patient-specific risk factors; Conclusions, ARES, powered by ETHOS, advances predictive healthcare AI by delivering dynamic, real-time, personalized risk estimation with patient-specific explainability. Although our results are promising, the clinical impact remains uncertain. Demonstrating ARES’s true utility in real-world settings will be the focus of our future work.
**Corresponding Author:**	Arkadiusz Sitek, Ph.D.<br>Massachusetts General Hospital<br>Sommerville, Massachusetts UNITED STATES
**Corresponding Author Secondary Information:**	
**Corresponding Author's Institution:**	Massachusetts General Hospital
**Corresponding Author's Secondary Institution:**	
**First Author:**	Pawel Renc
**First Author Secondary Information:**	
**Order of Authors:**	Pawel Renc
	Michal K Grzeszczyk
	Nassim Oufattole
	Deirdre Goode
	Yugang Jia
	Szymon Bieganski
	Matthew BA McDermott
	Jaroslaw Was
	Anthony E Samir

Powered by Editorial Manager® and ProduXion Manager® from Aries Systems Corporation

	David W Bates
	Jonathan W Cunningham
	Arkadiusz Sitek, Ph.D.
**Order of Authors Secondary Information:**	
**Response to Reviewers:**	Authors comments:<br>We thank the reviewer #1 for their comments and help with clarifications on our responses to reviewer #4.<br><br>Reviewer reports:<br><br>Reviewer #1: thank you for your satisfactory response to our comments.<br>The only point that remains unclear is the question about the loss of<br>a significant number of files. The authors explain this selection by<br>the tokenization process, which is a satisfactory answer. But they<br>don't discuss at all who are these patients excluded from the<br>analysis? Are they representative of the other patients, or is there a<br>selection bias that could undermine the generalizability of the<br>results?<br>this point should be discussed in the article<br>Otherwise, no comments.<br><br>Authors' response:<br><br>Patients were excluded if no information was available beyond the patient ID. After tokenization, any patient whose sequence contained no tokens between static tokens and timeline_end was excluded. This is because some patients in MIMIC have little or no usable information. Even if a patient has certain data, if that data type is not tokenized in the current version of ETHOS, the resulting tokenized timeline will have a length of zero, leading to exclusion. We explicitly discuss this in Evaluation (Page 2).<br><br><br>Reviewer reports:<br><br>Reviewer #4: Unfortunately reviewer #4 is not able to re-review the<br>manuscript. Reviewer #1 kindly helps to look at these additional<br>comments and responses as requested by the editor.<br><br>- On the foundation model aspect (questions 2 and 6): the reviewer's<br>question is very pertinent and the answer rather inadequate. A<br>foundation model is a model trained on a very large number of cases,<br>enabling results to be generalized to other cases without re-training.<br>The example given by the reviewer is trained on 28 M patients, and<br>that of the article on 300 k patients, which is much smaller and<br>suggests a lack of generalization. The answer given argues that the<br>granularity of the variables is much greater in the article's model<br>and that the data set is therefore larger, with consequently more<br>parameters on the model than for a foundation model as indicated by<br>the reviewer. What doesn't fit is that in the article's dataset, the<br>variables belonging to the same patient are not independent... so it<br>can't be argued that it's equivalent for training a model to have 1000<br>variables for each of the 300k patients and 10 variables for each of<br>the 28M patients. (this is what the<br>author maintains).<br><br>Authors' response:<br><br>We fully agree that a model trained on 300,000 patients cannot match the generalization potential of a model trained on 28 million patients, such as MedBERT. This limitation, which we acknowledge in the Discussion section, reflects the current scale of our dataset rather than an inherent limitation of our proposed method. Our approach is explicitly designed to scale, and we intend to incorporate larger and more diverse datasets in future work to enhance generalizability.

Powered by Editorial Manager® and ProduXion Manager® from Aries Systems Corporation

	In our initial response, we sought to clarify a separate point raised by Reviewer #4, who suggested that our model's parameter count is "relatively small," which was not true, and implied that the smaller patient cohort undermines its relevance compared to existing large-scale models. Our intention was not to equate 300k patients with 28M, but to explain that the complexity and richness of our input representation results in a model with higher representational capacity than might be expected purely based on patient count. Unlike models trained on sparse, flat representations, ours leverages dense, longitudinal sequences with fine-grained granularity, contributing to a higher parameter count and learning capacity. This was the purpose of our response to rev #4.<br>However, we acknowledge the reviewer's correct observation: while each patient in our dataset contributes multiple variables, these are not independent and do not fully compensate for the much larger cohort size in terms of generalization. This distinction is critical, and we thank the reviewer for pointing it out. We clarify in limitations in the discussion (Page 5) that our implementation has limitations of a relatively small size dataset which may prevent generalisation.<br><br>Reviewer reports:<br><br>- About benchmarks (question 3): we are not familiar with the MedBert and clinicalBert models. The authors have made no effort to convert the dataset to be compatible with these models. It's true that this is a pity, as they are closer to the article's model than a logistic regression. Nevertheless, we don't know whether this adaptation represents a lot of work time, as the authors say in their answer, or whether it can be done in a reasonable time. This would be a great benchmark, but may be difficult to achieve? We propose not to make this a blocking point for this submission and simply reiterate to the authors the interest for readers of this additional work.<br><br>Authors' response:<br><br>We thank the reviewer for the thoughtful suggestion to benchmark against MedBERT and ClinicalBERT. While we agree that such a comparison could be informative if conducted properly, the primary focus of our study lies elsewhere. Our core contribution is the development of a model that computes patient-specific risk dynamically, updating predictions as new clinical information becomes available rather than relying on static snapshots of patient variables.<br>We also introduce a novel explainability module within the ETHOS framework, enabling real-time interpretability of evolving risk estimates. Although we evaluated our approach on the ED-MIMIC dataset to demonstrate performance on an established benchmark, this was intended as supportive rather than central. Our primary aim was to show that ETHOS is competitive on standard tasks while uniquely enabling adaptive, temporally resolved risk prediction, capabilities not readily addressed by existing models, including those typically used for benchmarking.<br>Even if another model were to match or slightly exceed our performance on static benchmarks, it would not diminish our main contribution: a dynamic, explainable model that adapts to evolving clinical data in real time. In the revised version we note in limitations, our benchmarking employed classical risk-prediction methods; more sophisticated approaches could yield higher benchmark scores.<br>We appreciate the reviewer's agreement that this is not a barrier to publication and take their feedback as encouragement to extend our benchmarking to additional models in future work.
**Additional Information:**	
**Question**	**Response**
Are you submitting this manuscript to a special series or article collection?	No
**Experimental design and statistics**	Yes

Powered by Editorial Manager® and ProduXion Manager® from Aries Systems Corporation

Full details of the experimental design and statistical methods used should be given in the Methods section, as detailed in our Minimum Standards Reporting Checklist. Information essential to interpreting the data presented should be made available in the figure legends.<br><br>Have you included all the information requested in your manuscript?	
**Resources**<br><br>A description of all resources used, including antibodies, cell lines, animals and software tools, with enough information to allow them to be uniquely identified, should be included in the Methods section. Authors are strongly encouraged to cite Research Resource Identifiers (RRIDs) for antibodies, model organisms and tools, where possible.<br><br>Have you included the information requested as detailed in our Minimum Standards Reporting Checklist?	Yes
**Availability of data and materials**<br><br>All datasets and code on which the conclusions of the paper rely must be either included in your submission or deposited in publicly available repositories (where available and ethically appropriate), referencing such data using a unique identifier in the references and in the "Availability of Data and Materials" section of your manuscript.<br><br>Have you have met the above requirement as detailed in our Minimum Standards Reporting Checklist?	Yes
GigaScience has policies and guidelines in place for the use of generative AI-	No

writing tools such as ChatGPT. If you have used such writing tools to assist with writing the manuscript this must be declared and cited in the text. Authors should not list AI-writing tools and other AI-assisted technologies as an author or co-author and should acknowledge that they are fully responsible for text generated or refined by AI-writing tools.<p><br><br>A summary of use (particularly in the introduction or among methods) needs to be included at the end of the paper, and the outputs should also be included as a supplementary file hosted in GigaDB or other open repositories. Please read our guidelines for more information. <p><br><br>By submitting to GigaScience, you are aware of the journal's AI-writing tools policy, and if you have declared use of such tools below, you have acknowledged this where appropriate in your manuscript and have made a summary of use and outputs available. <p><br><b>AI-assisted writing tools have been used in the preparation of this manuscript?	

Manuscript

Click here to access/download;Manuscript;ETHOS_ARES___GigaScience_

Placeholder for OUP logo oup.pdf

GigaScience, 2023, 1–13

doi: xx.xxxx/xxxx
Manuscript in Preparation
Paper

PAPER

Foundation Model of Electronic Medical Records for Adaptive Risk Estimation

Pawel Renc[1,2,3], Michal K. Grzeszczyk[1,2], Nassim Oufattole[4], Deirdre Goode[8,2], Yugang Jia[4], Szymon Bieganski[6], Matthew B. A. McDermott[2], Jaroslaw Was[3], Anthony E. Samir[1,2], Jonathan W. Cunningham[5,2], David W. Bates[5,7,2] and Arkadiusz Sitek[1,2,*]

[1]Massachusetts General Hospital, Boston, USA and [2]Harvard Medical School, Boston, USA and [3]AGH University of Krakow, Krakow, Poland and [4]Massachusetts Institute of Technology, Cambridge, USA and [5]Brigham and Women's Hospital, Boston, USA and [6]Medical University of Lodz, Lodz, Poland and [7]Harvard Chan School of Public Health, Boston, USA and [8]Newton Wellesley Hospital, Newton, USA

[*] Corresponding author: Arkadiusz Sitek, sarkadiu@gmail.com

Abstract

Background, Hospitals struggle to predict critical outcomes. Traditional early warning systems, like NEWS and MEWS, rely on static variables and fixed thresholds, limiting their adaptability, accuracy, and personalization; **Methods,** We previously developed the Enhanced Transformer for Health Outcome Simulation (ETHOS), an AI model that tokenizes patient health timelines (PHTs) from EHRs and uses transformer-based architectures to predict future PHTs. ETHOS is a versatile framework for developing a wide range of applications. In this work, we develop the Adaptive Risk Estimation System (ARES) that leverages ETHOS to compute dynamic, personalized risk probabilities for clinician-defined critical events. ARES also features a personalized explainability module that highlights key clinical factors influencing risk estimates. We evaluated ARES using the MIMIC-IV v2.2 dataset together with its Emergency Department (ED) extension and benchmarked performance against both classical early warning systems and contemporary machine learning models.; **Results,** The entire dataset was tokenized resulting in 285,622 PHTs (63% with at least one hospital admission), comprising over 357 million tokens. ETHOS outperformed benchmark models in predicting hospital admissions, ICU admissions, and prolonged stays, achieving superior AUC scores. Its risk estimates were robust across demographic subgroups, with calibration curves confirming model reliability. The explainability module provided valuable insights into patient-specific risk factors; **Conclusions,** ARES, powered by ETHOS, advances predictive healthcare AI by delivering dynamic, real-time, personalized risk estimation with patient-specific explainability. Although our results are promising, the clinical impact remains uncertain. Demonstrating ARES's true utility in real-world settings will be the focus of our future work.

Key words: Early Warning Scores; EHR; Foundation Model; Transformer; Zero-Shot Inference; Patient Health Trajectories

Background

The United States allocates nearly 18 percent of its GDP to healthcare [1], yet Americans have shorter lifespans and poorer health than residents of other high-income nations. Among these countries, the U.S. not only has the lowest life expectancy but also the highest rates of preventable deaths [2]. Hospitals face mounting challenges managing patient influx and identifying individuals at risk for critical outcomes, including mortality, intensive care unit (ICU) admission, or prolonged hospital stays [3]. Accurate prediction of critical clinical events is essential for enhancing patient care and optimizing the timely allocation of limited healthcare resources [4]. Early identification of at-risk patients

Compiled on: August 5, 2025.
Draft manuscript prepared by the author.

Key Points

- ARES enables dynamic, real-time risk estimation by predicting patient health timelines (PHTs) from electronic health records using a transformer-based model.
- ARES enhances clinical decision-making by leveraging ETHOS-generated future PHTs to provide personalized risk predictions with explainability.
- Methods used outperform traditional models in predicting critical outcomes while demonstrating strong calibration and equitable performance across demographic subgroups.

enables clinicians to prioritize interventions, anticipate potential escalations in care, and improve outcomes while simultaneously reducing costs [5, 6]. However, current methodologies often fail to fully utilize the vast and complex data available in electronic health records (EHRs), a limitation that becomes particularly evident in emergency settings where time-sensitive decisions are critical [7, 8, 9, 10, 11]. Traditional scoring systems, such as the National Early Warning Score (NEWS) [12] and the Modified Early Warning Score (MEWS) [13], rely on static variables and predefined thresholds, constraining their ability to adapt to dynamic and multifaceted patient data. These approaches are further hindered by their reliance on specific cutoff points for data inclusion (e.g., triage, 24-hour windows), which can overlook valuable longitudinal patterns.

Recent advances in generative machine learning—particularly transformer architectures [14, 15, 16, 17] that underpin Large Language Models [18, 19]—have unlocked unprecedented capabilities for processing high-dimensional, heterogeneous, time-stamped health data from EHRs [20, 21, 22, 23, 24]. In this work, we build on our Enhanced Transformer for Health Outcome Simulation (ETHOS) [15], which differs from prior efforts in its tokenization and handling of EHR events. ETHOS is autoregressively pretrained—without any task-specific labels—on over 321 million tokens drawn from 269,741 Patient Health Timelines (PHTs), learning broad, high-dimensional representations that transfer across tasks. Operating on PHTs (tokenized sequences of demographics, diagnoses, medications, etc.; see Table 7), ETHOS generates plausible future timelines (Figure 1) and delivers zero-shot predictions for mortality, ICU admission, prolonged stay, and composite endpoints without any additional fine-tuning. By virtue of its scale, generalizability, and multi-task adaptability, ETHOS serves as a *foundation model* for PHT generation.

Once trained, ETHOS can generate multiple simulated future patient health timelines (PHTs) and estimate the probability of clinical events occurring within those trajectories (e.g., ICU admission). For adverse events during an inpatient stay, these probabilities serve as dynamic risk estimates, effectively functioning as an early warning system. Unlike traditional methods that require separate models or task-specific retraining, ETHOS operates as a unified model capable of concurrently assessing multiple clinical endpoints. As new patient data become available, risk estimates are automatically updated. This flexible and scalable risk prediction framework, built on ETHOS, is referred to as the Adaptive Risk Estimation System (ARES), as illustrated in Figure 2. Risk is quantified into five ordinal categories (levels 1 through 5) based on the predicted probability: 0–20% corresponds to level 1, 20–40% to level 2, and so on.

In this paper, we present ARES and introduce a novel explainability framework that delivers fully personalized insights, potentially allowing clinicians to understand the specific factors influencing the system's risk predictions for individual patients. We benchmark the performance of ARES against state-of-the-art methods across multiple clinically relevant tasks, demonstrating its superior predictive accuracy. We validate its effectiveness and provide the accompanying code for the full reproduction of all the experiments by other researchers.

Data Description

In this study, we used the Medical Information Mart for Intensive Care (MIMIC-IV) version 2.2 database [25, 26], including its ED extension. MIMIC-IV, developed by the Massachusetts Institute of Technology and Beth Israel Deaconess Medical Center contains de-identified health records for almost 300,000 patients either admitted to the ED and/or hospital at BIDMC from 2008 to 2019. Detailed patient demographics are presented in Table 2.

Evaluation

Following the tokenization process, the data of 299,721 unique patients from the MIMIC-IV dataset was converted into 285,622 PHTs, which were subsequently used for training and testing. Patients were excluded if they had no usable data after tokenization. This occurs when patients in MIMIC have little or no structured information available, or when the available information (e.g., clinical notes, imaging) is not tokenized in the current ETHOS version. Of the total PHTs, approximately 63% (180,733) contained hospital admissions records. The tokenized dataset comprised over 357 million tokens in total. In the Supplementary Materials, we provide detailed information regarding the MIMIC-IV data used (Table 7), patient demographics (Table 2), characteristics of the PHTs (Table 6) and tokens (Table 11). The model was trained and validated on 90% of the PHTs, with the remaining 10% reserved for testing. During inference, at least $N = 100$ fPHTs were generated for each investigated task.

The predictive performance of ARES and MEDS-Tab was evaluated on three individual clinical endpoints—hospital mortality (HM), ICU admission (IA), and prolonged hospital stay (PS; defined as length of stay >90th percentile)—and, as an illustrative demonstration of joint risk modeling, on a composite criterion combining these events (HM-IA-PS). The prevalence of these tasks is: 1.85%, 15.44%, 9.01% and 20.39%, respectively. This composite endpoint demonstrates ARES's capacity to compute joint probabilities across heterogeneous outcomes and to naturally model their statistical dependencies. The composite score represents the cumulative risk of clinician-defined critical events. All predictions were generated at the hospital admission. As summarized in Figure 3, Figure 5, and Table 1, ARES consistently outperformed MEDS-Tab across both individual and composite endpoints, achieving higher AUC values in every case. Notably, these gains were observed across all racial subgroups, with the most pronounced improvements for Asian and Hispanic patients, indicating ARES's robustness and its potential to reduce disparities in predictive accuracy.

Figure 4 illustrates the dynamic risk trajectories generated by ARES, showcasing how the system continuously updates probability estimates for key clinical outcomes, including ICU admission, prolonged hospital stay, and mortality, as new clinical events occur. The figure highlights specific medical interventions, such as laboratory tests and procedures, that drive significant changes in risk estimates, demonstrating ARES's ability to integrate evolving patient data into real-time risk assessment. The results underscore the model's capacity to capture complex temporal relationships be-

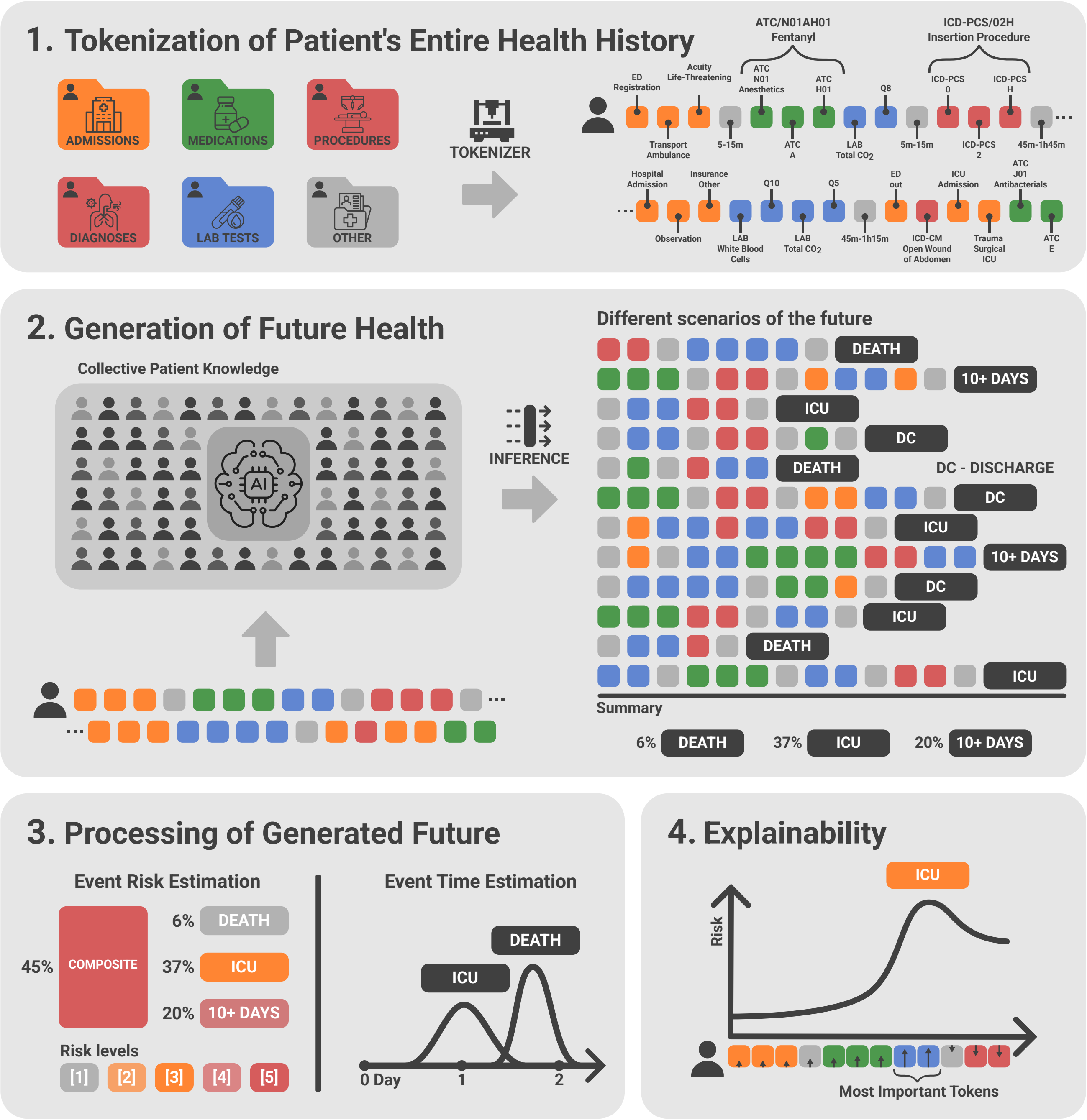

Figure 1. Workflow of the Adaptable Risk Estimation Score (ARES) Framework. This figure illustrates the ARES framework, developed on the ETHOS model, for dynamic and explainable risk evaluation. Panel 1 depicts the tokenization of a patient's entire health history into structured events represented as a sequence of tokens (PHTs), incorporating standardized coding systems such as ATC for medications, ICD-PCS for procedures, and others. Panel 2 demonstrates how the ETHOS model trained on a large dataset of PHTs to simulate potential future patient health timelines (fPHTs). By analyzing a particular patient's known PHT and generating multiple fPHTs, the model estimates the probabilities of critical outcomes, such as inpatient death, ICU admission, or a prolonged hospital stay exceeding 10 days. Panel 3 showcases the result of processing of fPHTs to calculate event-specific risks and predict the timing of these events, should they occur. Risk levels are defined across five categories, color-coded for enhanced clinical interpretability. Panel 4 showcases the explainability module, which identifies the key factors influencing specific risk estimates, offering personalized and actionable insights to support clinical decision-making. In this example, blue tokens indicate factors contributing to an increased risk of ICU admission.

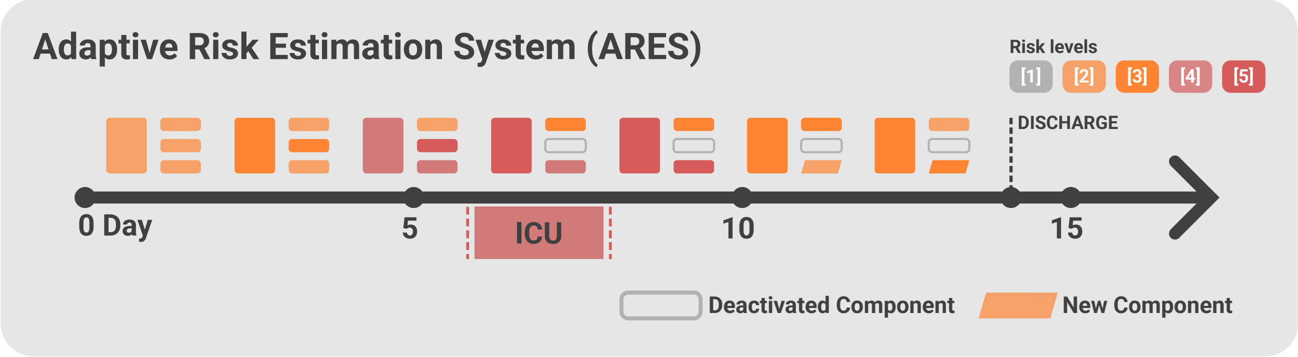

Figure 2. Timeline of a Patient's Hospital Stay and Hypothetical Risk Predictions by ARES. This figure illustrates the timeline of a patient's hospital stay, from admission to discharge around Day 14, demonstrating how ARES dynamically adjusts its predictions based on the patient's evolving clinical status and medical history. By Day 5, ARES predicts a high risk of ICU admission, which is subsequently confirmed as the patient is admitted around Day 6. Once the patient is in the ICU, ARES discontinues ICU risk evaluation, as indicated by the "Deactivated Component" label. After the ICU stay, ARES identifies an increased likelihood of a hospital stay exceeding 10 days. Upon reaching the 10-day threshold, ARES automatically recalibrates its predictions, replacing the previous risk estimation with the likelihood of a 15-day stay, now categorized as a "New Component" in the risk assessment.

tween clinical events, dynamically recalibrating risk scores based on patient status and treatment progression.

In addition to risk which are part of ARES and to contextualize the predictive capabilities of ETHOS, we compared its performance against traditional early warning scores and other ML models. Figure 3 presents the AUC values (ROC curves in Figure 2) for key ED benchmark tasks: hospitalization at triage, critical outcomes within 12 hours of triage, and ED re-presentation within 72 hours post-discharge. ETHOS demonstrated consistently superior predictive accuracy across all evaluated tasks. We provide detailed numerical values in the Supplement (Table 3,4,5).

The risks provided by ETHOS were also found to be well-calibrated, as tested by calibration curves. Brier scores were found in the range 0.01-0.14 depending on the task, indicating excellent to good performances, as shown in Figure 4.

Discussion

The ARES framework introduces an innovative approach to building predictive models by leveraging cutting-edge artificial intelligence technology. Several aspects of this approach distinguish it from traditional models. First, ARES enables dynamic risk estimation at any time during a patient's stay, from admission to discharge. Powered by ETHOS [15], ARES utilizes PHTs and incorporates all available clinical information at the time of risk estimation. Unlike traditional models, which rely on static data points such as information collected within 24 hours after admission or ED presentation or data up to triage [27, 28], ARES continuously adapts to the patient's evolving clinical status. This adaptability overcomes a key limitation of static models, which may not perform optimally outside the narrow time frames for which they are designed. This capability is demonstrated in the accompanying Figure 4 and Table 10, which illustrate how risk evolves over time during a patient's hospital stay. These visualizations, which depict how personalized risk evolves over time to reach the current estimates, provide insights into the specific factors driving model predictions for each patient. They highlight clinical events associated with increased or decreased risk, offering real-time explainability. By identifying the most influential features contributing to an individual's risk assessment, ARES have the potential to empower clinicians with a clearer understanding of the rationale behind each prediction.

As illustrated in Figure 1, ARES can estimate risk for various critical events, such as in-hospital mortality, ICU admission, and prolonged hospital stays. Beyond these standard metrics, additional indicators can be integrated seamlessly, including the risk of ICU admission during a specific length of stay, ICU readmission, acute kidney injury, sepsis, cardiac arrest, or 30-day readmission, and others. The ETHOS model, which underpins ARES, allows for the dynamic combination of these risks into composite measures while accounting for their interdependencies. For example, the occurrence of mortality on day 8 would render the probability of a 10-day hospital stay zero. This ability to incorporate conditional and causal relationships between tracked events is another strength of ARES. Importantly, integrating additional metrics does not require model retraining or modifications of ETHOS. Once a range of possible future PHTs has been generated, any additional metrics can be calculated with minimal computational resources, making ARES scalable and adaptable to diverse healthcare settings.

In its current implementation, ETHOS distills multiple fPHTs into a single predictive decision, such as inpatient mortality. However, this approach overlooks the wealth of longitudinal information contained in these trajectories, including the sequence of clinical events that lead to a particular outcome, or the absence thereof. By merely predicting the likelihood of an adverse event, valuable insights into the pathways that contribute to deterioration or recovery remain underutilized. Expanding ARES to provide a more granular, trajectory-based interpretation of risk would allow clinicians not only to assess a patient's probability of experiencing a critical event but also to understand the evolving clinical course leading to that outcome including the cost. This enhanced approach would address a key limitation highlighted in the early warning paradox [29], where models trained on retrospective data may fail to capture the full complexity of clinical interventions and their effects on patient outcomes. Moving forward, we aim to refine ARES to incorporate and visualize these probabilistic trajectories. This will equip clinicians with deeper, more actionable insights into clinical risk dynamics and potentially provide new information about causality in patient outcomes.

We recognize that, to date, ARES has not undergone formal usability testing with frontline clinicians, yet their ultimate impact depends on seamless integration into real-world workflows. Emergency medicine specialists on our team have provided informal feedback on feasibility and clarity of the risk estimates and explanatory highlights, and we are now designing pilot simulations in which physicians will "round" on de-identified patient cases presented through mock electronic charts powered by ARES. These studies, first leveraging MIMIC-derived timelines and subsequently our own Mass General Brigham data, will allow us to observe decision points, gather qualitative feedback on timing and interpretability of alerts, and refine both the user interface and explanation formats. We anticipate that iterative, case-based testing will guide the development of a clinician-centered dashboard, ensuring that ARES's predictions align with care priorities and support

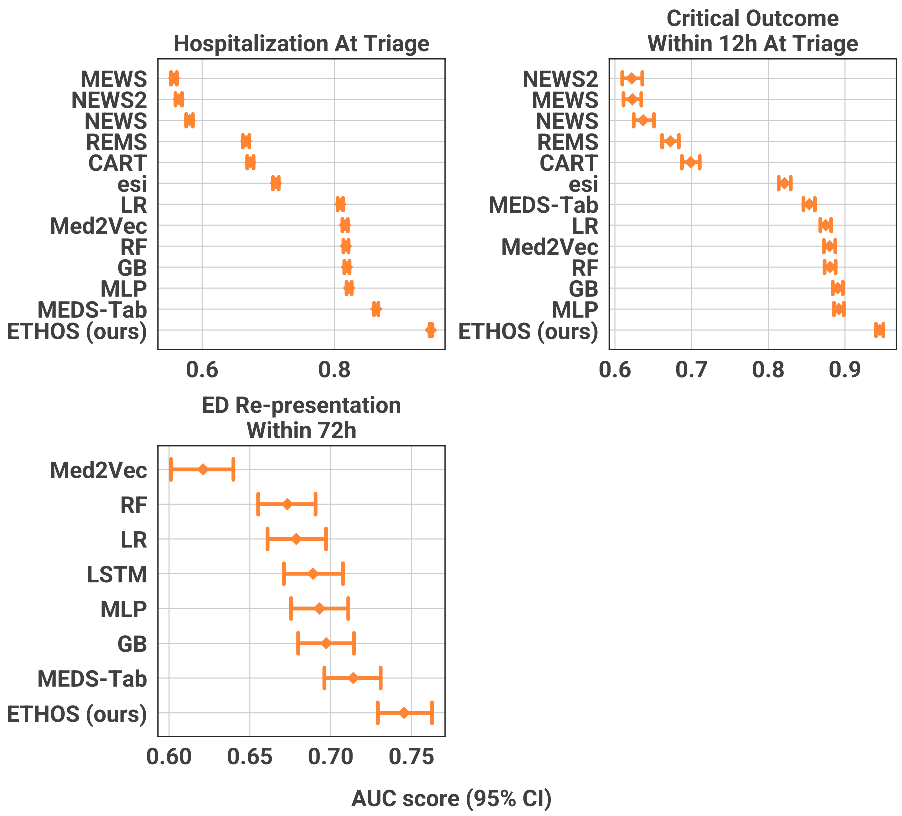

Figure 3. Predictive results for the ED benchmark tasks. Fewer methods appear in the ED re-presentation task (right) because score-based approaches, designed specifically to estimate in-hospital deterioration, are not applicable once the patient has left the ED. Ethos consistently achieves the best performance across all evaluated tasks.

timely, actionable insights in the emergency setting.

This study has several important limitations. First, although we demonstrated ETHOS using PHTs derived from the MIMIC-IV-ED dataset, its performance on data from other institutions may be compromised without retraining on external cohorts. Electronic health record systems and clinical workflows differ substantially across hospitals—driven by variations in documentation practices, patient case-mix, and care protocols—so models trained on one site can yield misleading risk estimates when deployed elsewhere. Moreover, our training data may harbor demographic and institutional biases (for example, overrepresentation of certain age, race, or socioeconomic groups), which could impair generalizability and exacerbate health inequities if unaddressed. The MIMIC dataset is relatively small, and although it contains dense, diverse information, the limited cohort size inevitably constrains generalization. Our model is explicitly designed to scale, and we expect its performance to further improve when trained on larger and more diverse datasets that capture a broader range of clinical variability. We have not yet conducted a thorough fairness audit to quantify potential disparities in ETHOS's predictions across sex, race, or ethnicity. By contrast, in domains such as radiology or pathology, data inputs like images are relatively standardized, enabling easier cross-institution transfer. To facilitate broader validation and retraining, we have ensured that the ETHOS-ARES codebase is fully compatible with the MEDS health AI data standard [30]. This interoperability simplifies the process for other researchers to apply identical model architectures to their local data, perform bias and subgroup analyses, and iteratively refine ETHOS for diverse patient populations.

We also recognize that our evaluation on the extensively curated MIMIC-IV dataset may underestimate challenges encountered in real-world EHRs, which often exhibit higher rates of missing or irregular data, temporal shifts in documentation and care processes, and evolving patient populations. Although ETHOS is designed to operate on incomplete timelines, elevated missingness will still impair model accuracy. It is unclear if temporal biases or practice changes may introduce drift over time for ETHOS because the inferences are based on the context which contemporary to predictions, but this has not been investigated yet. Future work will systematically assess ETHOS's resilience to these factors and develop strategies for ongoing recalibration in heterogeneous clinical environments. In addition, our current benchmarking employed only classical risk-prediction methods. While sufficient to demonstrate ETHOS's competitiveness on established tasks, more sophisticated approaches could yield higher benchmark scores. Even if such models were to match or slightly exceed our performance on static benchmarks, this would not diminish ETHOS's primary contribution: a dynamic, explainable framework that adapts predictions in real time as new clinical data become available. Future work will expand benchmarking to include a broader set of advanced methods (e.g. [22]).

In current implementation, we exclude unstructured clinical text and because of that ETHOS may miss nuanced patient information, such as narrative impressions or social determinants, that could enhance risk estimation and zero-shot generalizability. Integrating free-text notes poses challenges in segmenting and embedding variable-length narratives alongside structured events without overwhelming the model's capacity. In future work, we will explore the use of pretrained clinical-language-model embeddings, hierarchical chunking of note content, and multimodal fusion techniques to incorporate these rich data into the PHTs.

Data standardization is often proposed as a solution to address the challenges of variability in healthcare data. However, achieving meaningful standardization would require identifying commonalities between healthcare systems, an endeavor that may not be feasible given the diversity of clinical practices, patient populations, and institutional workflows. An alternative is to train AI models, such as ETHOS, on raw data from diverse institutions, allowing

the model itself to learn and interpret the underlying patterns and clinical pathways. This approach mirrors the capability of large language models to discern meaning from vastly different styles of text and presentations or even different languages, leveraging the same transformer architecture as ETHOS. We performed an energy consumption analysis comparing training of ETHOS to other known LLMs that can be seen in Table 9.

In summary, recent advances in AI have created unprecedented opportunities for innovative solutions like ARES, which harness large volumes of heterogeneous data to build general-purpose models whose predictive performance exceeds state-of-the-art methods. ARES delivers dynamic, personalized risk estimates and offers real-time explainability, empowering clinicians to make better-informed decisions. Moreover, its modular architecture and the underlying ETHOS framework enable seamless integration of additional data modalities, such as radiology, genomics, and other institutional datasets, further enhancing predictive accuracy and broadening applicability across diverse healthcare environments. Although our results are promising, the clinical impact remains uncertain. Demonstrating ARES's true utility in real-world settings will be the focus of our future work.

As healthcare costs and complexity continue to rise, PHT-based frameworks like ARES show a promising pathway towards data-driven AI-enabled individualized patient care with the potential to reduce morbidity, improve outcomes, and lower healthcare costs.

Potential implications

The Adaptive Risk Estimation System (ARES) offers several near-term opportunities to enhance clinical care beyond emergency early warning. First, by continuously updating personalized risk profiles, ARES can inform dynamic triage and resource allocation decisions across hospital units. For example, bed managers could use the ARES scores to anticipate ICU demand several hours in advance, improving staff deployment and reducing admission delays.

Second, ARES's real-time explainability module—highlighting the specific tokens driving risk changes—can support shared decision-making at the bedside. Clinicians may review the key factors that elevated a patient's risk, facilitating targeted interventions such as order adjustments, specialist consults, or heightened monitoring, and enabling more transparent discussions with patients and families.

Third, ARES can serve as a decision-support tool in clinical research and quality improvement initiatives. Embedded within institutional dashboards, ARES could identify patient subgroups with unexpectedly high or low risk trajectories, prompting retrospective chart reviews or prospective studies to refine care pathways. Its use in pilot implementation studies may reveal workflow integrations that optimize alert timing and reduce alarm fatigue.

Methods

ETHOS and Probabilistic Inference

We introduced ETHOS in [15]. It operates on Patient Health Timelines (PHTs), which are tokenized chronological representations of patient medical histories (see Table 8). Here, tokenization refers to encoding clinical events, such as inpatient visits, procedures, laboratory results, medication administrations, and vital signs, as sequences of discrete tokens. The intervals between events are captured using specialized time-interval tokens. Formally, a PHT is a sequence of integer labels corresponding to these tokens, and its length can reach hundreds of thousands of tokens.

ETHOS employs a transformer-based generative model to predict future clinical events from tokenized PHTs. During inference, ETHOS generates successive tokens, each denoting a prospective future event, until a predefined stopping condition is met, such as the appearance of a target event token or the attainment of a simulation time limit. By repeatedly simulating multiple future PHTs (fPHTs) for each patient, ETHOS explores a range of possible trajectories, thereby quantifying the inherent uncertainty in its predictions. For instance, if N fPHTs are simulated and M of these trajectories include an inpatient mortality token, the estimated mortality probability is given by M/N (see section). All probabilistic inferences in this paper utilize Monte Carlo (MC) sampling with $N = 100$ simulated fPHTs per patient, which inevitably introduces variability due to finite draws. We quantify this uncertainty by modeling the number of positive outcomes as a $Binomial(N, p)$ random variable and computing 95% confidence intervals, and visualizing these as shaded bands around the mean risk trajectory (e.g., Figure 4).

For detailed information on the transformer architecture, PHT statistics, and tokenization procedures, as well as intuitive explanation of ETHOS, please refer to our first publication [15] and section .

Data Preprocessing

We extracted relevant data from the MIMIC-IV tables as detailed in Table 7. Laboratory tests and medications were standardized using ATC codes, and all diagnostic and procedural codes were mapped to ICD-10 when necessary, as described in [15]. Additional tables requiring advanced processing, such as clinical notes, were not included in the current implementation of ETHOS.

The dataset was split into two disjoint groups: training/validation (90%) and testing (10%). Exactly the same splits were used for all methods investigated.

Tokenization, PHT Construction, model training

The core of ETHOS lies in constructing PHTs from electronic medical records (EMRs) using a tokenization strategy that captures diverse clinical events. A PHT represents a patient's medical history as a sequence of tokens, each encoding specific health-related information organized chronologically. This structured representation enables comprehensive modeling of patient journeys and more accurate clinical predictions. To build PHTs, we used the MEDS-DEV [31] extraction pipeline that converts EHR data to an intermediate format called MEDS [30] to facilitate further data transformations. Advanced transformation operations were subsequently applied, breaking down each event into 1 to 7 tokens based on its complexity.

For example, lab test results were encoded using quantile-based tokens to represent clinical significance. Time-interval tokens were added to mark the elapsed time between successive events, with intervals shorter than 5 minutes omitted and longer gaps tokenized into 19 distinct interval tokens. Continuous numerical values, such as lab test results, were similarly quantile-encoded using ten quantiles, balancing clinical interpretability and predictive precision. Diagnostic and procedural codes, including ICD-10-CM, ICD-10-PCS, and ATC drug codes, were encoded hierarchically, which leveraged their inherent structure to enhance the transformer model's attention mechanisms. For more details, refer to [15].

Static patient attributes such as gender, marital status, race, and body mass index (BMI) were encoded using a single token depending on the value. For age, tokens of quantiles were reused, allowing age representation from 0 to 99. For instance, a 46-year-old patient would be coded as Q5 and Q7. Attributes with potential variability were represented using their most recently known value at the start of the timeline. By incorporating these elements, ETHOS ensured a richer and more adaptable representation of patient timelines.

During the training phase, 6 million tokens (1.8% of the train/validation dataset) were used for validation to balance model optimization and computational efficiency. The detailed statistics

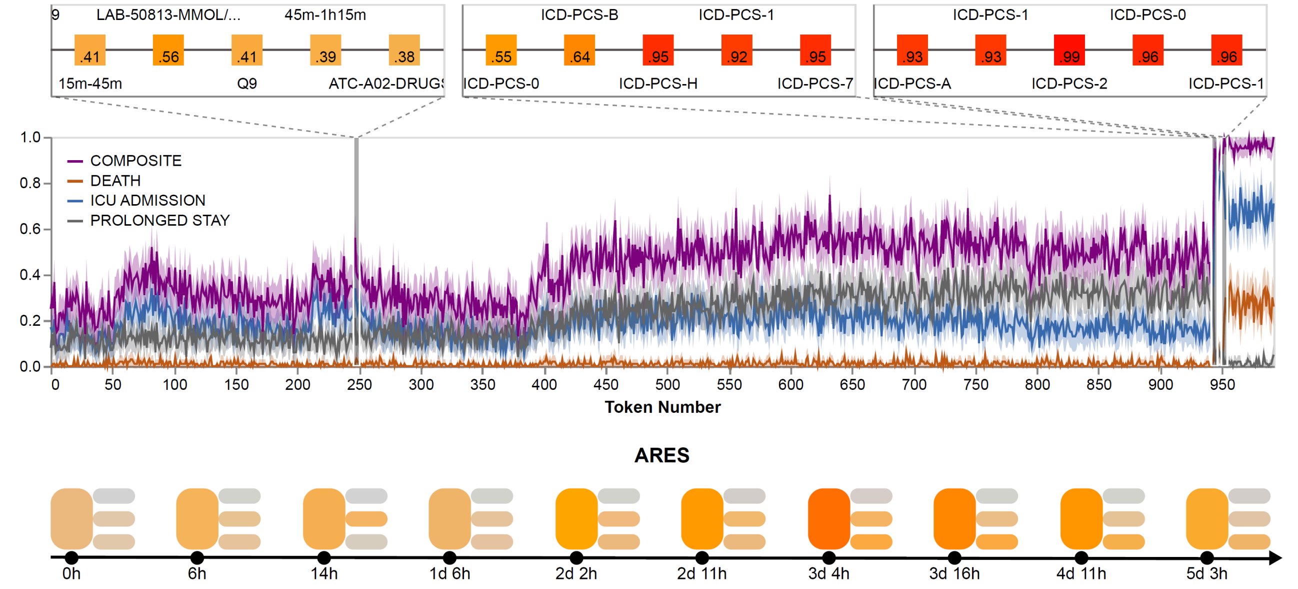

Figure 4. ARES Risk Trajectories. This figure illustrates risk trajectories for nearly 1000 tokens preceding patient death, as monitored by ARES, which evaluates the probability of death, ICU admission, prolonged hospital stay, and a composite risk score. The lower panel provides a color-coded representation of risk with the actual time since the ED presentation. In contrast, the upper panel highlights three 5-token regions influencing risk predictions at areas marked by the thin gray bar. In the first region, token LAB-50813 (Lactate Blood Test) increases the composite risk score from 0.41 to 0.56, but since the result falls in Q9 (80–90th percentile), ETHOS downgrades the risk estimate back to the previous level. In the second region (close to the end), a sharp increase in composite risk occurs due to heightened ICU admission triggered by ICD-PCS code 0BH17EZ, which is coded by 7 tokens (only 5 visible), which represents Endotracheal Airway Insertion into the Trachea via Natural or Artificial Opening. The 'H' token specifically signals ETHOS to escalate the ICU risk to nearly 1.0, indicating that the patient is being intubated de novo. The ICD-10-PCS breakdown confirms the procedure as a respiratory intervention involving tracheal insertion via a natural or artificial opening. ICD-PCS 0BH17EZ does not increase the risk of death, but the next ICD-PCS 5A12012 (5 tokens coding A1202 visible) raises the risk of death to about 0.25. We note that an increased risk of death is associated with a decreased risk of ICU admission, as these are competing risks. This visualization demonstrates how ARES dynamically adjusts risk scores based on evolving patient data, integrating clinical trajectories into real-time risk assessment. In this example, the rapid risk increases immediately following invasive procedures (e.g., intubation) should be interpreted as retrospective severity markers rather than actionable alerts, since they occur too late to guide effective intervention. Shaded bands around each trajectory denote the 95% confidence intervals arising from Monte Carlo sampling.

about the tokenized dataset are available in Table 6, 11, and information about the model is in Figure 1.

Explainability

As illustrated in Figure 4, stochastic simulations can be initiated not only from the most recent token representing current information but also from any preceding token in the patient's history. This allows risk estimates to be visualized as a time series, highlighting how specific medical events affect risk over time. This approach provides intuitive visualizations, offering clinicians clear insights into the factors contributing to current risk values.

Methods used for benchmarking

We followed benchmarking tasks for emergency department models presented in the Emergency Department MIMIC-IV-ED benchmark paper [27]. Three tasks were defined: prediction of the hospital admission at triage, prediction of the critical outcome (death or transfer to ICU within 12 hours) at triage, and ED re-presentation within 72 hours after discharge from ED. We applied machine learning methods (logistic regression, random forest, gradient boosting), scoring systems MEWS [13], NEWS [12, 32, 33], Rapid Emergency Medicine Scores (REMS) [34], Cardiac Arrest Risk Triage (CART) [35], five-level triage system Emergency Severity Index (ESI) [36] and neural networks-based models including multilayer perceptron, Med2Vec [37] and Long Short-Term Memory (LSTM) [38].

To compare tasks used for early warning scores, we compared the MEDS-Tab library [39] which was used to establish a baseline. MEDS-Tab converts time-series EHR data into a tabular format by aggregating features across multiple time windows. It takes longitudinal patient data and applies various aggregation functions (like sum, count, min, max) over different historical window sizes to create fixed-size feature vectors, where each feature represents a combination of a medical code, time window, and aggregation method. XGBoost [40] models are trained on these tabular features computed from data windows prior to each prediction time point for each clinical task.

Statistical Methods

The performance of predictive models was evaluated using Receiver Operating Characteristic (ROC) curves and corresponding Area Under the Curve (AUC) values. Bootstrapping techniques were employed to estimate 95% confidence intervals (CIs) for AUCs. Model predicted probabilities were compared with observed event frequencies using calibration curves to evaluate ETHOS's reliability and alignment with real-world clinical outcomes. All statistical analyses were conducted using Python-based libraries, including scipy and scikit-learn [41, 42]. Data visualization, including ROC curves, calibration plots, and other statistical figures, was performed using matplotlib, seaborn and altair.

Availability of source code and requirements

- Project name: ETHOS-ARES
- Project home page: https://github.com/ipolharvard/ethos-ares
- Operating system(s): Platform independent
- Programming language: Python
- Other requirements: Polars, Pytorch etc. (see pyproject.toml)

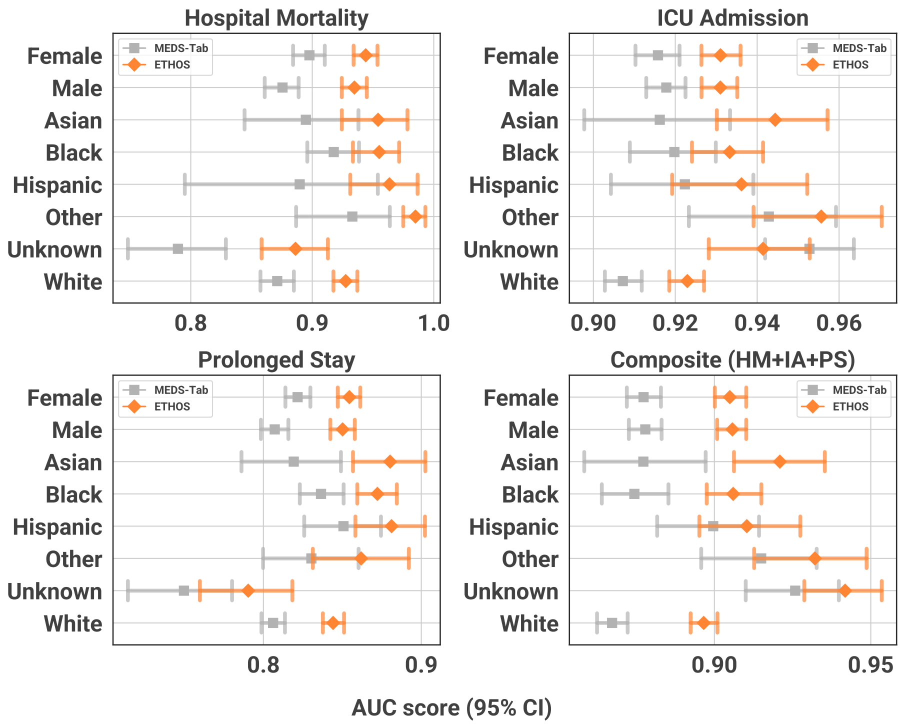

Figure 5. AUC Comparison Between ETHOS and MEDS-Tab Across Demographic Subgroups and Prediction Tasks. AUC scores with 95% confidence intervals are shown for ETHOS (orange) and MEDS-Tab (gray) across four prediction tasks: Hospital Mortality, ICU Admission, Prolonged Stay, and Composite Outcome (Hospital Mortality + ICU Admission + Prolonged Stay). Performance is reported for the overall population and stratified by gender (Female, Male) and race (Asian, Black, Hispanic, Other, Unknown, White). ETHOS consistently outperforms MEDS-Tab across all demographic subgroups and tasks.

- License: MIT

Data availability

The MIMIC-IV dataset is publicly available at `https://physionet.org/content/mimiciv/2.2`. Its Emergency Department extension is available at `https://physionet.org/content/mimic-iv-ed/2.2/`. These are controlled-access datasets; users need to sign in to PhysioNet, apply for a credentialed account, sign a Data Use Agreement, and follow training on human research data. Annotations to the code are available at ML-DOME [43].

Declarations

List of abbreviations

- **AI** – Artificial Intelligence
- **ARES** – Adaptive Risk Estimation System
- **ATC** – Anatomical Therapeutic Chemical (codes)
- **AUC** – Area Under the Curve
- **BIDMC** – Beth Israel Deaconess Medical Center
- **BMI** – Body Mass Index
- **CART** – Cardiac Arrest Risk Triage
- **CI** – Confidence Interval
- **ED** – Emergency Department
- **EHR** – Electronic Health Record
- **EMR** – Electronic Medical Record
- **ESI** – Emergency Severity Index
- **ETHOS** – Enhanced Transformer for Health Outcome Simulation
- **fPHT** – Future Patient Health Timeline
- **GDP** – Gross Domestic Product
- **ICD-10** – International Classification of Diseases, 10th Revision
- **ICU** – Intensive Care Unit
- **LLM** – Large Language Model
- **LSTM** – Long Short-Term Memory
- **MEDS** – Medical Event Data Standard
- **MEWS** – Modified Early Warning Score
- **MIMIC-IV** – Medical Information Mart for Intensive Care, version IV
- **ML** – Machine Learning
- **NEWS** – National Early Warning Score
- **PHT** – Patient Health Timeline
- **REMS** – Rapid Emergency Medicine Score
- **ROC** – Receiver Operating Characteristic
- **XGBoost** – eXtreme Gradient Boosting

Consent for publication

Not applicable

Competing Interests

Dr. Jia is also affiliated with Verily Life Science. Dr. Cunningham reports consultancy for Edgewise Therapeutics, Occlutech, and us2.ai. Dr. Bates reports grants and personal fees from EarlySense, personal fees from CDI Negev, equity from ValeraHealth, equity from Clew, equity from MDClone, personal fees and equity from AESOP, personal fees and equity from FeelBetter, personal fees and equity from Guided Clinical Solutions, outside the submitted work. Other authors declare no competing interests.

Funding

Dr. Sitek reports grant support NIH R01HL159183, and Dr. Cunningham reports grant support from the American Heart Association (23CDA1052151) and the National Heart, Lung, and Blood Institute (1K23HL168163).

Author's Contributions

- **PR** – Conceptualization, Methodology, Formal Analysis(lead), Visualization, Software(lead), Writing—Review, and Editing.
- **MKG** – Conceptualization, Methodology, Formal Analysis, Writing—Review, Editing, and Visualization.
- **NO** – Writing—Review, Formal Analysis.
- **DG** – Writing–Review, and Editing.
- **YJ** – Methodology, Writing–Review, and Editing.
- **SB** – Conceptualization.
- **MBAM** – Methodology, Writing–Review, and Editing.
- **JW** – Writing–Review, and Editing.
- **AES** – Writing–Review, and Editing.
- **JWC** – Writing–Review, and Editing.
- **DWB** – Writing–Review, and Editing.
- **AS** – Conceptualization(lead), Methodology, Writing–Original Draft, Preparation, and Supervision.

Acknowledgements

We thank Kinga Renc, M.Arch, for her invaluable assistance with graphic design. We also thank Ethan Steinberg for the careful and very helpful review of our codebase.

References

1. Centers for Medicare & Medicaid Services, National Health Expenditure Data: Historical; 2024. Accessed: 2025-05-03. https://www.cms.gov/data-research/statistics-trends-and-reports/national-health-expenditure-data/historical.
2. Gunja MZ, Gumas ED, Williams RD II, U.S. Health Care from a Global Perspective, 2022: Accelerating Spending, Worsening Outcomes; 2023. Accessed: 2025-1-26. https://www.commonwealthfund.org/publications/issue-briefs/2023/jan/us-health-care-global-perspective-2022.
3. Committee on the Future of Emergency Care in the United States Health System . Hospital-based emergency care: at the breaking point. Washington, D.C., DC: National Academies Press; 2007.
4. Yang KK, Lam SSW, Low JMW, Ong MEH. Managing emergency department crowding through improved triaging and resource allocation. Oper Res Health Care 2016 Sep;10:13–22.
5. Horton DJ, Graves KK, Kukhareva PV, Johnson SA, Cedillo M, Sanford M, et al. Modified early warning score-based clinical decision support: cost impact and clinical outcomes in sepsis. JAMIA Open 2020 Jul;3(2):261–268.
6. Adams R, Henry KE, Sridharan A, Soleimani H, Zhan A, Rawat N, et al. Prospective, multi-site study of patient outcomes after implementation of the TREWS machine learning-based early warning system for sepsis. Nat Med 2022 Jul;28(7):1455–1460.
7. Edelson DP, Churpek MM, Carey KA, Lin Z, Huang C, Siner JM, et al. Early warning scores with and without artificial intelligence. JAMA Netw Open 2024 Oct;7(10):e2438986.
8. Gerry S, Bonnici T, Birks J, Kirtley S, Virdee PS, Watkinson PJ, et al. Early warning scores for detecting deterioration in adult hospital patients: systematic review and critical appraisal of methodology. BMJ 2020 May;369:m1501.
9. Winslow CJ, Edelson DP, Churpek MM, Taneja M, Shah NS, Datta A, et al. The impact of a machine learning early warning score on hospital mortality: A multicenter clinical intervention trial. Crit Care Med 2022 Sep;50(9):1339–1347.
10. Escobar GJ, Liu VX, Schuler A, Lawson B, Greene JD, Kipnis P. Automated identification of adults at risk for in-hospital clinical deterioration. N Engl J Med 2020 Nov;383(20):1951–1960.
11. Cummings BC, Blackmer JM, Motyka JR, Farzaneh N, Cao L, Bisco EL, et al. External validation and comparison of a general ward deterioration index between diversely different health systems. Crit Care Med 2023 Jun;51(6):775–786.
12. Williams B. The National Early Warning Score: from concept to NHS implementation. Clin Med 2022 Nov;22(6):499–505.
13. Subbe CP, Kruger M, Rutherford P, Gemmel L. Validation of a modified Early Warning Score in medical admissions. QJM 2001 Oct;94(10):521–526.
14. Vaswani A, Shazeer N, Parmar N, Uszkoreit J, Jones L, Gomez AN, et al. Attention is all you need. Adv Neural Inf Process Syst 2017;30.
15. Renc P, Jia Y, Samir AE, Was J, Li Q, Bates DW, et al. Zero shot health trajectory prediction using transformer. NPJ Digit Med 2024 Sep;7(1):256.
16. Yang Z, Mitra A, Liu W, Berlowitz D, Yu H. TransformEHR: transformer-based encoder-decoder generative model to enhance prediction of disease outcomes using electronic health records. Nat Commun 2023 Nov;14(1):7857.
17. Li Y, Mamouei M, Salimi-Khorshidi G, Rao S, Hassaine A, Canoy D, et al. Hi-BEHRT: Hierarchical Transformer-Based Model for Accurate Prediction of Clinical Events Using Multimodal Longitudinal Electronic Health Records. IEEE J Biomed Health Inform 2023 Feb;27(2):1106–1117.
18. Luo X, Rechardt A, Sun G, Nejad KK, Yáñez F, Yilmaz B, et al. Large language models surpass human experts in predicting neuroscience results. Nat Hum Behav 2024 Nov;.
19. Thirunavukarasu AJ, Ting DSJ, Elangovan K, Gutierrez L, Tan TF, Ting DSW. Large language models in medicine. Nat Med 2023 Aug;29(8):1930–1940.
20. Kraljevic Z, Bean D, Shek A, Bendayan R, Hemingway H, Yeung JA, et al. Foresight-a generative pretrained transformer for modelling of patient timelines using electronic health records: a retrospective modelling study. Lancet Digit Health 2024 Apr;6(4):e281–e290.
21. McDermott MBA, Nestor BA, Argaw P, Kohane I. Event Stream GPT: A data pre-processing and modeling library for generative, pre-trained transformers over continuous-time sequences of complex events. Adv Neural Inf Process Syst 2023 Jun;abs/2306.11547.
22. Steinberg E, Fries J, Xu Y, Shah N. MOTOR: A time-to-event foundation model for structured medical records. arXiv [csLG] 2023 Jan;.
23. Li Y, Rao S, Solares JRA, Hassaine A, Ramakrishnan R, Canoy D, et al. BEHRT: Transformer for electronic health records. Sci Rep 2020 Apr;10(1):7155.
24. Jeong H, Oufattole N, Mcdermott M, Balagopalan A, Jangeesingh B, Ghassemi M, et al. Event-Based Contrastive Learning for medical time series. arXiv [csLG] 2023 Dec;.
25. Johnson AEW, Bulgarelli L, Shen L, Gayles A, Shammout A, Horng S, et al. MIMIC-IV, a freely accessible electronic health record dataset. Sci Data 2023 Jan;10(1):1.
26. Johnson A, Bulgarelli L, Pollard T, Horng S, Celi LA, Mark R. Mimic-iv. PhysioNet Available online at: https://physionet org/content/mimiciv/22/(accessed Oct 1, 2023) 2023;.
27. Xie F, Zhou J, Lee JW, Tan M, Li S, Rajnthern LS, et al. Benchmarking emergency department prediction models with machine learning and public electronic health records. Sci Data 2022 Oct;9(1):658.
28. Meng C, Trinh L, Xu N, Enouen J, Liu Y. Interpretability and fairness evaluation of deep learning models on MIMIC-IV dataset. Sci Rep 2022 May;12(1):7166.
29. Logan Ellis H, Palmer E, Teo JT, Whyte M, Rockwood K, Ibrahim Z. The early warning paradox. NPJ Digit Med 2025 Feb;8(1):81.
30. Arnrich B, Choi E, Fries JA, McDermott MBA, Oh J, Pollard T, et al. Medical Event Data Standard (MEDS): Facilitating

Machine Learning for Health. In: ICLR 2024 Workshop on Learning from Time Series For Health; 2024. .
31. MEDS-DEV: Establishing Reproducibility and Comparability in Health AI;. https://github.com/mmcdermott/MEDS-DEV.
32. Smith GB, Prytherch DR, Meredith P, Schmidt PE, Featherstone PI. The ability of the National Early Warning Score (NEWS) to discriminate patients at risk of early cardiac arrest, unanticipated intensive care unit admission, and death. Resuscitation 2013 Apr;84(4):465–470.
33. Zhang S, Xu Y, Usuyama N, Xu H, Bagga J, Tinn R, et al. A multimodal biomedical foundation model trained from fifteen million image–text pairs. NEJM AI 2025 Jan;2(1).
34. Olsson T, Terent A, Lind L. Rapid Emergency Medicine score: a new prognostic tool for in-hospital mortality in nonsurgical emergency department patients. J Intern Med 2004 May;255(5):579–587.
35. Churpek MM, Yuen TC, Park SY, Meltzer DO, Hall JB, Edelson DP. Derivation of a cardiac arrest prediction model using ward vital signs. Crit Care Med 2012 Jul;40(7):2102–2108.
36. Eitel DR, Travers DA, Rosenau AM, Gilboy N, Wuerz RC. The Emergency Severity Index triage algorithm version 2 is reliable and valid. Acad Emerg Med 2003 Oct;10(10):1070–1080.
37. Choi E, Bahadori MT, Searles E, Coffey C, Thompson M, Bost J, et al. Multi-layer representation learning for medical concepts. In: Proceedings of the 22nd ACM SIGKDD International Conference on Knowledge Discovery and Data Mining New York, NY, USA: ACM; 2016. .
38. Hochreiter S, Schmidhuber J. Long short-term memory. Neural Comput 1997 Nov;9(8):1735–1780.
39. Oufattole N, Bergamaschi T, Kolo A, Jeong H, Gaggin H, Stultz CM, et al. MEDS-Tab: Automated tabularization and baseline methods for MEDS datasets. arXiv [csLG] 2024 Oct;.
40. Chen T, Guestrin C. XGBoost: A Scalable Tree Boosting System. In: Proceedings of the 22nd ACM SIGKDD International Conference on Knowledge Discovery and Data Mining KDD ’16, New York, NY, USA: Association for Computing Machinery; 2016. p. 785–794.
41. Virtanen P, Gommers R, Oliphant TE, Haberland M, Reddy T, Cournapeau D, et al. SciPy 1.0: fundamental algorithms for scientific computing in Python. Nat Methods 2020 Mar;17(3):261–272.
42. Pedregosa F, Varoquaux G, Gramfort A, Michel V, Thirion B, Grisel O, et al. Scikit-learn: Machine learning in python journal of machine learning research. Journal of machine learning research 2011;12:2825–2830.
43. Renc P, Grzeszczyk MK, Oufattole N, Goode D, Jia Y, Bieganski S, et al., Foundation Model of Electronic Medical Records for Adaptive Risk Estimation. [DOME-ML Annotations]; 2025. https://registry.dome-ml.org/review/jdqxjqrnsb. DOME-ML Registry.
44. Xie F, Chakraborty B, Ong MEH, Goldstein BA, Liu N, et al. AutoScore: a machine learning–based automatic clinical score generator and its application to mortality prediction using electronic health records. JMIR medical informatics 2020;8(10):e21798.

Click here to access/download
Supplementary Material
ETHOS_ARES___GigaScience___Resubmission-part-2.pdf

Personal Cover

Click here to access/download;Personal Cover;Cover_letter_gigascience_resubmission_2.docx.pdf

MASSACHUSETTS GENERAL HOSPITAL

HARVARD MEDICAL SCHOOL

Radiology Department,
Massachusetts General Hospital, Harvard Medical School
55 Fruit St, Boston, MA 02114 Suite 427
asitek@@mgh.harvard.edu

Arkadiusz Sitek, PhD
Associate Investigator
Massachusetts General Hospital
Member of Faculty
Harvard Medical School

Boston, August 5, 2025

Prof. Scott Edmunds, PhD
Editor-in-Chief
GigaScience Press,
BGI Hong Kong, Hong Kong

Dear Prof. Scott Edmunds,

We appreciate the opportunity to submit our revised manuscript and to address the reviewers' remaining concerns. We are grateful to Reviewer #1 for their constructive feedback and for assisting with the follow-up review in the absence of Reviewer #4.

In this revision, we have:

1. Clarified the exclusion criteria for patients removed during tokenization and discussed the representativeness of excluded patients to address potential selection bias.

2. Expanded the discussion on dataset size and generalization, acknowledging that our current dataset is smaller than those used to train large-scale foundation models such as MedBERT, and explaining our plans to scale ETHOS to larger and more diverse datasets.

3. Elaborated on benchmarking limitations, noting that our current evaluation used classical risk-prediction methods and that future work will compare ETHOS to additional advanced models, including MedBERT and ClinicalBERT.

We believe these revisions improve transparency, strengthen the limitations discussion, and address the reviewers' key points without altering the central contributions of our work.

We thank you and the reviewers for your thoughtful guidance throughout the review process, and we look forward to your consideration of this resubmission.

Sincerely,

Arkadiusz Sitek

Arkadiusz Sitek, Ph.D.
Associate Investigator, Massachusetts General Hospital
Member of Faculty, Harvard Medical School
on behalf of all co-authors
