# Supplementary material for: Foundation model of electronic medical records for adaptive risk estimation
Source: Gigascience. 2025 Sep 30;14:giaf107. doi: 10.1093/gigascience/giaf107 (PMC12482913; doi:10.1093/gigascience/giaf107)
Supplement: giaf107_Authors_Response_To_Reviewer_Comments_Revision_1 [file giaf107_authors_response_to_reviewer_comments_revision_1.pdf]

Author's Response To Reviewer Comments

Authors comments:
We thank the reviewer #1 for their comments and help with clarifications on our responses to reviewer #4.

Reviewer reports:

Reviewer #1: thank you for your satisfactory response to our comments.
The only point that remains unclear is the question about the loss of a significant number of files. The authors explain this selection by the tokenization process, which is a satisfactory answer. But they don't discuss at all who are these patients excluded from the analysis? Are they representative of the other patients, or is there a selection bias that could undermine the generalizability of the results?
this point should be discussed in the article
Otherwise, no comments.

Authors' response:

Patients were excluded if no information was available beyond the patient ID. After tokenization, any patient whose sequence contained no tokens between static tokens and timeline_end was excluded. This is because some patients in MIMIC have little or no usable information. Even if a patient has certain data, if that data type is not tokenized in the current version of ETHOS, the resulting tokenized timeline will have a length of zero, leading to exclusion. We explicitly discuss this in Evaluation (Page 2).

Reviewer reports:

Reviewer #4: Unfortunately reviewer #4 is not able to re-review the manuscript. Reviewer #1 kindly helps to look at these additional comments and responses as requested by the editor.

- On the foundation model aspect (questions 2 and 6): the reviewer's question is very pertinent and the answer rather inadequate. A foundation model is a model trained on a very large number of cases, enabling results to be generalized to other cases without re-training. The example given by the reviewer is trained on 28 M patients, and that of the article on 300 k patients, which is much smaller and suggests a lack of generalization. The answer given argues that the granularity of the variables is much greater in the article's model and that the data set is therefore larger, with consequently more parameters on the model than for a foundation model as indicated by the reviewer. What doesn't fit is that in the article's dataset, the variables belonging to the same patient are not independent... so it can't be argued that it's equivalent for training a model to have 1000 variables for each of the 300k patients and 10 variables for each of the 28M patients. (this is what the author maintains).

Authors' response:

We fully agree that a model trained on 300,000 patients cannot match the generalization potential of a model trained on 28 million patients, such as MedBERT. This limitation, which we acknowledge in the Discussion section, reflects the current scale of our dataset rather than an inherent limitation of our

proposed method. Our approach is explicitly designed to scale, and we intend to incorporate larger and more diverse datasets in future work to enhance generalizability.
In our initial response, we sought to clarify a separate point raised by Reviewer #4, who suggested that our model's parameter count is "relatively small," which was not true, and implied that the smaller patient cohort undermines its relevance compared to existing large-scale models. Our intention was not to equate 300k patients with 28M, but to explain that the complexity and richness of our input representation results in a model with higher representational capacity than might be expected purely based on patient count. Unlike models trained on sparse, flat representations, ours leverages dense, longitudinal sequences with fine-grained granularity, contributing to a higher parameter count and learning capacity. This was the purpose of our response to rev #4.
However, we acknowledge the reviewer's correct observation: while each patient in our dataset contributes multiple variables, these are not independent and do not fully compensate for the much larger cohort size in terms of generalization. This distinction is critical, and we thank the reviewer for pointing it out. We clarify in limitations in the discussion (Page 5) that our implementation has limitations of a relatively small size dataset which may prevent generalisation.

Reviewer reports:

- About benchmarks (question 3): we are not familiar with the MedBert and clinicalBert models. The authors have made no effort to convert the dataset to be compatible with these models. It's true that this is a pity, as they are closer to the article's model than a logistic regression. Nevertheless, we don't know whether this adaptation represents a lot of work time, as the authors say in their answer, or whether it can be done in a reasonable time. This would be a great benchmark, but may be difficult to achieve? We propose not to make this a blocking point for this submission and simply reiterate to the authors the interest for readers of this additional work.

Authors' response:

We thank the reviewer for the thoughtful suggestion to benchmark against MedBERT and ClinicalBERT. While we agree that such a comparison could be informative if conducted properly, the primary focus of our study lies elsewhere. Our core contribution is the development of a model that computes patient-specific risk dynamically, updating predictions as new clinical information becomes available rather than relying on static snapshots of patient variables.
We also introduce a novel explainability module within the ETHOS framework, enabling real-time interpretability of evolving risk estimates. Although we evaluated our approach on the ED-MIMIC dataset to demonstrate performance on an established benchmark, this was intended as supportive rather than central. Our primary aim was to show that ETHOS is competitive on standard tasks while uniquely enabling adaptive, temporally resolved risk prediction, capabilities not readily addressed by existing models, including those typically used for benchmarking.
Even if another model were to match or slightly exceed our performance on static benchmarks, it would not diminish our main contribution: a dynamic, explainable model that adapts to evolving clinical data in real time. In the revised version we note in limitations, our benchmarking employed classical risk-prediction methods; more sophisticated approaches could yield higher benchmark scores.
We appreciate the reviewer's agreement that this is not a barrier to publication and take their feedback as encouragement to extend our benchmarking to additional models in future work.

Cookie Preference Center

We use cookies which are necessary to make our site work. We may also use additional cookies to analyze, improve and personalize our content and your digital experience. For more information, see our Cookie Policy.

You may choose not to allow some types of cookies. However, blocking some types may impact your experience of our site and the services we are able to offer. See the different category headings below to find out more or change your settings.

Allow all

Manage Consent Preferences

Strictly Necessary Cookies

Always active

These cookies are necessary for the website to function and cannot be switched off in our systems. They are usually only set in response to actions made by you which amount to a request for services, such as setting your privacy preferences, logging in or filling in forms. You can set your browser to block or alert you about these cookies, but some parts of the site will not then work.

Functional Cookies

These cookies enable the website to provide enhanced functionality and personalisation. They may be set by us or by third party providers whose services we have added to our pages. If you do not allow these cookies then some or all of these services may not function properly.

Performance Cookies

These cookies allow us to count visits and traffic sources so we can measure and improve the performance of our site. They help us to know which pages are the most and least popular and see how visitors move around the site.

Targeting Cookies

These cookies may be set through our site by our advertising partners. They may be used by those companies to build a profile of your interests and show you relevant adverts on other sites. If you do not allow these cookies, you will experience less targeted advertising.

Cookie List

Clear

Apply Cancel
Consent Leg.Interest

Confirm my choices

Powered by onetrust
