# Supplementary material for: Foundation model of electronic medical records for adaptive risk estimation
Source: Gigascience. 2025 Sep 30;14:giaf107. doi: 10.1093/gigascience/giaf107 (PMC12482913; doi:10.1093/gigascience/giaf107)
Supplement: giaf107_Reviewer_1_Report_Original_Submission — Jean-Luc Bosson -- 4/4/2025 [file giaf107_reviewer_1_report_original_submission.pdf]

Reviewer Report

Title: Foundation Model of Electronic Medical Records for Adaptive Risk Estimation

Version: Original Submission **Date:** 4/4/2025

Reviewer name: Jean-Luc Bosson

Reviewer Comments to Author:

This was peer reviewed jointly by Paul Antoine Beaudoin and Jean-Luc Bosson.
GIGASCIENCE REVIEW: Foundation Model of Electronic Medical Records for Adaptive Risk Estimation
This paper presents a new risk estimation system (ARES) capable of calculating real-time predictions throughout the patient journey in hospital thanks to an AI-based model using tokenized patient timelines (ETHOS). This new risk estimation system offers promising new possibilities and differs from other fixed time-points, which seem less flexible and agile.
On the general reading of the article, although the paper introducing the ETHOS framework is cited ([14]), it would be useful for the reader and the whole understanding of the article to provide more information about this framework in the introduction. As it stands, the reader is almost obliged to read the paper cited in [14] in order to understand the article.
A few major questions and reviews arise from our reading of the article:
- Firstly, from the clinical point of view :
o It seems that the most useful alert is the one given at least 12 to 24 hours before the event. ARES provide warnings in real-time, how would it be possible to evaluate its ability to predict events 24 hours before they occur? If an alert is too close to the predicted event, preventive measures cannot be taken. On the other hand, a prediction too close to the event risks using information that already reflects the event itself. There is therefore a risk of over-matching or artificially improving model quality. For example, predicting ICU admission knowing that the patient has just been intubated and ventilated makes no sense. Can the authors explain this point?
o What is the clinical justification of the composite indicator? This composite criterion combines highly heterogeneous events of varying importance (ie mortality and prolonged length of stay). We suggest deleting this criterion, which is clinically irrelevant and provides no major information on the method.
- With regard to methodology :
o It would be useful to detail a little more detail on the flowchart. In particular, what can explain the loss of over 10 thousands patients between the 299 721 patients of the MIMIC-IV database and the 285 622 PHTs after the tokenization process used in the study? Does it introduce a bias or risk of poorer generalisation of the method for future use?
- In terms of results,
o It seems important to clarify the new key advantages of ARES over ETHOS. For example, it is not clear whether the results on figure 5 are provided by ARES and ETHOS or only ETHOS as they are very close to those presented on the ETHOS publication cited in the references [14]
o On figure 4: the signal provided by ARES appears noisy due to the granularity of the tokens. What enables ARES to detect key breaks in the risk estimation timeline? Has token granularity been discussed

in the modelling process in order to provide less noisy timelines?
- Concerning the benchmarks provided to assess the results,
o It would be interesting to provide measures dedicated to imbalanced class classification (as it is the case) such as F1-score and balanced accuracy.
o Variable structure is very particular in the ETHOS framework due to tokenization. Would it be possible to provide more information about data pre-processing to run baselines like logistic regression with this particular data structure?
o Concerning the benchmarks, it would be interesting to provide an indicator of the energy consumption to train each of the algorithms and add it in the balance sheet.
We would also like to point out a few other minor reviews:
- On figure 4 : the colours differ between the legend and the figure itself
- The references are incomplete: we cannot see the names of journals.

Methods

Are the methods appropriate to the aims of the study, are they well described, and are necessary controls included? Choose an item.

Conclusions

Are the conclusions adequately supported by the data shown? Choose an item.

Reporting Standards

Does the manuscript adhere to the journal's guidelines on [minimum standards of reporting?]() Choose an item.

Choose an item.

Statistics

Are you able to assess all statistics in the manuscript, including the appropriateness of statistical tests used? Choose an item.

Quality of Written English

Please indicate the quality of language in the manuscript: Choose an item.

Declaration of Competing Interests

Please complete a declaration of competing interests, considering the following questions:

- Have you in the past five years received reimbursements, fees, funding, or salary from an organisation that may in any way gain or lose financially from the publication of this manuscript, either now or in the future?
- Do you hold any stocks or shares in an organisation that may in any way gain or lose financially from the publication of this manuscript, either now or in the future?
- Do you hold or are you currently applying for any patents relating to the content of the manuscript?
- Have you received reimbursements, fees, funding, or salary from an organization that holds or has applied for patents relating to the content of the manuscript?
- Do you have any other financial competing interests?
- Do you have any non-financial competing interests in relation to this paper?

If you can answer no to all of the above, write 'I declare that I have no competing interests' below. If your reply is yes to any, please give details below.

'I declare that I have no competing interests'

I agree to the open peer review policy of the journal. I understand that my name will be included on my report to the authors and, if the manuscript is accepted for publication, my named report including any attachments I upload will be posted on the website along with the authors' responses. I agree for my report to be made available under an Open Access Creative Commons CC-BY license (http://creativecommons.org/licenses/by/4.0/). I understand that any comments which I do not wish to be included in my named report can be included as confidential comments to the editors, which will not be published.

Choose an item.

To further support our reviewers, we have joined with Publons, where you can gain additional credit to further highlight your hard work (see: https://publons.com/journal/530/gigascience). On publication of this paper, your review will be automatically added to Publons, you can then choose whether or not to claim your Publons credit. I understand this statement.

Yes Choose an item.
