# Supplementary material for: Foundation model of electronic medical records for adaptive risk estimation
Source: Gigascience. 2025 Sep 30;14:giaf107. doi: 10.1093/gigascience/giaf107 (PMC12482913; doi:10.1093/gigascience/giaf107)
Supplement: giaf107_Reviewer_2_Report_Original_Submission — Heloisa Oss Boll -- 4/21/2025 [file giaf107_reviewer_2_report_original_submission.pdf]

Reviewer Report

Title: Foundation Model of Electronic Medical Records for Adaptive Risk Estimation

Version: Original Submission **Date:** 4/21/2025

Reviewer name: Heloisa Oss Boll

Reviewer Comments to Author:

The authors present ETHOS, a novel deep learning method for dynamic, personalized, and explainable clinical outcome prediction based on EHR. The approach is relevant and, in my view, holds strong potential for actual implementation in clinical settings.
Question to authors:
- Why is ETHOS currently restricted to ED settings? Could it generalize to inpatient/outpatient contexts with broader training?
- In "Analyses," please add a brief explanation about what the remaining 40% of PHTs represent.
- It seems all tasks can be framed as binary classifications. Have you explored ETHOS for multiclass or multilabel outcomes (diagnosis/ procedure codes, multiple LOS thresholds etc.)? How would thresholding work for overlapping events? If not, you could add a brief speculation in the Supplementary.
- Can you explain your data filtering approach for making predictions at admission level? E.g. truncation based on admission timestamp?
- How are thresholds determined for highlighting risk-contributing events?
- Was any usability testing or qualitative feedback from clinicians conducted to assess how predictions and explanations might be integrated into real workflows? It would be nice to see at least a speculation (Supplementary) of how the authors envision doctors using the system, e.g., 1 patient case.
- Would you consider integrating an LLM layer to perhaps summarize or explain risk-contributing tokens in natural language? For instance, using an LLM to return a phrase about a newly highlighted token, which understands the entire patient trajectory and that doctors can also query.
- How does explainability work if only one component from multi-component information is highlighted as important for a prediction?
- Consider adding AUPRC plots alongside AUC (Figure 5), given tasks are imbalanced.
- Add a brief explanation, at least in the Supplementary, about how baseline algorithms (e.g. Figure 3) work and differ from your method.
- The "Potential implications" section appears to be placeholder text, please revise.
- How do you potentially see the integration of clinical notes in the system?
- How does ETHOS handle variability in patient history length (e.g. patients with extensive vs. minimal prior data)? Has performance been evaluated across short and long PHTs?
- The token distribution shows extreme variation in both frequency and diversity across code groups. Could the authors elaborate on how the system handles this? Was synthetic data or oversampling considered to improve the representation of these less frequent events?
Minor revisions:

- Add a reference to support the claim in "The United States allocates nearly 18 percent of its GDP to healthcare, yet….".
- Add references to support the claim in "Similarly, conventional machine learning models depend on preselected predictors…".
- Provide the class balance for the four prediction tasks in the main paper.
- Add a figure, at least in the Supplementary, to exemplify patient data before/after tokenization. Even reusing the example provided in the MEDS paper could help clarify the technique.
- Add a brief explanation of the (HM+IA+PS) composite risk score in the "Analyses" section.
- In the "Probabilistic inference" section, fix (Section) in "…the estimated probability of mortality is calculated as M/N (Section)."

Methods

Are the methods appropriate to the aims of the study, are they well described, and are necessary controls included? Choose an item.

Conclusions

Are the conclusions adequately supported by the data shown? Choose an item.

Reporting Standards

Does the manuscript adhere to the journal's guidelines on [minimum standards of reporting?] Choose an item.

Choose an item.

Statistics

Are you able to assess all statistics in the manuscript, including the appropriateness of statistical tests used? Choose an item.

Quality of Written English

Please indicate the quality of language in the manuscript: Choose an item.

Declaration of Competing Interests

Please complete a declaration of competing interests, considering the following questions:

- Have you in the past five years received reimbursements, fees, funding, or salary from an organisation that may in any way gain or lose financially from the publication of this manuscript, either now or in the future?
- Do you hold any stocks or shares in an organisation that may in any way gain or lose financially from the publication of this manuscript, either now or in the future?

- Do you hold or are you currently applying for any patents relating to the content of the manuscript?
- Have you received reimbursements, fees, funding, or salary from an organization that holds or has applied for patents relating to the content of the manuscript?
- Do you have any other financial competing interests?
- Do you have any non-financial competing interests in relation to this paper?

If you can answer no to all of the above, write 'I declare that I have no competing interests' below. If your reply is yes to any, please give details below.

I declare that I have no competing interests.

I agree to the open peer review policy of the journal. I understand that my name will be included on my report to the authors and, if the manuscript is accepted for publication, my named report including any attachments I upload will be posted on the website along with the authors' responses. I agree for my report to be made available under an Open Access Creative Commons CC-BY license (http://creativecommons.org/licenses/by/4.0/). I understand that any comments which I do not wish to be included in my named report can be included as confidential comments to the editors, which will not be published.

Choose an item.

To further support our reviewers, we have joined with Publons, where you can gain additional credit to further highlight your hard work (see: https://publons.com/journal/530/gigascience). On publication of this paper, your review will be automatically added to Publons, you can then choose whether or not to claim your Publons credit. I understand this statement.

Yes Choose an item.
