# Supplementary material for: Foundation model of electronic medical records for adaptive risk estimation
Source: Gigascience. 2025 Sep 30;14:giaf107. doi: 10.1093/gigascience/giaf107 (PMC12482913; doi:10.1093/gigascience/giaf107)
Supplement: giaf107_Reviewer_3_Report_Original_Submission — Guishen Wang -- 4/26/2025 [file giaf107_reviewer_3_report_original_submission.pdf]

Reviewer Report

Title: Foundation Model of Electronic Medical Records for Adaptive Risk Estimation

Version: Original Submission **Date:** 4/26/2025

Reviewer name: Guishen Wang

Reviewer Comments to Author:

This manuscript introduces the Enhanced Transformer for Health Outcome Simulation (ETHOS) and the Adaptive Risk Estimation System (ARES), representing a novel approach to predicting critical healthcare outcomes using electronic health records (EHRs). The authors present a transformer-based foundation model that tokenizes patient health timelines (PHTs) to forecast future clinical trajectories and compute personalized risk probabilities for key outcomes such as mortality, ICU admission, and prolonged hospital stays. Notably, the system features an explainability module that highlights the clinical factors most influential in patient-specific risk assessments. The proposed approach is evaluated on the MIMIC-IV dataset, where it demonstrates superior predictive performance compared to traditional early warning systems and machine learning models.
However, I have several suggestions for the authors:
1. Bias and Generalizability: While the discussion of limitations is thoughtful, it would benefit from a more direct examination of potential biases present in the training dataâ€”such as demographic or institutional biasesâ€—and how these might affect the model's generalizability to diverse patient populations or external datasets.
2. Additional Limitations: Although the limitations of training on a single dataset are acknowledged, other important limitations are not fully explored. In particular, issues related to data missingness, temporal biases inherent in EHR data, and the potential for model drift over time should be discussed in greater detail.
3. Manuscript Formatting:The overall format of the manuscript could be further improved for readability and professionalism. For example, the abstract should be justify-aligned to enhance visual presentation and consistency with journal standards.

Methods

Are the methods appropriate to the aims of the study, are they well described, and are necessary controls included? Choose an item.

Conclusions

Are the conclusions adequately supported by the data shown? Choose an item.

Reporting Standards

Does the manuscript adhere to the journal's guidelines on [minimum standards of reporting?] Choose an item.

Choose an item.

Statistics

Are you able to assess all statistics in the manuscript, including the appropriateness of statistical tests used? Choose an item.

Quality of Written English

Please indicate the quality of language in the manuscript: Choose an item.

Declaration of Competing Interests

Please complete a declaration of competing interests, considering the following questions:

- Have you in the past five years received reimbursements, fees, funding, or salary from an organisation that may in any way gain or lose financially from the publication of this manuscript, either now or in the future?
- Do you hold any stocks or shares in an organisation that may in any way gain or lose financially from the publication of this manuscript, either now or in the future?
- Do you hold or are you currently applying for any patents relating to the content of the manuscript?
- Have you received reimbursements, fees, funding, or salary from an organization that holds or has applied for patents relating to the content of the manuscript?
- Do you have any other financial competing interests?
- Do you have any non-financial competing interests in relation to this paper?

If you can answer no to all of the above, write 'I declare that I have no competing interests' below. If your reply is yes to any, please give details below.

I declare that I have no competing interests' below.

I agree to the open peer review policy of the journal. I understand that my name will be included on my report to the authors and, if the manuscript is accepted for publication, my named report including any attachments I upload will be posted on the website along with the authors' responses. I agree for my report to be made available under an Open Access Creative Commons CC-BY license (http://creativecommons.org/licenses/by/4.0/). I understand that any comments which I do not wish to be included in my named report can be included as confidential comments to the editors, which will not be published.

Choose an item.

To further support our reviewers, we have joined with Publons, where you can gain additional credit to further highlight your hard work (see: https://publons.com/journal/530/gigascience). On publication of this paper, your review will be automatically added to Publons, you can then choose whether or not to claim your Publons credit. I understand this statement.

Yes Choose an item.
