# Supplementary material for: Foundation model of electronic medical records for adaptive risk estimation
Source: Gigascience. 2025 Sep 30;14:giaf107. doi: 10.1093/gigascience/giaf107 (PMC12482913; doi:10.1093/gigascience/giaf107)
Supplement: giaf107_Reviewer_4_Report_Original_Submission — W Jim Zheng -- 4/26/2025 [file giaf107_reviewer_4_report_original_submission.pdf]

Reviewer Report

Title: Foundation Model of Electronic Medical Records for Adaptive Risk Estimation

Version: Original Submission **Date:** 4/26/2025

Reviewer name: W Jim Zheng

Reviewer Comments to Author:

This manuscript presents a transformer-based modelâ€”Enhanced Transformer for Health Outcome Simulationâ€”and its application in adaptive risk estimation using EHR data. The model is evaluated on MIMIC-IV and benchmarked against a set of traditional and machine learning approaches. The authors position this work as a zero-shot foundation model for real-time, personalized clinical risk prediction. While the manuscript introduces a potentially impactful approach to clinical AI, several substantive concerns must be addressed before the work can be considered for publication. These primarily relate to methodological clarity, evaluation rigor, and support for the model's positioning as a "foundation model." The current version lacks sufficient technical and empirical depth to support the central claims. Specific concerns include:

1. Insufficient methodological detail: The manuscript omits key information about the model's training procedure and learning objective(s). It is unclear whether masked token prediction, autoregressive modeling, or another strategy was used. Details on the loss functions, training epochs, optimization strategy, and data preprocessing are essential to assess reproducibility and scalability.
2. Model and dataset scale: The model is trained on EHRs from approximately 300K patients, which is modest relative to other models such as MedBERT (trained on over 28 million patients). Similarly, the model's parameter count is relatively small. It is not evident that either the data scale or model capacity meets the bar typically associated with foundation models.
3. Benchmarking omissions: The comparative evaluation lacks transformer-based baselines trained on clinical data, such as MedBERT. Including such models would provide a more meaningful benchmark than comparisons with classical ML methods alone.
4. Exclusion of unstructured clinical text: The decision to exclude clinical notesâ€”a rich source of patient-level informationâ€”is a major limitation. The authors should discuss how this exclusion impacts performance and generalizability, particularly for a model intended to operate in real-world, zero-shot settings.
5. Evaluation methodology: The manuscript does not clarify whether all models were trained and evaluated on the same data splits, nor whether hyperparameter tuning or statistical testing was applied. This lack of rigor makes it difficult to interpret the significance of performance differences.
6. Zero-shot and "foundation model" claims: The manuscript emphasizes zero-shot capabilities but does not clearly contrast this with standard fine-tuning requirements in comparable models. Furthermore, the term "foundation model" is used without sufficient justification. Articulating the criteria used and how this model meets them (e.g., scale, generalizability, multi-task adaptability) would strengthen the framing.

In summary, the manuscript addresses an important and timely topic, but significant revisions are

needed to clarify technical details, expand the evaluation, and substantiate key claims. At this stage, I do not recommend acceptance, but I encourage the authors to address these concerns in a future submission.

Methods

Are the methods appropriate to the aims of the study, are they well described, and are necessary controls included? Choose an item.

Conclusions

Are the conclusions adequately supported by the data shown? Choose an item.

Reporting Standards

Does the manuscript adhere to the journal's guidelines on [minimum standards of reporting?]() Choose an item.

Choose an item.

Statistics

Are you able to assess all statistics in the manuscript, including the appropriateness of statistical tests used? Choose an item.

Quality of Written English

Please indicate the quality of language in the manuscript: Choose an item.

Declaration of Competing Interests

Please complete a declaration of competing interests, considering the following questions:

- Have you in the past five years received reimbursements, fees, funding, or salary from an organisation that may in any way gain or lose financially from the publication of this manuscript, either now or in the future?
- Do you hold any stocks or shares in an organisation that may in any way gain or lose financially from the publication of this manuscript, either now or in the future?
- Do you hold or are you currently applying for any patents relating to the content of the manuscript?
- Have you received reimbursements, fees, funding, or salary from an organization that holds or has applied for patents relating to the content of the manuscript?
- Do you have any other financial competing interests?
- Do you have any non-financial competing interests in relation to this paper?

If you can answer no to all of the above, write 'I declare that I have no competing interests' below. If your reply is yes to any, please give details below.

I declare that I have no competing interests

I agree to the open peer review policy of the journal. I understand that my name will be included on my report to the authors and, if the manuscript is accepted for publication, my named report including any attachments I upload will be posted on the website along with the authors' responses. I agree for my report to be made available under an Open Access Creative Commons CC-BY license (http://creativecommons.org/licenses/by/4.0/). I understand that any comments which I do not wish to be included in my named report can be included as confidential comments to the editors, which will not be published.

Choose an item.

To further support our reviewers, we have joined with Publons, where you can gain additional credit to further highlight your hard work (see: https://publons.com/journal/530/gigascience). On publication of this paper, your review will be automatically added to Publons, you can then choose whether or not to claim your Publons credit. I understand this statement.

Yes Choose an item.
